# WGS reveals high-risk clones of *Pseudomonas aeruginosa* harbouring extensive antimicrobial and predicted anti-phage defense systems recovered from diabetic foot ulcer patients in Egypt

**DOI:** 10.1186/s12866-026-05145-x

**Published:** 2026-06-03

**Authors:** Mai A. Amer, Manal M. Darwish, Reham Monir, Ahmed Al Taweel, Ayat I. Ghanem, Ihab N. Hanna, Aya H. Hefnawy, Nada S. Gebreel, Noha M. Soltan, Yara H. Aboudewan, Samira M. Hamed

**Affiliations:** 1 Microbiology and Immunology Department, October University for Modern Sciences and Arts (MSA), Giza, Egypt; 2 Medical Microbiology and Immunology, Faculty of Medicine, Ain Shams University, Cairo, Egypt; 3 National Institute of Diabetes and Endocrinology (NIDE), Cairo, Egypt; 4 PharmD Students Program, Faculty of Pharmacy, October University for Modern Sciences and Arts (MSA), Giza, Egypt

**Keywords:** Diabetic foot ulcer, *Pseudomonas aeruginosa*, Whole-genome sequencing, Multidrug resistance, Anti-phage Defense systems

## Abstract

Diabetic foot ulcers (DFUs) are a major complication of diabetes mellitus and a leading cause of morbidity, amputation, and mortality among affected patients. In this study, five *Pseudomonas aeruginosa* isolates recovered from DFUs of diabetic patients in Egypt were investigated. Antimicrobial susceptibility was evaluated using the disc diffusion method and broth microdilution assay, while biofilm-forming ability was phenotypically assessed using the crystal violet assay. All isolates exhibited multidrug-resistant (MDR) phenotypes, with two isolates classified as pan-drug resistant (PDR). Moreover, all isolates demonstrated a strong capacity for biofilm formation. Whole-genome sequencing (WGS) and subsequent bioinformatic analysis revealed three sequence types, ST369, ST664, and ST773, corresponding to serotypes O6, O2, and O11, respectively, with ST664 and ST773 representing high-risk clones. These high-risk clones carried horizontally transferable integrative conjugative elements (ICEs) encoding metal resistance and anti-phage defense systems. The isolates also harboured a diverse resistome comprising *bla*_NDM-1_, *bla*_PER-1_, *rmtB*, *qnrVC1*, *aac(3)*, *ant(4')-IIb*, *aph(3')-VIb*, *sul1*, *tet(G)*, and *cmlA9* in addition to biocide resistance genes (*qacE*, *triABC*). Colistin resistance in three isolates was associated with L71R *pmrA* mutation. Virulence profiling revealed a minimum of 230 genes associated with adhesion, biofilm formation, toxin production, and secretion systems. Notably, high-risk clones (ST773 and ST664) carried an extensive array of anti-phage defense systems, up to 32 per genome, potentially reducing the efficacy of bacteriophage-based therapeutic alternatives. Collectively, these findings highlight the remarkable genomic plasticity and adaptive resilience of *P. aeruginosa* in chronic diabetic wounds, emphasizing the urgent need for improved infection control practices, genomic surveillance, and the development of novel therapeutic strategies for effective DFU management. To our knowledge, this is the first genomic characterization of MDR *P. aeruginosa* isolates from DFUs in Egypt.

**Supplementary Information:**

The online version contains supplementary material available at 10.1186/s12866-026-05145-x.

## Introduction

*Pseudomonas* spp. are non-fermenting Gram-negative rods widely distributed in diverse environments such as soil, water, and healthcare settings, and are notable for their intrinsic resistance to multiple antibiotics [48, 77]. Among them, *Pseudomonas aeruginosa* is the most clinically important species and is a major cause of chronic and opportunistic infections, particularly diabetic foot ulcers (DFUs) [37, 89, 94], where it contributes to poor healing outcomes and increased risk of amputation. Its metabolic versatility and ability to persist in hospital environments make it a significant nosocomial pathogen and a member of the ESKAPE group, which includes highly drug-resistant organisms responsible for healthcare-associated infections [33, 38, 124]. Resistance in *P. aeruginosa* is driven by multiple mechanisms, including efflux pumps, porin modifications, beta-lactamase production, and strong biofilm formation, all of which enhance survival in chronic infections [15, 40, 55]. Based on antimicrobial susceptibility profiles, *P. aeruginosa* isolates are classified as MDR, XDR, DTR, or PDR, with increasing reports of XDR and PDR strains complicating treatment [28, 65]. In DFUs, biofilm-associated growth further exacerbates resistance, as biofilm-embedded cells can exhibit dramatically increased tolerance to antibiotics compared to planktonic bacteria, leading to persistent infection and reduced therapeutic success [57, 71].

This expanding resistance spectrum reflects the complex interplay of intrinsic, adaptive, and acquired resistance mechanisms, which collectively confer reduced susceptibility to multiple antimicrobial classes. As a result of this extensive resistance repertoire, *P. aeruginosa* has been designated by the World Health Organization as a priority 1 “critical” pathogen, underscoring the urgent need for intensified research efforts and confirmatory development of novel therapeutic strategies [78].

*P. aeruginosa* is an important opportunistic pathogen in DFU infections, with multiple epidemiological studies and meta-analyses reporting a global prevalence of approximately 16–21%. A PRISMA-based systematic review estimated its overall prevalence at 16.6%, with nearly 37.9% of isolates being multidrug-resistant, and highlighted significant regional variation across Asia, Africa, and Western countries [28, 52, 65]. Although Gram-positive bacteria, particularly *Staphylococcus aureus*, remain the most frequently isolated pathogens in DFUs [52], *P. aeruginosa* is consistently recognized as one of the leading Gram-negative organisms alongside *Escherichia coli* [131]. Importantly, despite its moderate prevalence, *P. aeruginosa* is strongly associated with severe, chronic, and non-healing infections due to its intrinsic multidrug resistance and potent biofilm-forming ability, which collectively contribute to treatment failure and poor clinical outcomes [52].

Managing infections caused by MDR/XDR *P. aeruginosa* is challenging and often requires newer β-lactam/β-lactamase inhibitor combinations such as ceftolozane/tazobactam and ceftazidime/avibactam. Due to limited options, older antibiotics like colistin have been reintroduced as last-resort treatments [42]. However, increased reliance on colistin has led to the emergence and spread of resistant strains, driven by horizontal gene transfer and chromosomal mutations, posing a significant public health threat [95]. The dissemination of colistin resistance is further exacerbated by horizontal gene transfer (HGT) mediated by conjugative plasmids and mobile genetic elements (MGEs), as well as vertical inheritance through chromosomal mutations, posing a serious public health challenge, especially among XDR and PDR clinical isolates [1].

Advancements in whole-genome sequencing (WGS) have revealed novel resistance determinants and highlighted the clonal dissemination of high-risk MDR *P. aeruginosa strains* in diabetic foot infections [74, 123]. These findings highlight the urgent need for alternative therapeutic approaches, including bacteriophage therapy, antimicrobial peptides, and novel adjuvant therapies to combat antibiotic resistance in *P. aeruginosa* [45, 56, 30, 98].

Bacteriophages have recently gained attention as promising therapeutic agents for treating bacterial infections. To defend themselves, bacteria have developed a variety of protective mechanisms that are often grouped together in their genomes, forming defense islands. In recent years, researchers have discovered more than a hundred new anti-phage defense systems, revealing the complexity of bacterial immunity. Among these is the Hachiman system, a remarkable and recently identified defense mechanism present in about 4% of bacterial genomes across nearly 2,000 species, which provides a broad protection against diverse phage. Identifying this system, particularly in *P. aeruginosa*, may offer important understanding into how bacteria resist phage attacks and how this could influence future phage therapy strategies [26].

Research on *P. aeruginosa* in DFU has evolved from prevalence studies to genomics-driven insights into virulence, biofilms, and resistance. The present study employed whole-genome sequencing to characterize MDR *P. aeruginosa* isolates from Egyptian DFU patients, providing new insights into resistance, virulence, and potential barriers to emerging therapies, and supporting improved surveillance and therapeutic strategies.

## Materials and methods

### Collection and preliminary identification of bacterial isolates

Five MDR *P. aeruginosa* isolates were obtained from patients with infected DFUs admitted to the National Institute of Diabetes and Endocrinology (NIDE), Cairo, Egypt, between February and March 2024. Bacterial isolates were initially recovered and identified at the Microbiology Laboratory of the NIDE as part of routine clinical care. Confirmed *P. aeruginosa* isolates were preserved in glycerol stocks and transported under cold chain conditions (4 °C) to the Microbiology Laboratory at MSA University to maintain viability and genetic stability, where further phenotypic and molecular analysis were conducted. Species identification was performed using conventional biochemical methods in combination with matrix-assisted laser desorption/ionization time-of-flight mass spectrometry (MALDI-TOF–MS) in Children’s Cancer Hospital Egypt (57,357), Cairo, Egypt. (BioMérieux; Marcy l’Etoile, France). The demographic and clinical details of the DFU samples are summarized in Table 1.

**Table 1:** The demographic and clinical details of the *P. aeruginosa* DFU Isolates

Isolate ID	Collection Date	Patient Age (years)	Gender
DFU7	February 2024	52	Female
DFU9	February 2024	56	Male
DFU16	February 2024	66	Male
DFU48	March 2024	72	Female
DFU58	March 2024	65	Male

### Antimicrobial susceptibility testing

The susceptibility of the five isolates to a large panel of antimicrobial agents was evaluated using the Kirby-Bauer disc diffusion method. The following antibiotic discs (Oxoid, UK) were utilized: amoxicillin/clavulanic acid (20/10 μg), piperacillin (100 μg), piperacillin/tazobactam (100/10 μg), cefazolin (30 μg), cefoxitin (30 μg), ceftriaxone (30 μg), cefotaxime (30 μg), cefepime (30 μg), ceftazidime (30 μg), aztreonam (30 μg), meropenem (10 μg), gentamicin (10 µg), amikacin (30 μg), ciprofloxacin (5 μg), tetracycline (30 μg), tigecycline (15 μg), chloramphenicol (30 μg), nitrofurantoin (300 μg), fosfomycin (200 μg), and trimethoprim/sulfamethoxazole (1.25/23.75 μg). Additionally, the broth microdilution assay was employed to determine the minimum inhibitory concentrations (MICs) of colistin (Sigma-Aldrich, St Louis, MO, USA) across a concentration range of 128–0.125 μg/mL. All susceptibility tests were conducted and interpreted according to the Clinical and Laboratory Standards Institute (CLSI M100; CLSI M07) guidelines [34, 35] for all antimicrobial agents, except for tigecycline, for which the susceptibility breakpoints recommended by EUCAST v14.0 for *Enterobacterales* were applied [47].

### Phenotypic characterization of biofilm production of *P. aeruginosa* isolates

The biofilm-forming capacity of the isolates was assessed using the crystal violet (CV) staining assay [97]. Overnight bacterial cultures were diluted to a concentration of 10^5^ CFU/mL and inoculated into 200 μL of tryptic soy broth (TSB) supplemented with 1% (v/v) glucose in a 96-well flat-bottom polystyrene microtiter plate (Greiner Bio-one^®^, Germany). The plate was incubated at 37 °C for 24 h without shaking. Following incubation, turbidity at 600 nm was measured to assess microbial growth. The wells were then emptied, rinsed twice with sterile phosphate-buffered saline (PBS), and left to air dry. Dried biofilms were stained with 0.1% crystal violet for 15 min. Excess stain was discarded, and the wells were washed with 200 μL of sterile distilled water, followed by air drying. Finally, 33% glacial acetic acid was added to solubilize the retained dye. The biofilm biomass was then measured at 545 nm. Biofilm formation was assessed using the biofilm formation index (BFI), calculated as

$$BFI=\frac{(\mathrm{O}\mathrm{D}545 \mathrm{o}\mathrm{f} \mathrm{c}\mathrm{r}\mathrm{y}\mathrm{s}\mathrm{t}\mathrm{a}\mathrm{l} \mathrm{v}\mathrm{i}\mathrm{o}\mathrm{l}\mathrm{e}\mathrm{t} \mathrm{s}\mathrm{t}\mathrm{a}\mathrm{i}\mathrm{n}\mathrm{e}\mathrm{d} \mathrm{m}\mathrm{i}\mathrm{c}\mathrm{r}\mathrm{o}\mathrm{b}\mathrm{i}\mathrm{a}\mathrm{l} \mathrm{c}\mathrm{e}\mathrm{l}\mathrm{l}\mathrm{s}- \mathrm{O}\mathrm{D}545 \mathrm{o}\mathrm{f} \mathrm{s}\mathrm{t}\mathrm{a}\mathrm{i}\mathrm{n}\mathrm{e}\mathrm{d} \mathrm{b}\mathrm{l}\mathrm{a}\mathrm{n}\mathrm{k} \mathrm{w}\mathrm{e}\mathrm{l}\mathrm{l}\mathrm{s})}{(\mathrm{O}\mathrm{D}600 \mathrm{o}\mathrm{f} \mathrm{t}\mathrm{h}\mathrm{e} \mathrm{b}\mathrm{a}\mathrm{c}\mathrm{t}\mathrm{e}\mathrm{r}\mathrm{i}\mathrm{a}\mathrm{l} \mathrm{c}\mathrm{u}\mathrm{l}\mathrm{t}\mathrm{u}\mathrm{r}\mathrm{e}-\mathrm{O}\mathrm{D}600 \mathrm{o}\mathrm{f} \mathrm{t}\mathrm{h}\mathrm{e} \mathrm{b}\mathrm{l}\mathrm{a}\mathrm{n}\mathrm{k} \mathrm{c}\mathrm{o}\mathrm{n}\mathrm{t}\mathrm{r}\mathrm{o}\mathrm{l})}$$

Based on BFI values, isolates were classified into four categories: non-adherent (BFI < 0.35), weak (0.35–0.69), moderate (0.70–1.09), and strong biofilm producers (> 1.10), according to the defined semi-quantitative criteria [9].

### Genomic analysis using whole-genome sequencing

DNA extraction was done using QIAamp DNA Kits (Qiagen, Hilden, Germany), following the manufacturer’s protocol. Library preparation and WGS were performed by BGI Tech Solutions (Hong Kong) on the DNBseq™ platform using a paired-end 150 bp (PE150) sequencing strategy. Raw reads underwent routine quality filtering and adaptor removal with SOAPnuke [32].

High-quality reads were assembled de novo through the BV-BRC (https://www.bv-brc.org/) assembly service. Unicycler v0.4.8. [104] was used for genome assembly, while PlasmidSPAdes [10] was employed for plasmid assembly. Draft genomes were annotated using the NCBI Prokaryotic Genome Annotation Pipeline [125]. Circular genome diagrams were generated via Proksee (https://proksee.ca/).

For taxonomic and phylogenomic analyses, full-length 16S rRNA gene sequences retrieved from the assemblies were deposited in NCBI (www.ncbi.nlm.nih.gov) with accession numbers as follows: DFU7: PV082531, DFU9: PV083167, DFU16: PV083168, DFU48: PV082535, and DFU58: PV082532, and compared with reference taxa [29]. BLASTn searches against curated 16S rRNA databases were carried out [27].

Genome-based classification was further assessed using the Type Strain Genome Server (TYGS) with MASH-based similarity calculations (https://tygs.dsmz.de/) [105], digital DNA–DNA hybridization thresholds for species/subspecies delineation, and a tetra-correlation search via JSpeciesWS [116].

A codon-based phylogenomic tree of the most closely related *P. aeruginosa* genomes was generated using BV-BRC’s Codon Tree workflow [104]. The tree was then visualized and annotated using iTOL (https://itol.embl.de/) [80].

Strain typing was conducted in silico. The multilocus sequence types (MLST) profiles were assigned via the PubMLST *P. aeruginosa* database, which follows the MLST scheme established by Juneja and Lazzaro [69], which is based on comparing the internal fragments of the seven housekeeping genes: *acsA*, *aroE*, *guaA*, *mutL*, *nuoD*, *ppsA*, and *trpE*.

In silico O-serotyping was conducted using PAst, a bioinformatics tool that enables rapid and accurate serotyping of *P. aeruginosa* based on WGS data (https://cge.cbs.dtu.dk/services/PAst-1.0) [126]. The assembled genomes were analyzed against a curated database of O-specific antigen (OSA) gene clusters using BLASTn. Serogroups were assigned when the alignment coverage of an OSA cluster within a genome exceeded 95%, indicating a reliable match to a specific O-serotype.

Antimicrobial resistance determinants were detected using the Resistance Gene Identifier (RGI) from the CARD database [5]. Mutations associated with fluoroquinolone and colistin resistance were investigated by aligning *gyrA*, *parC*, *pmrA*, *pmrB*, and *phoQ* against *P. aeruginosa* reference sequences (PAO1 and ATCC^®^ 33,363) using BV-BRC alignment utilities and Clustal Omega (https://www.ebi.ac.uk/Tools/msa/clustalo/) and visualized with the MView 1.63 alignment viewer (https://www.ebi.ac.uk/Tools/msa/mview/). Virulence genes were catalogued using VFanalyzer [84], while pathogenicity prediction was assessed with PathogenFinder that was used to predict the pathogenicity of the isolates for human hosts (https://cge.cbs.dtu.dk/services/PathogenFinder/) [31]. Gene neighborhoods surrounding resistance/virulence genes were visualized using SnapGene (http://www.snapgene.com).

MGEs were examined using MobileElementFinder (https://cge.cbs.dtu.dk/services/ MGE/) [66] and VRprofile [82]. Insertion sequences (ISs) were identified by comparison to ISFinder (http://www-is.biotoul.fr) [81]. Genomic islands (GI) were predicted using IslandViewer4 after mapping draft genomes against the genome of *P. aeruginosa* strain PAO1 (GenBank accession: NC_002516.2) using (http://www.pathogenomics.sfu.ca/islandviewer/) [19]. Integrative and conjugative elements (ICEs) were detected using ICEfinder [20], and integrons were located with IntegronFinder v2.0 [100]. Prophage regions were screened using PHASTER [12].

Defense systems were identified using DefenseFinder (v1.2.3) (https://defensefinder.mdmlab.fr/) and PADLOC (v2.0.0) (https://padloc.otago.ac.nz/) with default parameters [26, 68]. The presence of CRISPR-Cas systems was assessed using CRISPRCasTyper (https://crisprcastyper.crispr.dk).

## Results

### Preliminary identification of the bacterial isolates

Isolates were identified using biochemical reactions and showed characteristic results supporting their identification as *P. aeruginosa*. The isolates were oxidase- and catalase-positive and produced a distinctive blue-green pigment on nutrient agar. It grew on cetrimide agar, exhibiting a distinct odour and fluorescent pigmentation. The isolates utilized citrate as a sole carbon source. The Triple Sugar Iron (TSI) agar test showed a non-fermentative reaction and negative hydrogen sulphide (H₂S) production and gas formation. Negative results were observed for urease, indole, methyl red, Voges-Proskauer tests. These biochemical characteristics were collectively confirmed by the identification using MALDI-TOF–MS [7].

### Antimicrobial susceptibility profiles of *P. aeruginosa* isolates

The antimicrobial susceptibility testing revealed a broad resistance profile among the *P. aeruginosa* isolates across multiple antibiotic classes. Although a wide panel of antibiotics was included to characterize overall resistance patterns, interpretation of clinically relevant resistance was primarily based on anti-pseudomonal agents, particularly ceftazidime, cefepime, carbapenems, aminoglycosides, and fluoroquinolones. Some antibiotics were included in the screening panel, such as tetracycline, tigecycline, and several non-anti-pseudomonal cephalosporins, have limited or no clinical efficacy against *P. aeruginosa* and were therefore used for comparative intrinsic resistance profiling rather than therapeutic evaluation. DFU9, DFU16, and DFU48 isolates were identified as MDR, showing resistance to at least one antibiotic in three or more different antimicrobial categories. Furthermore, DFU7 and DFU58 were PDR; resistant to all tested antimicrobial agents, in accordance with the MDR phenotype criteria defined by Magiorakos et al. [88]. The complete antimicrobial susceptibility profiles of the isolates are presented in Table 2.

**Table 2:** Antimicrobial susceptibility profiles of *P. aeruginosa* isolates. Susceptibility results are interpreted as susceptible (S), intermediate (I), or resistant (R)

Antimicrobial Class	Antimicrobial Agents	Antimicrobial Susceptibility				
		**DFU7**	**DFU9**	**DFU16**	**DFU48**	**DFU58**
Penicillins	Amoxicillin/clavulanic acid	R	R	R	R	R
	Piperacillin	R	R	R	R	R
	Piperacillin/tazobactam	R	R	R	R	R
Cephalosporins	Cefazolin	R	R	R	R	R
	Cefoxitin	R	R	R	R	R
	Ceftazidime	R	R	R	S	R
	Ceftriaxone	R	R	R	R	R
	Cefepime	R	R	I	I	R
	Cefotaxime	R	R	R	S	R
Monobactams	Aztreonam	R	S	S	S	R
Carbapenems	Meropenem	R	I	I	I	R
Lipopeptides	Colistin*	R (≥ 512)	R (≥ 4)	R (≥ 512)	R (≥ 512)	R (≥ 512)
Aminoglycosides	Amikacin	R	S	S	S	R
	Gentamicin	R	S	R	S	R
	Tobramycin	R	R	R	R	R
Tetracyclines	Tetracycline	R	R	R	R	R
	Tigecycline	R	R	R	R	R
Fluoroquinolones	Ciprofloxacin	R	S	I	R	R
Folate pathway antagonists	Trimethoprim/sulfamethoxazole	R	R	R	I	R
Phenicols	Chloramphenicol	R	R	R	S	R
Fosfomycins	Fosfomycin	R	R	R	R	R
Nitrofurans	Nitrofurantoin	R	R	R	R	R

^*^Colistin MIC was determined by broth microdilution assay

### Phenotypic characteristics of biofilm production of *P. aeruginosa* isolates

**Fig. 1:**
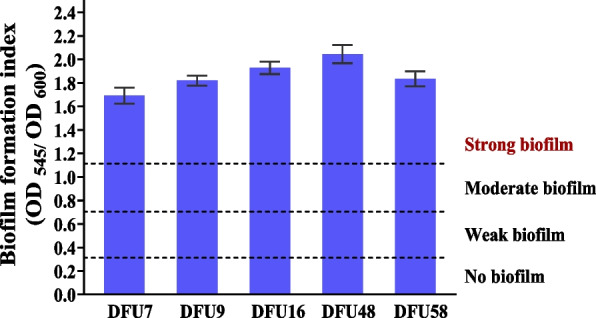
Biofilm formation ability of the *P. aeruginosa* isolates using crystal violet assay. Isolates were classified according to BFI into four categories: none (< 0.35), weak (0.35 to 0.69), moderate (0.70 to 1.09), and strong (> 1.10)

Biofilm formation ability of *P. aeruginosa* isolates was tested using the crystal violet biofilm assay. All five *P. aeruginosa* isolates recovered from DFU were able to form strong biofilm (Fig. 1).

### Genome assembly and annotation metrics

Draft genomes were assembled from short-read sequencing data, yielding between 47 and 170 contigs per isolate, with an average N_50_ of 230,789 bp. Detailed genome and assembly statistics, including genome size, contig count, RNA genes, GC content, number of coding sequences, N50, and L50, are provided in Supplementary Table 1. The draft genome sizes of the isolates ranged from 6.3 to 7.1 Mb, with GC content between 65.8% and 66.4%. WGS identified the five isolates as *P. aeruginosa*. Regarding genome quality, the genome completeness ranged between 99.1% to 99.9%, with contamination levels ranging between 1 to 2%. Annotation features showed a total of 6,727 genes in DFU7, 5,967 genes in DFU9, 5,973 genes in DFU16, 6,792 genes in DFU48, and 6,974 genes in DFU58, including 5,202, 4,843, 4,848, 5,192, and 5,369 protein-coding CDSs, respectively. All the isolates contained 59 tRNA and 2 rRNA, except DFU58, which contained 60 tRNA and 3 rRNA. The functional annotation statistics of the draft genomes are shown in Supplementary Table 1.

### WGS-based identification

The full-length 16S rRNA gene sequences of the isolates were analysed using the EzBioCloud 16S-based ID tool. Isolates DFU7 and DFU48 showed 99.93% similarity, while DFU9, DFU16, and DFU58 showed 100% similarity to *P. aeruginosa* JCM 5962ᵀ (BAMA01000316). Further alignment against the NCBI rRNA_typestrains/16S_ribosomal_RNA database revealed that all five isolates had the highest similarity to *P. aeruginosa* strain DSM 50071 (NR_117678.1), with 99% sequence coverage and identity values ranging from 99.87% to 99.93%. In each case, *P. aeruginosa* strain ATCC 10145 (NR_114471.1) was identified as the second closest match, showing 97% coverage and identity values between 99.93% and 100%. The phylogenomic tree presented in Supplementary Fig. 1, which includes the five *P. aeruginosa* alongside type strains from the TYGS database, demonstrates that all the isolates belong to the species *P. aeruginosa*, and the closest type strain is strain DMS50071.

### Genome-based phylogeny

**Fig. 2:**
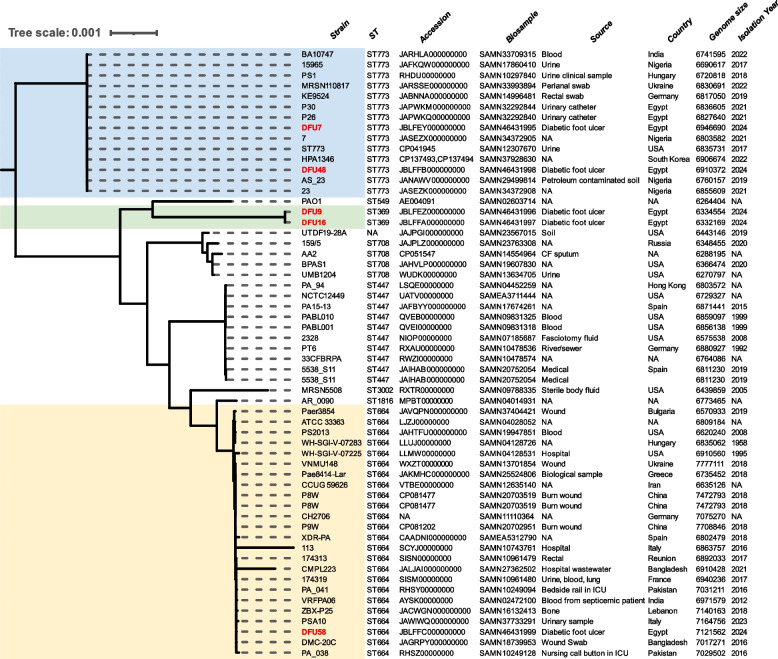
Whole-genome–based phylogenetic tree of *P. aeruginosa* isolates. The *P. aeruginosa* genomes sequenced in this study are labelled in red, while closely related genomes obtained from public databases are highlighted in blue (ST773), green (ST369), and yellow (ST664). The phylogenetic tree was constructed using BV-BRC and visualized with the iTOL web tool (v6.7; https://itol.embl.de/). Closely related publicly available genomes retrieved from BV-BCRC database are included for comparative analysis, with associated metadata (including accession number, biosample number, isolation source, country, enome size and year of collection) displayed to support epidemiological interpretation

The phylogenetic tree in Fig. 2 illustrates the genomic relatedness of the study isolates in comparison to closely related genomes retrieved from the BV-BRC database (Supplementary Tables 2). This analysis was conducted to explore the epidemiological relationships among the isolates, revealing their genetic relatedness and potential transmission patterns within the broader context of global *P. aeruginosa* strains.

### MLSTs and predicted serotypes

MLST analysis identified three distinct sequence types (STs) among the isolates, including two high-risk clones, ST773 and ST664. Isolates DFU7 and DFU48 belonged to ST773 and were predicted to be serotype O11, while isolates DFU9 and DFU16 were assigned to ST369 and associated with serotype O6. The remaining isolate, DFU58, was identified as ST664 and predicted to belong to serotype O2. These findings indicate the coexistence of multiple high-risk lineages within DFU infections, highlighting the genetic diversity and epidemiological relevance of the recovered isolates.

### Resistome analysis

Genome analysis of our isolates revealed a diverse array of AMR genes, efflux systems, and regulatory elements contributing to their MDR phenotypes (Table 3). Efflux systems were particularly abundant among the isolates. All genomes possessed at least ten chromosomally encoded Resistance-Nodulation-Division (RND) family multidrug efflux pump clusters, including MexAB-OprM, MexCD-OprJ, MexEF-OprN, MexGHI-OpmD, MexJK-OpmH, MexMN-OprM, MexPQ-OpmE, MexXY-OprM, MexVW-OprM, and MuxABC-OpmB. Additionally, the *cmlA9* and *tet*(G) genes, both encoding Major Facilitator Superfamily (MFS) efflux pumps, were detected in isolates DFU7, DFU48, and DFU58.

**Table 3:** AMR genes carried by *P. aeruginosa* isolates

Antimicrobial class	AMR Genes				
	DFU7 (ST773)	DFU9 (ST369)	DFU16 (ST369)	DFU48 (ST773)	DFU58 (ST664)
ß-lactams	*bla*_OXA-395_*,* *bla*_PDC-16_, *bla*_PER-1_, bla_NDM-1_	*bla*_OXA-494_, *bla*_PDC-60_	*bla*_OXA-494_, *bla*_PDC-60_	*bla*_OXA-395_, *bla*_PDC-16_, *bla*_PER-1_, *bla*_NDM-1_	*bla*_OXA-50_, *bla*_OXA-14_, *bla*_PDC-98_
Aminoglycosides	*aph(3'')-Ib, aph(3')-IIb, aph(3')-VIb,* *ant(3'')-Ia, (aadA11),* *aac(3),* *rmtB,* *emrE*	*aph(3')-IIb,* *emrE*	*aph(3')-IIb,* *emrE,*	*aph(3'')-Ib, aph(3')-IIb, aph(3')-VIb,* *ant(3'')-Ia, (aadA11),* *aac(3),* *rmtB,* *emrE*	*aph(3')-IIb,* *ant(4')-IIb,* *emrE*
Fluoroquinolones	*qnrVC1*	–-	–-	*qnrVC1*	–-
Sulfonamides	*sul1*	–-	–-	*sul1*	–-
Phenicols	*catB7, cmlA9*	*catB7*	*catB7*	*catB7, cmlA9*	*catB7, cmlA9*
Fosfomycin	*fosA*	*fosA*	*fosA*	*fosA*	*fosA*
Tetracyclines	*tet(G)*	–-	–-	*tet(G)*	–-
Disinfectants	*triABC*	*triABC*	*triABC*	*triABC*	*triABC, qacE*

In addition to intrinsic β-lactamase genes, including members of the class-D β-lactamase *bla*_OXA-50_ family (*bla*_OXA-50_, *bla*_OXA-395_, and *bla*_OXA-494_) and the class-C β-lactamase *Pseudomonas*-derived cephalosporinase genes (*bla*_PDC_), acquired β-lactamase genes were also identified. The carbapenem-hydrolysing metallo-β-lactamase-coding gene *bla*_NDM-1_ was detected in two carbapenem-resistant isolates belonging to sequence type ST773 (DFU7 and DFU48). Notably, the ST773 isolates carried four β-lactamase genes (*bla*_OXA-395_, *bla*_PDC-16_, *bla*_PER-1_, and *bla*_NDM-1_), while ST369 (DFU9 and DFU16) harbored two genes (*bla*_OXA-494_ and *bla*_PDC-60_). The ST664 isolate (DFU58) contained *bla*_OXA-50_, *bla*_OXA-14_, and *bla*_PDC-98_. Collectively, these enzymes confer resistance to a broad spectrum of β-lactam antibiotics, including carbapenems and extended-spectrum cephalosporins.

Analysis of quinolone- and polymyxin-resistance–associated genes in the *P. aeruginosa* isolates revealed several point mutations in *gyrA*, *parC*, *pmrA*, and *pmrB*, while no mutations were detected in *phoQ*, as shown in Table 4.

**Table 4:** *P. aeruginosa* isolates mutations in *gyrA, parC, pmrA,* and *pmrB* genes

Isolate	*gyrA*	*parC*	*pmrA*	*pmrB*				
DFU7	T83I	S87L	–-	S2P	A4T	V15I	G68S	Y345H
DFU9	–-	–-	L71R	–-	–-	–-	–-	–-
DFU16	–-	–-	L71R	–-	–-	–-	–-	–-
DFU48	T83I	S87L	–-	S2P	T4A	V15I	G68S	Y345H
DFU58	T83I	S87L	L71R	–-	–-	–-	–-	Y345H

Since sequence type (ST)-related polymorphisms in the *pmrA* and *pmrB* genes have been previously reported [135], multiple sequence alignment (MSA) of the predicted amino acid sequences from our isolates was performed against those from other strains sharing the same STs, as well as the reference strain *P. aeruginosa* PAO1. The alignments were visualized using MView (Supplementary Figs. 2–6).

### Pathogenicity and virulome

**Fig. 3:**
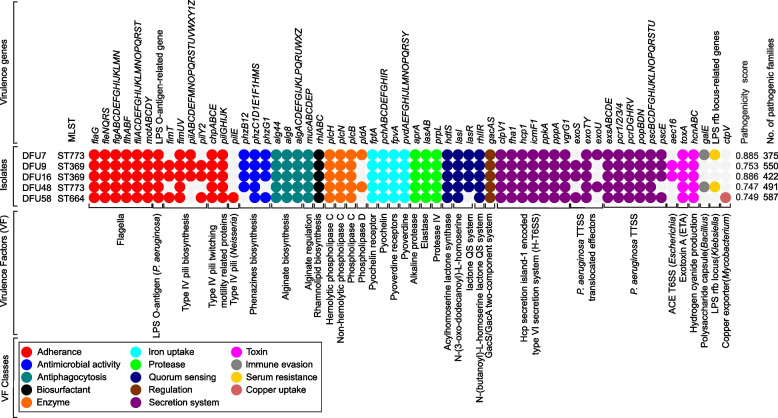
Heatmap illustrating the distribution of major virulence factor genes among five *P. aeruginosa* isolates. The presence of each gene is represented by a colored circle. Each isolate is annotated with its corresponding pathogenicity score and the number of pathogenic gene families as predicted by PathogenFinder

According to the PathogenFinder analysis, isolate DFU58 harbored the highest number of proteins matching pathogenic protein families, followed by DFU9, DFU48, DFU16, and DFU7. BLAST comparison of the draft genomes against the VFDB enabled the detection of genes linked to experimentally validated virulence traits. Key virulence factors related to adherence, antimicrobial activity, antiphagocytosis, biosurfactant production, enzyme secretion, iron uptake, protease production, quorum sensing, regulation, secretion systems, toxins, immune evasion, and serum resistance were detected (Fig. 3).

While most virulence factors were conserved across all isolates, variation was observed in several virulence factors, including type IV pili, phenazine biosynthesis, phospholipase D, the N-(3-oxo-dodecanoyl)-L-homoserine lactone quorum-sensing system, and the type III secretion system (TTSS). Additionally, certain virulence factors typically associated with other species were identified in some but not all isolates, including the ACE type VI secretion system (T6SS) from *Escherichia* sp., the polysaccharide capsule from *Bacillus* sp., the LPS rfb locus from *Klebsiella* sp., and the copper exporter from *Mycobacterium* sp.

### Mobile genetic elements

Comprehensive genomic screening revealed the presence of multiple MGEs in our *P. aeruginosa* isolates, including integrons, transposons, and ISs carrying antimicrobial and heavy metal resistance determinants, as shown in Table 5.

**Table 5:** MGEs in *P. aeruginosa* isolates linked to antibiotic resistance genes

Isolate	Contig	MGEs	Associated resistance genes	Origin Species
DFU7	1	IS*Pa100*	*ArsABCDH*	*P. aeruginosa*
	9	IS*Pst3*	–-	*Pseudomonas stutzeri*
	10	IS*6100*	–-	*Mycobacterium fortuitum*
	47	IS*110*-Int*I1*	*qnrVC*-*qacE*-*aadA11*	*P. aeruginosa*
	48	Tn*1213* (IS*Pa12*/IS*Pa13*)	*bla*_PER-1_-*aph(3')-VIb*	*P. aeruginosa*
DFU9	1	IS*Pa6*	*fosA*-*catB7*	*P. aeruginosa*
	2	IS*Pa11*	–-	*P. aeruginosa*
DFU16	1	IS*Pa6*	*fosA*-*catB7*	*P. aeruginosa*
	15	IS*Pa11*	–-	*P. aeruginosa*
DFU48	1	IS*Pa100*	*ArsABCDH*	*P. aeruginosa*
	9	IS*6100*	–-	*Mycobacterium fortuitum*
	30	IS*Pa32*	–-	*P. aeruginosa*
	30	IS*110*-IntI1	*qnrVC1*-*qacE*-*aadA11*	*P. aeruginosa*
	49	Tn*1213* (IS*Pa12*/IS*Pa13)*	*Bla*_PER-1_-*aph(3')-VIb*	*P. aeruginosa*
DFU58	14	IS*Pa1*	*fosA*	*P. aeruginosa*
	35	IS*Pa100*	*`ArsABCDH*	*P. aeruginosa*
	78	IS*91* family transposase	*Ant(4')-IIb*	NA
	104	IS*6100*	–-	*Mycobacterium fortuitum*

NA, The origin of the IS could not be determined as the sequence is not complete

**Fig. 4:**
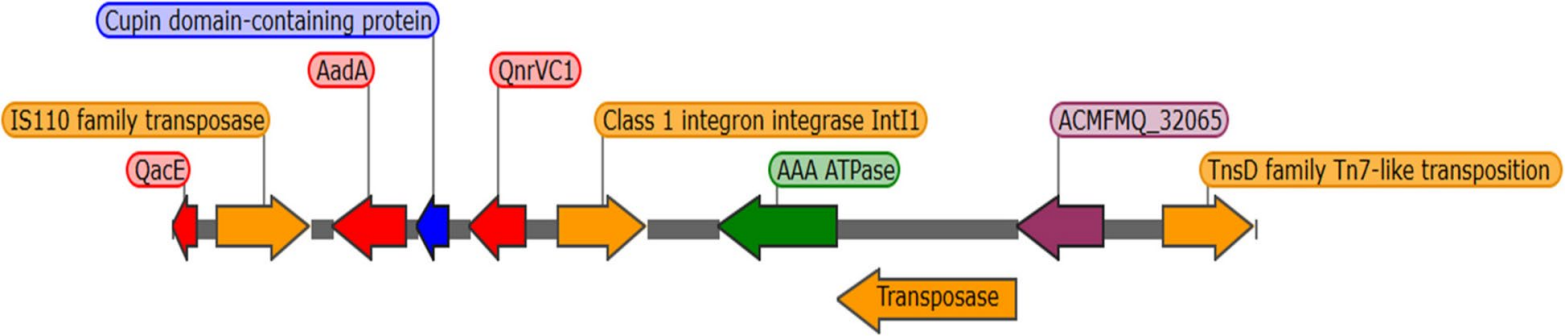
Genetic map illustrating the organization of the integron carried by DFU7 and DFU48. Arrows represent open reading frames (ORFs), with colors indicating gene function: orange (transposase and integrase genes), red (AMR genes), green (ATPase), blue (structural protein), and purple (hypothetical protein)

An integron-like region associated with IS*110* was identified in isolates DFU7 and DFU48 of length 5260 bp, showing 100% identity to *P. aeruginosa*. The element contained several *attC* recombination sites and an array of resistance cassettes, including *aadA11* (aminoglycoside 3″-adenylyltransferase), *qacE* (efflux gene) and *qnrVC1* (quinolone resistance gene). The class I integron integrase-coding gene *intI1* is located downstream of the cassette array (Fig. 4). Several MGEs shared high sequence similarity with *P. aeruginosa* strain AR_0111, *M. fortuitum* Tn*610*, and plasmid-borne integrons (AY139602), highlighting their role in resistance gene acquisition and dissemination.

**Fig. 5:**
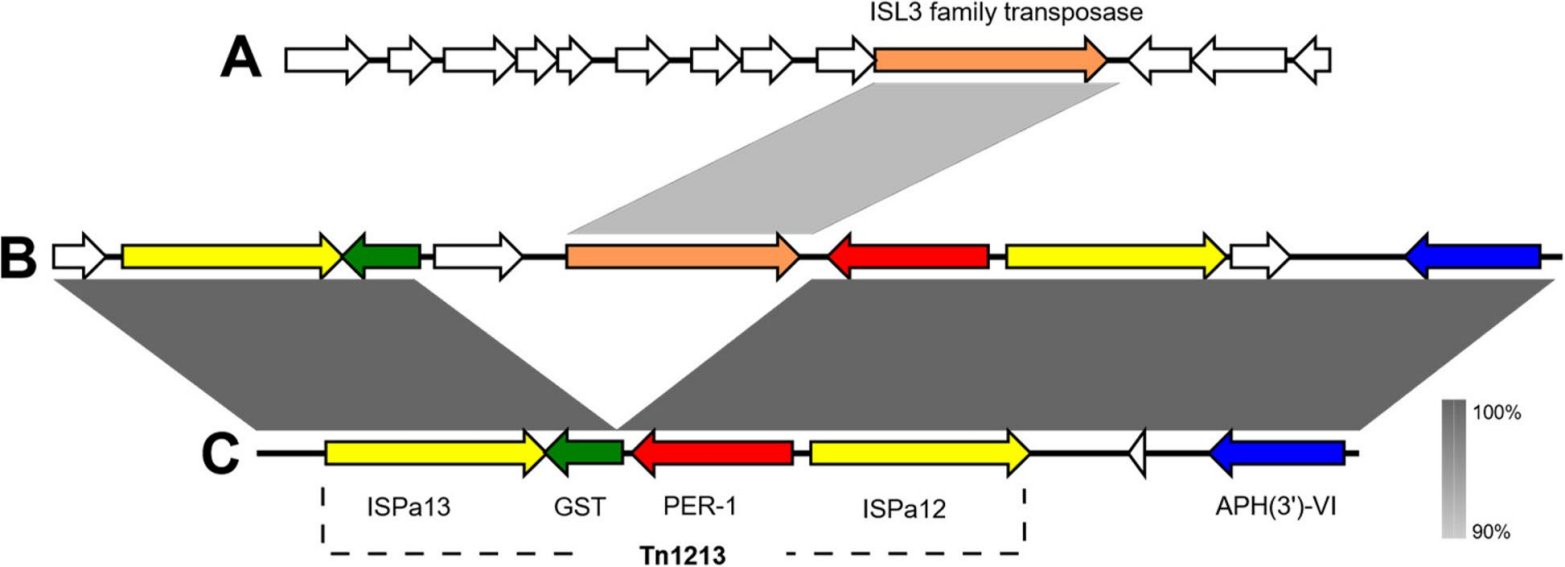
Gene maps depicting the Tn*1213* transposon-mediated *bla*_PER-1_ insertion in DFU7 (**B**), compared to *Pseudomonas* sp. 18–147 plasmid pPA18-147–2 (GenBank Accession: CP182297.1—region: 32,000–38000) (**A**) and *P. aeruginosa* isolates 3796 A chromosome (GenBank accession: OX638564 – region: 2,854,245–2,860,582) (**C**). Arrows represent ORFs and are labelled by their predicted protein products. Grey panels between maps correspond to the similarity percentage. The figure was created by Easyfig version 2.2.5

The *bla*_PER-1_gene was located within the composite transposon Tn*1213*, flanked by IS*Pa12* and IS*Pa13* (Fig. 5). However, in a novel arrangement, the transposon was disrupted by a new IS*L3* family IS carrying a cargo gene that encodes a DUF1643, domain-containing protein. Homologous ISs were exclusively identified on plasmids and at a single chromosomal location from *P. aeruginosa* isolates deposited in the NCBI database, showing a maximum coverage of 93% and a maximum identity of 93.18%. This IS, and its cargo genes interrupted a glutathione-S-transferase coding gene, generating an eight-base target site duplication (TTATTAGG) characteristic of transposition.

Analysis of the *P. aeruginosa* isolates using the ICEfinder tool identified multiple ICEs exhibiting diverse structural and functional profiles (Supplementary Table 3). The three isolates belonging to high-risk clones-DFU7, DFU48, and DFU58-each harbored two ICEs: one associated with metal resistance and another carrying anti-phage defense systems.

In DFU7 and DFU48, the metal resistance ICE contained genes conferring arsenic resistance, while the defense ICE (defense island) encoded defense systems of the Hachiman and Wadjet types. These ICEs also carried additional genes, including those encoding a putative nuclease YhcG (involved in DNA recombination and repair) and catalase, as well as genes for integrase and type IV secretion system (T4SS) proteins.

The ICEs identified in DFU58 included a defense ICE carrying three defense systems-the type IIG restriction–modification system, the AbiE defense system, and the RloC defense system-along with integrase and T4SS protein-coding genes. The second ICE in this isolate encoded copper resistance genes.

### Anti-phage defense systems and prophage regions

**Fig. 6:**
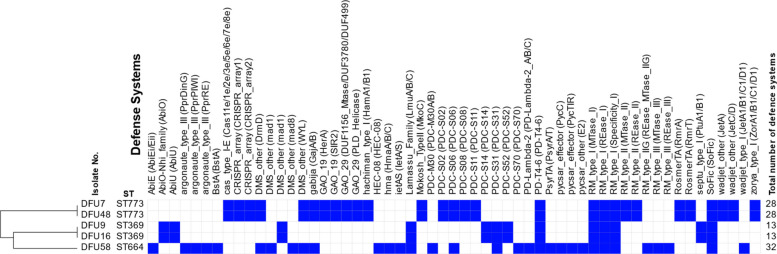
Heatmap summarizing the anti-phage defense systems identified by PADLOC in the isolates analyzed in this study. Blue squares indicate the presence of a defense system, while white squares denote its absence. The proteins associated with each defense system are shown in parentheses. Clustering was performed based on the presence or absence of these defense systems to illustrate similarities among the isolates

In addition to the defense systems detected by ICEfinder, a variety of defense systems were identified in all isolates using the PADLOC and DefenseFinder tools. The defense systems predicted by PADLOC are summarized in Fig. 6, with detailed information on their genomic locations are provided in Supplementary Table 4. The DefenseFinder results are presented in Supplementary Tables 5 and 6.

Notably, a greater diversity of defense systems was observed in isolates belonging to high-risk clones, particularly in DFU58 (ST664), DFU7, and DFU48 (ST773), compared with DFU9 and DFU16, which both belong to ST369. Interestingly, isolates sharing the same sequence type exhibited an identical number of defense systems.

Despite the relatively large number of defense systems carried by our isolates, the prophage analysis of the *P. aeruginosa* isolates revealed multiple prophage regions of varying completeness and size across the genomes. Isolates DFU7 and DFU48 each contained four intact prophage regions, while DFU9 and DFU16 harbored two intact prophages each. DFU58 exhibited the highest number of intact prophages, with six regions identified. Interestingly, intact prophage regions showing the highest similarity to *Pseudomonas* phage Pf1, previously associated with biofilm formation, were identified in DFU9 and DFU16. Details of all identified prophage regions, including their sizes, completeness, genomic locations, and the phages showing the highest number of proteins most like those in each region, are provided in Supplementary Table 7.

Surprisingly, the isolate DFU58, which carried the largest number of defense systems (*n* = 32), also harbored the greatest number of intact prophage regions (*n* = 6). In contrast, isolates DFU9 and DFU16, which possessed the lowest number of defense systems (13 each), also contained the fewest intact prophage regions (*n* = 2). DFU7 and DFU48 carried 28 defense systems and three intact prophage regions each. Furthermore, certain defense systems were found to be located within prophage regions. For example, Lamassu family antiphage defense systems were identified within an intact prophage showing the highest sequence similarity to PHAGE_Pseudo_Pf1_NC_001331 in DFU9 and DFU16, while the BstA defense system was located within an incomplete prophage region most like PHAGE_Pseudo_YMC11/02/R656_NC_028657 in DFU58.

## Discussion

DFUs represent one of the most common and severe complications of diabetes mellitus, primarily resulting from peripheral neuropathy and peripheral arterial disease [4, 93, 121]. These ulcers are frequently infected, with over 50% showing clinical signs of infection at initial evaluation, contributing to considerable morbidity and increased risk of lower-limb amputation and mortality [58, 92, 99]. Effective management of infected DFUs remains a significant clinical challenge, hindered by multiple factors including impaired immunity, poor vascularization, and delayed wound healing. The complexity is further heightened by the polymicrobial nature of these wounds [25, 118, 128], the ability of pathogens to form biofilms, and the growing prevalence of AMR among the infecting microorganisms.

*P. aeruginosa* is one of the most frequently isolated pathogens from DFUs worldwide, with a growing prevalence in both hospital and community settings in low- and middle-income countries [11, 21]. In Egypt and across North Africa, its incidence in chronic wound infections has markedly increased [41], often associated with MDR profiles that complicate treatment and elevate the risk of limb amputation [60, 130]. Several Egyptian and global studies have reported that *P. aeruginosa* ranks among the top three pathogens in infected DFUs and wound infections [3, 8, 52, 109, 112]. Its adaptability to moist environments, intrinsic resistance mechanisms, and capacity for biofilm formation contribute to its persistence in DFUs, particularly in patients with poor wound care or repeated antibiotic exposure [6, 61]. The high prevalence of MDR *P. aeruginosa* isolates in African settings may be linked to unregulated antibiotic use, limited infection control practices, and lack of routine microbiological diagnostics. In Egypt, molecular surveillance of DFU-associated isolates has revealed the frequent occurrence of ESBL and carbapenemase producers, including those harboring *bla*_NDM-1_, *bla*_PER-1_, and OXA-type β-lactamase-coding genes [6, 43].

In the current study, the isolation of five *P. aeruginosa* isolates from DFUs in diabetic patients in Egypt reflects the ongoing challenge posed by this opportunistic pathogen. The phenotypic antimicrobial susceptibility profiles of isolates DFU7 and DFU58 demonstrated a PDR phenotype, showing resistance to all tested antimicrobial agents. In contrast, isolates DFU9, DFU16, and DFU48 exhibited an MDR phenotype, all showing susceptibility to aztreonam and amikacin.

Biofilm formation is a major virulence mechanism in DFU infections, promoted by high glucose levels that enhance bacterial adhesion and extracellular polymeric substances (EPS) production. The EPS matrix protects bacteria from antibiotics and immune responses by limiting drug penetration and phagocytosis [115]. Consistent with these findings, all *P. aeruginosa* isolates in the current study demonstrated strong biofilm-forming ability as confirmed by the crystal violet assay, underscoring the role of biofilms in chronic infection and treatment resistance in DFUs.

The MLST analysis of the isolates revealed the presence of three distinct STs: ST773 (DFU7 and DFU48), ST369 (DFU9 and DFU16), and ST664 (DFU58). These STs are of growing clinical and epidemiological concern, especially in the context of chronic wound infections such as DFUs [70]. Genomic surveillance in Africa has revealed high-risk clones of *P. aeruginosa*, including ST664, which are associated with carbapenem resistance and MDR, indicating significant challenges for public health. This ST, found in DFU58, has been sporadically associated with healthcare outbreaks in Africa and the Middle East [22, 83]. ST773, detected in DFU7 and DFU48, is a globally recognized high-risk clone known for its association with *bla*_NDM-1_, *bla*_OXA-395_ and aminoglycoside-modifying enzymes, and has been increasingly reported in Egypt [2, 49, 136] Phylogenetic analysis based on WGS showed that isolates DFU7 and DFU48 (ST773) clustered with isolates from diverse geographical regions, while DFU58 (ST664) similarly grouped with international isolates, reflecting the global dissemination of these high-risk lineages.

On the other hand, DFU9 and DFU16, both assigned to ST369, showed no close matches in the BV-BRC genomic database. Only two entries with this ST were found in the PubMLST database-one from Saudi Arabia and another from an unspecified country. Although not yet a dominant global clone, ST369 has been reported more frequently in MDR settings and shows resistance traits linked to upregulated efflux systems [64]. This emerging sequence type therefore warrants close genomic and epidemiological monitoring, as its increasing association with MDR and efflux-mediated resistance mechanisms may signal the early evolution of a potentially high-risk clone that could spread more widely if not carefully tracked.

Most of the strains belonged to serotypes O6 and O11, which are among the most prevalent serotypes and are frequently associated with high-risk clones [39, 107]. Isolate DFU58 was classified as serotype O2, another commonly reported serotype [96]. Although DFU58 belonged to a high-risk clone, the O2 serotype has previously been associated with lower mortality rates compared to other serotypes [86]. This finding suggests that the O2 serotype may now be emerging within high-risk lineages, indicating a possible shift in its clinical significance and underscoring the need to reassess its role in virulence and resistance evolution.

The MDR and PDR profiles of the isolates obtained in the current study were supported by a diverse resistome potentially affecting multiple classes of antibiotics and biocides. In addition to intrinsic chromosomal resistance genes encoding efflux pumps and class D and class C β-lactamases, as well as genes conferring resistance to aminoglycosides (*aph(3')-IIb*), chloramphenicol (*catB7*), and fosfomycin (*fosA*), the high-risk clones harbored an extensive arsenal of acquired resistance determinants.

The highest number of resistance genes was identified in the ST773 isolates (DFU7 and DFU48). These isolates uniquely carried the carbapenemase gene *bla*_NDM-1_ and the extended-spectrum β-lactamase gene *bla*_PER-1_, both of which are known to mediate high-level resistance to two of the most critical antibiotic classes used to treat *P. aeruginosa* infections—carbapenems and extended-spectrum cephalosporins [72, 87, 103]. Despite the presence of β-lactamase resistance genes, some isolates remained susceptible to aztreonam. This observation may be explained by aztreonam’s relative stability against hydrolysis by several β-lactamases, allowing it to retain activity in certain β-lactamase- producing strains [17]. The presence of *bla*_NDM-1_ and *bla*_PER-1_ in *P. aeruginosa* isolates from Egypt has also been reported previously [18, 46, 134]. In addition, the ST773 strains carried several unique resistance genes, including *qnrVC1*, *sul1*, and *tet(G)*, as well as the aminoglycoside resistance genes *aph(3')-VIb*, *aac3,* and the broad-spectrum aminoglycoside resistance gene *rmtB*, which encodes a 16S rRNA methyltransferase conferring pan-aminoglycoside resistance [85]. This set of resistance genes has been previously described as a characteristic signature of ST773 isolates [62, 110]. Interestingly, despite harboring the aminoglycoside-modifying enzyme-coding genes *rmtB* and *aph(3*'*)-VIb*, DFU48 remained phenotypically sensitive to amikacin. This discrepancy could be explained by low gene expression levels or partial functional activity of the resistance determinant, as previously reported in other studies [13,114, 120].

Although the genetic context of these resistance genes could not be fully resolved due to the fragmented nature of the short-read assemblies, the genes *bla*_NDM-1_, *sul1*, *aac*(3), *rmtB4*, *floR*, and *tet(G)* have previously been reported to be located on an ICE [62]. Other genes, such as *qnrVC* and *bla*_*PER*−1_, were also associated with MGEs, specifically an integron and transposon Tn*1213* [90], respectively. DFU58 (ST664) additionally harbored the *ant(4')-IIb* gene, which confers resistance to amikacin and was found to be associated with an IS*91* family transposase.

Mutations associated with antibiotic resistance were also detected among the isolates, including substitutions in the QRDR of *gyrA* and *gyrB* genes affecting susceptibility to fluoroquinolones. While the plasmid-mediated quinolone resistance gene *qnrVC1* was identified only in the ST773 isolates (DFU7 and DFU48), DFU58 (ST664) also exhibited phenotypic resistance to fluoroquinolones. This resistance correlated with the presence of T83I and S87L mutations in *gyrA* and *parC*, respectively, in all three resistant strains. Previous studies demonstrated that a single *gyrA* mutation combined with a *parC* mutation is sufficient to confer high-level fluoroquinolone resistance in *P. aeruginosa* [59]. In contrast, the ST369 isolates (DFU9 and DFU16), which were susceptible to fluoroquinolones, lacked mutations in such genes. The *gyrA* T83I and *parC* S87L mutations detected in ST773 and ST664 isolates are frequently reported among global *P. aeruginosa* lineages, including these same STs [23, 54, 79].

Colistin remains a last-line therapeutic option against MDR *P. aeruginosa*, particularly in chronic wound infections such as DFUs [108]. Our findings align with previous studies from Egypt and the wider region, which have reported high rates of colistin resistance among *P. aeruginosa* clinical isolates. The chromosomal mutations and regulatory activation of the *arn* operon remain the principal drivers of colistin resistance in *P. aeruginosa*, in contrast to *Enterobacterales* where plasmid-mediated *mcr* genes play a major role [14, 16, 44, 73]. Mutations affecting *PmrAB* two-component regulatory system induce the *arnBCADTEF* operon and subsequently 4-amino-L-arabinose modification of lipid A, reducing the drug’s binding affinity to lipopolysaccharides. Nonetheless, most experimentally validated resistance-conferring *pmrB* changes occur in key regulatory or transmembrane regions (e.g., L17Q, P105L, T158P, L243R), not the N-terminal residues like S2P or T4A. As ST-related polymorphism of *pmrABC* operon has been previously reported [135], mutations in *pmrA*, and *pmrB* among the studied *P. aeruginosa* isolates were analyzed in the context of their STs. The L71R mutation in *pmrA* was not found in all ST773 representative strains included in our analysis and has been previously linked to colistin resistance rather than being a simple polymorphism. However, the predicted PmrB amino acid substitutions S2P, T4A, V15I, G68S, and Y345H have been detected in both susceptible and resistant isolates, suggesting they represent background lineage-specific polymorphisms rather than adaptive resistance mechanisms. Since the L71R mutation was absent in DFU7 and DFU48 despite their reduced susceptibility to colistin, alternative resistance mechanisms such as regulation of the *arn* operon and other efflux-mediated pathways may contribute and requires further future investigation [2, 135]. Notably, the L71R mutation was absent in isolates DFU7 and DFU48, which nonetheless exhibited reduced susceptibility to colistin, indicating that additional colistin resistance mechanisms remain to be elucidated.

Beyond their antibiotic resistance profiles, in silico genome analysis revealed the presence of genes associated with reduced susceptibility to disinfectants and antiseptics, including *triABC* and *qacE* (identified in DFU58). These findings suggest a potential for biocide tolerance based on genomic determinants; however, this has not been experimentally validated using phenotypic disinfectant or antiseptic susceptibility assays in this study. This dual resistance underscores a critical challenge for infection control in wound care units [24]. A study by Gadepalli et al. [51] found that *P. aeruginosa* isolates from DFUs exhibited poor susceptibility to antiseptics and biocides used in clinical settings, contributing to their chronic persistence.

The isolates collectively harbored minimum 230 virulence genes, encompassing adhesion, secretion, biofilm formation, toxin production, and metal acquisition. Across all STs, conservation of Type IV pili, quorum-sensing systems, alginate regulation, and siderophore biosynthesis indicates strong adaptation to chronic wound niches. Consistent with the observed strong biofilm-forming phenotype, all isolates carried genes encoding pili required for the initial attachment phase of biofilm development, as well as genes responsible for exopolysaccharide production. These findings underscore the dual challenge of MDR spread and persistence of virulent strains in vulnerable diabetic patients. However, ST-specific variations were evident. The ST773 strains uniquely harbored both the *pldA* gene, encoding phospholipase D (PLD), and the *exoU* gene, a potent cytotoxin secreted via the type III secretion system (TTSS). PLD produces phosphatidic acid, a signaling molecule implicated in inflammation and tumorigenesis [133], while ExoU is essential for the rapid killing of mammalian cells [113]. The coexistence of these two virulence determinants may substantially enhance the pathogenic potential of ST773 isolates, amplify tissue damage, inflammation, and immune evasion in diabetic foot infections, thereby contributing to more severe and difficult-to-treat ulcers in affected patients. In contrast, *exoS*, encoding another TTSS-secreted effector, was uniquely detected in ST369 and ST664. Notably, *exoU* and *exoS* have been widely used for *P. aeruginosa* virulotyping. Although both contribute to bacterial propagation and pathogenesis, they are not co-expressed within the same strain,*exoS* is primarily linked to endocytosis and intracellular persistence, whereas *exoU* triggers rapid lysis of host cell membranes [102]. Variability in T3SS effectors (*exoS*, *exoU*) further reflects clonal diversity, as documented in global *P. aeruginosa* populations [50, 101]. Additionally, ST773 uniquely carried *galE*, which may enhance serum resistance and immune evasion.

Comprehensive genomic screening revealed the presence of diverse MGEs, including ISs, transposons, and integron-like regions, that collectively contribute to AMR dissemination. An integron-like region linked to IS*Pa62* in DFU7 and DFU48 carried multiple AMR cassettes (*aadA11*, *qacE*, and *qnrVC1*) flanked by a XerC-like integrase, supporting its function as a class 1 integron variant, a structure frequently associated with MDR in *P. aeruginosa* [53, 91]. The detection of *bla*_PER-1_ within the composite transposon Tn*1213*, flanked by IS*Pa12* and IS*Pa13*, and disrupted by a novel IS*L3* family IS, represents a previously unreported arrangement. This element, carrying a DUF1643 domain–containing gene, exhibited only partial sequence identity to known IS*L3* members. The observed target site duplication (TTATTAGG) and flanking *IS* elements indicate active transposition and potential for mobilization across plasmid and chromosomal backgrounds, similar to recombination-mediated transposon reshuffling events reported in other *P. aeruginosa* lineages [111, 132]. Together, these findings highlight the genomic plasticity of *P. aeruginosa* and its capacity to recruit resistance determinants from diverse Gram-negative species through MGE-driven HGT [76].

Genomic analysis of our isolates revealed four distinct ICEs carried by the high-risk clones, including metal-resistance and defense islands. These elements harbored genes encoding T4SS and type IV coupling protein (T4CP), indicating active conjugation potential and suggesting their ability to mediate DNA transfer between bacterial species. Such mobile elements are major drivers of HGT and play a crucial role in the adaptation and persistence of *P. aeruginosa* in hostile environments [127]. Studying the CRISPR–Cas system is essential to clarify its influence on AMR. In addition to protecting bacteria from foreign DNA, CRISPR–Cas can affect the acquisition and persistence of mobile genetic elements, such as plasmids and integrons, that frequently carry resistance genes. Depending on its presence and type, this system may either restrict horizontal gene transfer or coexist with resistance determinants, thereby shaping the resistome of *P. aeruginosa*. Analysis of CRISPR–Cas structure and spacer sequences can therefore shed light on the evolution of AMR and its impact on genomic adaptability and treatment outcomes [122].

The detection of metal resistance ICEs emphasizes the selective pressure imposed by heavy metals, which can co-select for AMR genes due to shared regulatory pathways or physical linkage within the same MGE [63, 106].

Of particular significance is the identification of at least two defense-associated ICEs in ST773 (DFU7 and DFU48) and ST664 (DFU58). The bacterial innate defense arsenal protects against phage infection through abortive infection mechanisms and degradation of invading nucleic acids [67, 75]. It has been shown that defense systems play a key role in determining phage susceptibility in *P. aeruginosa*, with overall phage resistance increasing proportionally to the number of defense systems encoded in the genome [36]. This shows that the isolates studied here can survive not only under antimicrobial stress but also under phage attack. One of the defense systems encoded within the defense ICE of ST773 is the Hachiman system. Hachiman consists of the HamA and HamB proteins, which protect bacteria against a broad range of phages. This system can be activated not only by phage infection but also by DNA damage, indicating a role in monitoring genome integrity. When excessive DNA damage is detected, ATP-bound HamAB activates the nuclease activity of HamA, amplifying the immune response and restricting invading phages [129].

It is worth mentioning that the number of defense systems identified in the high-risk clones analyzed in this study using PADLOC exceeded those reported by Costa et al. [36], who examined 167 *P. aeruginosa* RefSeq genomes and found that only 71% carried defense systems, with a maximum of 19 and an average of 7 per genome. ST773 carried 28 defense systems, while ST664 carried 32. Despite the presence of numerous defense systems, multiple prophage regions were identified within the studied isolates. Some of these prophage regions carried defense systems, likely providing protection against superinfection by other bacteriophages [117]. One of the prophages carried by ST369 isolates and DFU48, showed the highest sequence similarity to PHAGE_Pseudo_Pf1_NC_001331. Pf1 prophage has been previously shown to contribute to *P. aeruginosa* pathogenesis through multiple mechanisms, including organizing the polymer-rich biofilm matrix into crystalline, higher-order structures that increase viscosity, adhesiveness, and resistance to desiccation. Such effects enhance antibiotic tolerance and immune evasion of bacterial cells within biofilms, which may promote persistence in wound environments such as diabetic foot ulcers [119]. One limitation of our study is the small number of isolates and the limited clinical information available, which makes it difficult to generalize our findings or directly link genomic features to patient outcomes. Another limitation of our study is that, although genomic analyses identified multiple predicted anti-phage defense systems in the recovered *P. aeruginosa* isolates, no experimental phage susceptibility or phage-challenge assays were performed to functionally validate their impact. As a result, the inferred role of these systems in conferring resistance to bacteriophages remains based solely on genomic prediction. Future work incorporating phenotypic phage infection assays will be essential to confirm their functional relevance and to better assess their implications for phage therapy applications.

## Conclusion

This study provides an integrated phenotypic and genomic characterization of *P. aeruginosa* isolates recovered from DFUs in Egypt, revealing the circulation of high-risk clones, particularly ST773 and ST664, with extensive antimicrobial resistance and virulence potential. ST773 isolates harbored the highest resistance gene burden, including clinically significant carbapenemase (*bla*_NDM-1_) and ESBL (*bla*_PER-1_) genes, alongside multiple aminoglycoside, fluoroquinolone, and tetracycline resistance determinants, many of which were embedded within MGEs. In addition, strong biofilm formation and the presence of biocide resistance genes indicate enhanced persistence of the isolates under routine wound care and infection control practices. The virulence profile was extensive and sequence-type specific, with ST773 uniquely harboring *exoU* and *pldA*, key cytotoxicity genes. The abundance of MGEs and anti-phage defense systems highlights the high genomic plasticity and adaptability of these high-risk clones, with potential implications for both persistence and resistance to phage-based therapies. Collectively, these findings highlight the convergence of MDR, virulence, environmental persistence, and phage defense in DFU-associated *P. aeruginosa* in Egypt. This work underscores the need for strengthened infection control practices and expanded genomic surveillance to mitigate the clinical impact of this highly adaptable and high-risk pathogen.

## Supplementary Information

Supplementary Material 1.

Supplementary Material 2.

## Data Availability

The complete sequence of the 16S rRNA gene from *P. aeruginosa* isolates has been submitted to the NCBI GenBank database with the following accession numbers: PV082531 (DFU7), PV083167 (DFU9), PV083168 (DFU16), PV082535 (DFU48), and PV082532 (DFU58). The draft genome assemblies are available in the NCBI BioProject PRJNA1181229 under the accession numbers: JBLFEY000000000 (DFU7), JBLFEZ000000000 (DFU9), JBLFFA000000000 (DFU16), JBLFFB000000000 (DFU48), and JBLFFC000000000 (DFU58).

## Abbreviations

AMR: Antimicrobial resistance
DFU: Diabetic foot ulcer
DTR: Difficult-to-treat resistance
GI: Genomic island
ICE: Integrative and conjugative element
IS: Insertion sequence
MDR: Multidrug-resistant
MFS: Major Facilitator Superfamily
MGEs: Mobile genetic elements
MLST: Multilocus sequence typing
NIDE: National Institute of Diabetes and Endocrinology
OSA: O-specific antigen
PDR: Pan-drug-resistant
RND: Resistance-Nodulation-Division
ST: Sequence type
TYGS: Type strain genome server
TTSS: Type III secretion system
WGS: Whole genome sequencing
XDR: Extensively drug-resistant
