# Supplementary material for: WGS reveals high-risk clones of Pseudomonas aeruginosa harbouring extensive antimicrobial and predicted anti-phage defense systems recovered from diabetic foot ulcer patients in Egypt
Source: BMC Microbiol. 2026 Jun 3;26:524. doi: 10.1186/s12866-026-05145-x (PMC13231603; doi:10.1186/s12866-026-05145-x)
Supplement: Supplementary file 1 — Supplementary Material 1. [file 12866_2026_5145_MOESM1_ESM.docx]

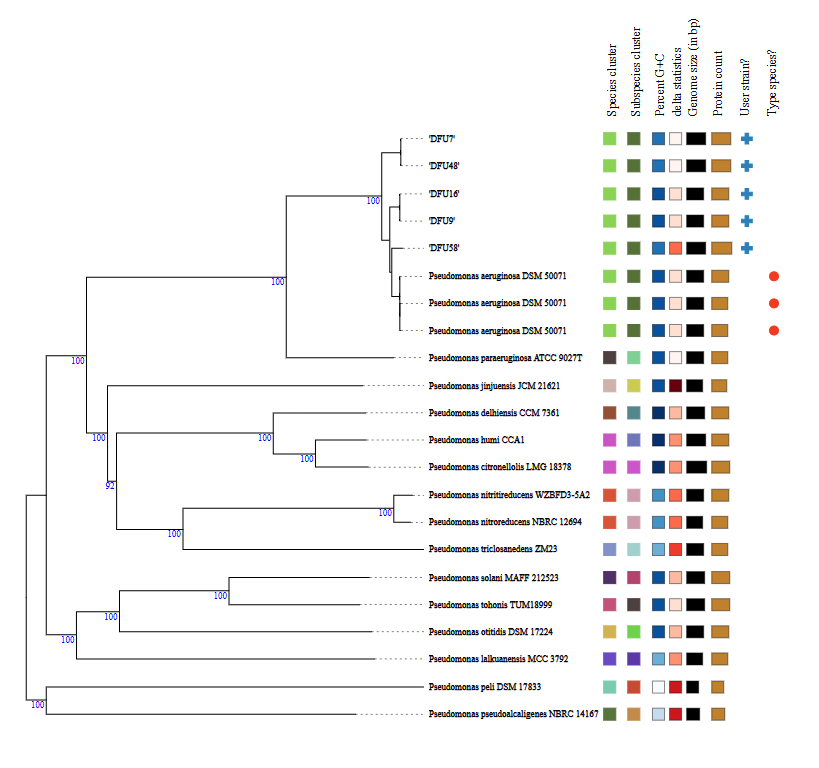


**Supplementary Figure 1: Phylogenomic tree constructed using GBDP distances derived from the genomes of TYGS type strains most closely related to *P. aeruginosa* isolates.** Clusters representing species and subspecies are defined by dDDH thresholds of 70% and 79%, respectively. Strains from the current study are highlighted in bold red within the tree.

cov pid  **1** **[ . . . . : . . .** **80**

1 DFU7 100.0% 100.0% **MGELAKEILPVNIEDELKQSYLDYAMSVIVGRALPDARDGLKPVHRRVLYAMSELGNDWNKPYKKSARVVGDVIGKYHPH**

2 DFU9 100.0% 99.9% **MGELAKEILPVNIEDELKQSYLDYAMSVIVGRALPDARDGLKPVHRRVLYAMSELGNDWNKPYKKSARVVGDVIGKYHPH**

3 DFU16 100.0% 99.9% **MGELAKEILPVNIEDELKQSYLDYAMSVIVGRALPDARDGLKPVHRRVLYAMSELGNDWNKPYKKSARVVGDVIGKYHPH**

4 DFU48 100.0% 100.0% **MGELAKEILPVNIEDELKQSYLDYAMSVIVGRALPDARDGLKPVHRRVLYAMSELGNDWNKPYKKSARVVGDVIGKYHPH**

5 DFU58 100.0% 100.0% **MGELAKEILPVNIEDELKQSYLDYAMSVIVGRALPDARDGLKPVHRRVLYAMSELGNDWNKPYKKSARVVGDVIGKYHPH**

6 PAO1 100.0% 99.9% **MGELAKEILPVNIEDELKQSYLDYAMSVIVGRALPDARDGLKPVHRRVLYAMSELGNDWNKPYKKSARVVGDVIGKYHPH**

7 ATCC33363 100.0% 99.9% **MGELAKEILPVNIEDELKQSYLDYAMSVIVGRALPDARDGLKPVHRRVLYAMSELGNDWNKPYKKSARVVGDVIGKYHPH**

8 ZBX-P25 100.0% 99.9% **MGELAKEILPVNIEDELKQSYLDYAMSVIVGRALPDARDGLKPVHRRVLYAMSELGNDWNKPYKKSARVVGDVIGKYHPH**

9 DMC-20C 100.0% 100.0% **MGELAKEILPVNIEDELKQSYLDYAMSVIVGRALPDARDGLKPVHRRVLYAMSELGNDWNKPYKKSARVVGDVIGKYHPH**

10 P8W 100.0% 100.0% **MGELAKEILPVNIEDELKQSYLDYAMSVIVGRALPDARDGLKPVHRRVLYAMSELGNDWNKPYKKSARVVGDVIGKYHPH**

11 UTDF19-28A 100.0% 99.9% **MGELAKEILPVNIEDELKQSYLDYAMSVIVGRALPDARDGLKPVHRRVLYAMSELGNDWNKPYKKSARVVGDVIGKYHPH**

12 CMPL223 99.8% 99.2% **MGELAKEILPVNIEDELKQSYLDYAMSVIVGRALPDARDGLKPVHRRVLYAMSELGNDWNKPYKKSARVVGDVIGKYHPH**

13 AS_23 100.0% 100.0% **MGELAKEILPVNIEDELKQSYLDYAMSVIVGRALPDARDGLKPVHRRVLYAMSELGNDWNKPYKKSARVVGDVIGKYHPH**

14 PS1972 100.0% 99.9% **MGELAKEILPVNIEDELKQSYLDYAMSVIVGRALPDARDGLKPVHRRVLYAMSELGNDWNKPYKKSARVVGDVIGKYHPH**

15 BA10747 100.0% 100.0% **MGELAKEILPVNIEDELKQSYLDYAMSVIVGRALPDARDGLKPVHRRVLYAMSELGNDWNKPYKKSARVVGDVIGKYHPH**

16 ST773 100.0% 100.0% **MGELAKEILPVNIEDELKQSYLDYAMSVIVGRALPDARDGLKPVHRRVLYAMSELGNDWNKPYKKSARVVGDVIGKYHPH**

17 DSM50071 100.0% 99.9% **MGELAKEILPVNIEDELKQSYLDYAMSVIVGRALPDARDGLKPVHRRVLYAMSELGNDWNKPYKKSARVVGDVIGKYHPH**

consensus/100% **MGELAKEILPVNIEDELKQSYLDYAMSVIVGRALPDARDGLKPVHRRVLYAMSELGNDWNKPYKKSARVVGDVIGKYHPH**

consensus/90% **MGELAKEILPVNIEDELKQSYLDYAMSVIVGRALPDARDGLKPVHRRVLYAMSELGNDWNKPYKKSARVVGDVIGKYHPH**

consensus/80% **MGELAKEILPVNIEDELKQSYLDYAMSVIVGRALPDARDGLKPVHRRVLYAMSELGNDWNKPYKKSARVVGDVIGKYHPH**

consensus/70% **MGELAKEILPVNIEDELKQSYLDYAMSVIVGRALPDARDGLKPVHRRVLYAMSELGNDWNKPYKKSARVVGDVIGKYHPH**

cov pid  **81**  **. 1 . . . . : .** **160**

1 DFU7 100.0% 100.0% **GDIAVYDTIVRMAQPFSLRYMLVDGQGNFGSVDGDNAAAMRYTEVRMAKLAHELLADLEKETVDWVPNYDGTEQIPAVMP**

2 DFU9 100.0% 99.9% **GDTAVYDTIVRMAQPFSLRYMLVDGQGNFGSVDGDNAAAMRYTEVRMAKLAHELLADLEKETVDWVPNYDGTEQIPAVMP**

3 DFU16 100.0% 99.9% **GDTAVYDTIVRMAQPFSLRYMLVDGQGNFGSVDGDNAAAMRYTEVRMAKLAHELLADLEKETVDWVPNYDGTEQIPAVMP**

4 DFU48 100.0% 100.0% **GDIAVYDTIVRMAQPFSLRYMLVDGQGNFGSVDGDNAAAMRYTEVRMAKLAHELLADLEKETVDWVPNYDGTEQIPAVMP**

5 DFU58 100.0% 100.0% **GDIAVYDTIVRMAQPFSLRYMLVDGQGNFGSVDGDNAAAMRYTEVRMAKLAHELLADLEKETVDWVPNYDGTEQIPAVMP**

6 PAO1 100.0% 99.9% **GDTAVYDTIVRMAQPFSLRYMLVDGQGNFGSVDGDNAAAMRYTEVRMAKLAHELLADLEKETVDWVPNYDGTEQIPAVMP**

7 ATCC33363 100.0% 99.9% **GDTAVYDTIVRMAQPFSLRYMLVDGQGNFGSVDGDNAAAMRYTEVRMAKLAHELLADLEKETVDWVPNYDGTEQIPAVMP**

8 ZBX-P25 100.0% 99.9% **GDIAVYNTIVRMAQPFSLRYMLVDGQGNFGSVDGDNAAAMRYTEVRMAKLAHELLADLEKETVDWVPNYDGTEQIPAVMP**

9 DMC-20C 100.0% 100.0% **GDIAVYDTIVRMAQPFSLRYMLVDGQGNFGSVDGDNAAAMRYTEVRMAKLAHELLADLEKETVDWVPNYDGTEQIPAVMP**

10 P8W 100.0% 100.0% **GDIAVYDTIVRMAQPFSLRYMLVDGQGNFGSVDGDNAAAMRYTEVRMAKLAHELLADLEKETVDWVPNYDGTEQIPAVMP**

11 UTDF19-28A 100.0% 99.9% **GDTAVYDTIVRMAQPFSLRYMLVDGQGNFGSVDGDNAAAMRYTEVRMAKLAHELLADLEKETVDWVPNYDGTEQIPAVMP**

12 CMPL223 99.8% 99.2% **GDIAVYDTIVRMAQPFSLRYMLVDGQGNFGSVDGDNAAAMRYTEVRMAKLAHELLADLEKETVDWVPNYDGTEQIPAVMP**

13 AS_23 100.0% 100.0% **GDIAVYDTIVRMAQPFSLRYMLVDGQGNFGSVDGDNAAAMRYTEVRMAKLAHELLADLEKETVDWVPNYDGTEQIPAVMP**

14 PS1972 100.0% 99.9% **GDTAVYDTIVRMAQPFSLRYMLVDGQGNFGSVDGDNAAAMRYTEVRMAKLAHELLADLEKETVDWVPNYDGTEQIPAVMP**

15 BA10747 100.0% 100.0% **GDIAVYDTIVRMAQPFSLRYMLVDGQGNFGSVDGDNAAAMRYTEVRMAKLAHELLADLEKETVDWVPNYDGTEQIPAVMP**

16 ST773 100.0% 100.0% **GDIAVYDTIVRMAQPFSLRYMLVDGQGNFGSVDGDNAAAMRYTEVRMAKLAHELLADLEKETVDWVPNYDGTEQIPAVMP**

17 DSM50071 100.0% 99.9% **GDTAVYDTIVRMAQPFSLRYMLVDGQGNFGSVDGDNAAAMRYTEVRMAKLAHELLADLEKETVDWVPNYDGTEQIPAVMP**

consensus/100% **GDhAVYsTIVRMAQPFSLRYMLVDGQGNFGSVDGDNAAAMRYTEVRMAKLAHELLADLEKETVDWVPNYDGTEQIPAVMP**

consensus/90% **GDhAVYDTIVRMAQPFSLRYMLVDGQGNFGSVDGDNAAAMRYTEVRMAKLAHELLADLEKETVDWVPNYDGTEQIPAVMP**

consensus/80% **GDhAVYDTIVRMAQPFSLRYMLVDGQGNFGSVDGDNAAAMRYTEVRMAKLAHELLADLEKETVDWVPNYDGTEQIPAVMP**

consensus/70% **GDhAVYDTIVRMAQPFSLRYMLVDGQGNFGSVDGDNAAAMRYTEVRMAKLAHELLADLEKETVDWVPNYDGTEQIPAVMP**

cov pid **161**  **. . . 2 . . . .** **240**

1 DFU7 100.0% 100.0% **TKIPNLLVNGSSGIAVGMATNIPPHNLGEVIDGCLALMDNPDLTVDELMQYIPGPDFPTAGIINGRAGIIEAYRTGRGRI**

2 DFU9 100.0% 99.9% **TKIPNLLVNGSSGIAVGMATNIPPHNLGEVIDGCLALMDNPDLTVDELMQYIPGPDFPTAGIINGRAGIIEAYRTGRGRI**

3 DFU16 100.0% 99.9% **TKIPNLLVNGSSGIAVGMATNIPPHNLGEVIDGCLALMDNPDLTVDELMQYIPGPDFPTAGIINGRAGIIEAYRTGRGRI**

4 DFU48 100.0% 100.0% **TKIPNLLVNGSSGIAVGMATNIPPHNLGEVIDGCLALMDNPDLTVDELMQYIPGPDFPTAGIINGRAGIIEAYRTGRGRI**

5 DFU58 100.0% 100.0% **TKIPNLLVNGSSGIAVGMATNIPPHNLGEVIDGCLALMDNPDLTVDELMQYIPGPDFPTAGIINGRAGIIEAYRTGRGRI**

6 PAO1 100.0% 99.9% **TKIPNLLVNGSSGIAVGMATNIPPHNLGEVIDGCLALMDNPDLTVDELMQYIPGPDFPTAGIINGRAGIIEAYRTGRGRI**

7 ATCC33363 100.0% 99.9% **TKIPNLLVNGSSGIAVGMATNIPPHNLGEVIDGCLALMDNPDLTVDELMQYIPGPDFPTAGIINGRAGIIEAYRTGRGRI**

8 ZBX-P25 100.0% 99.9% **TKIPNLLVNGSSGIAVGMATNIPPHNLGEVIDGCLALMDNPDLTVDELMQYIPGPDFPTAGIINGRAGIIEAYRTGRGRI**

9 DMC-20C 100.0% 100.0% **TKIPNLLVNGSSGIAVGMATNIPPHNLGEVIDGCLALMDNPDLTVDELMQYIPGPDFPTAGIINGRAGIIEAYRTGRGRI**

10 P8W 100.0% 100.0% **TKIPNLLVNGSSGIAVGMATNIPPHNLGEVIDGCLALMDNPDLTVDELMQYIPGPDFPTAGIINGRAGIIEAYRTGRGRI**

11 UTDF19-28A 100.0% 99.9% **TKIPNLLVNGSSGIAVGMATNIPPHNLGEVIDGCLALMDNPDLTVDELMQYIPGPDFPTAGIINGRAGIIEAYRTGRGRI**

12 CMPL223 99.8% 99.2% **TKIPNLLVNGSSGIAVGMATNIPPHNLGEVIDGCLALMDNPDLTVDELMQYIPGPDFPTAGIINGRAGIIEAYRTGRGRI**

13 AS_23 100.0% 100.0% **TKIPNLLVNGSSGIAVGMATNIPPHNLGEVIDGCLALMDNPDLTVDELMQYIPGPDFPTAGIINGRAGIIEAYRTGRGRI**

14 PS1972 100.0% 99.9% **TKIPNLLVNGSSGIAVGMATNIPPHNLGEVIDGCLALMDNPDLTVDELMQYIPGPDFPTAGIINGRAGIIEAYRTGRGRI**

15 BA10747 100.0% 100.0% **TKIPNLLVNGSSGIAVGMATNIPPHNLGEVIDGCLALMDNPDLTVDELMQYIPGPDFPTAGIINGRAGIIEAYRTGRGRI**

16 ST773 100.0% 100.0% **TKIPNLLVNGSSGIAVGMATNIPPHNLGEVIDGCLALMDNPDLTVDELMQYIPGPDFPTAGIINGRAGIIEAYRTGRGRI**

17 DSM50071 100.0% 99.9% **TKIPNLLVNGSSGIAVGMATNIPPHNLGEVIDGCLALMDNPDLTVDELMQYIPGPDFPTAGIINGRAGIIEAYRTGRGRI**

consensus/100% **TKIPNLLVNGSSGIAVGMATNIPPHNLGEVIDGCLALMDNPDLTVDELMQYIPGPDFPTAGIINGRAGIIEAYRTGRGRI**

consensus/90% **TKIPNLLVNGSSGIAVGMATNIPPHNLGEVIDGCLALMDNPDLTVDELMQYIPGPDFPTAGIINGRAGIIEAYRTGRGRI**

consensus/80% **TKIPNLLVNGSSGIAVGMATNIPPHNLGEVIDGCLALMDNPDLTVDELMQYIPGPDFPTAGIINGRAGIIEAYRTGRGRI**

consensus/70% **TKIPNLLVNGSSGIAVGMATNIPPHNLGEVIDGCLALMDNPDLTVDELMQYIPGPDFPTAGIINGRAGIIEAYRTGRGRI**

cov pid **241**  **: . . . . 3 . .** **320**

1 DFU7 100.0% 100.0% **YIRARAVVEEMEKGGGREQIIITELPYQLNKARLIEKIAELVKEKKIEGISELRDESDKDGMRVVIELRRGEVGEVVLNN**

2 DFU9 100.0% 99.9% **YIRARAVVEEMEKGGGREQIIITELPYQLNKARLIEKIAELVKEKKIEGISELRDESDKDGMRVVIELRRGEVGEVVLNN**

3 DFU16 100.0% 99.9% **YIRARAVVEEMEKGGGREQIIITELPYQLNKARLIEKIAELVKEKKIEGISELRDESDKDGMRVVIELRRGEVGEVVLNN**

4 DFU48 100.0% 100.0% **YIRARAVVEEMEKGGGREQIIITELPYQLNKARLIEKIAELVKEKKIEGISELRDESDKDGMRVVIELRRGEVGEVVLNN**

5 DFU58 100.0% 100.0% **YIRARAVVEEMEKGGGREQIIITELPYQLNKARLIEKIAELVKEKKIEGISELRDESDKDGMRVVIELRRGEVGEVVLNN**

6 PAO1 100.0% 99.9% **YIRARAVVEEMEKGGGREQIIITELPYQLNKARLIEKIAELVKEKKIEGISELRDESDKDGMRVVIELRRGEVGEVVLNN**

7 ATCC33363 100.0% 99.9% **YIRARAVVEEMEKGGGREQIIITELPYQLNKARLIEKIAELVKEKKIEGISELRDESDKDGMRVVIELRRGEVGEVVLNN**

8 ZBX-P25 100.0% 99.9% **YIRARAVVEEMEKGGGREQIIITELPYQLNKARLIEKIAELVKEKKIEGISELRDESDKDGMRVVIELRRGEVGEVVLNN**

9 DMC-20C 100.0% 100.0% **YIRARAVVEEMEKGGGREQIIITELPYQLNKARLIEKIAELVKEKKIEGISELRDESDKDGMRVVIELRRGEVGEVVLNN**

10 P8W 100.0% 100.0% **YIRARAVVEEMEKGGGREQIIITELPYQLNKARLIEKIAELVKEKKIEGISELRDESDKDGMRVVIELRRGEVGEVVLNN**

11 UTDF19-28A 100.0% 99.9% **YIRARAVVEEMEKGGGREQIIITELPYQLNKARLIEKIAELVKEKKIEGISELRDESDKDGMRVVIELRRGEVGEVVLNN**

12 CMPL223 99.8% 99.2% **YIRARAVVEEMEKGGGREQIIITELPYQLNKARLIEKIAELVKEKKIEGISELRDESDKDGMRVVIELRRGEVGEVVLNN**

13 AS_23 100.0% 100.0% **YIRARAVVEEMEKGGGREQIIITELPYQLNKARLIEKIAELVKEKKIEGISELRDESDKDGMRVVIELRRGEVGEVVLNN**

14 PS1972 100.0% 99.9% **YIRARAVVEEMEKGGGREQIIITELPYQLNKARLIEKIAELVKEKKIEGISELRDESDKDGMRVVIELRRGEVGEVVLNN**

15 BA10747 100.0% 100.0% **YIRARAVVEEMEKGGGREQIIITELPYQLNKARLIEKIAELVKEKKIEGISELRDESDKDGMRVVIELRRGEVGEVVLNN**

16 ST773 100.0% 100.0% **YIRARAVVEEMEKGGGREQIIITELPYQLNKARLIEKIAELVKEKKIEGISELRDESDKDGMRVVIELRRGEVGEVVLNN**

17 DSM50071 100.0% 99.9% **YIRARAVVEEMEKGGGREQIIITELPYQLNKARLIEKIAELVKEKKIEGISELRDESDKDGMRVVIELRRGEVGEVVLNN**

consensus/100% **YIRARAVVEEMEKGGGREQIIITELPYQLNKARLIEKIAELVKEKKIEGISELRDESDKDGMRVVIELRRGEVGEVVLNN**

consensus/90% **YIRARAVVEEMEKGGGREQIIITELPYQLNKARLIEKIAELVKEKKIEGISELRDESDKDGMRVVIELRRGEVGEVVLNN**

consensus/80% **YIRARAVVEEMEKGGGREQIIITELPYQLNKARLIEKIAELVKEKKIEGISELRDESDKDGMRVVIELRRGEVGEVVLNN**

consensus/70% **YIRARAVVEEMEKGGGREQIIITELPYQLNKARLIEKIAELVKEKKIEGISELRDESDKDGMRVVIELRRGEVGEVVLNN**

cov pid **321**  **. . : . . . . 4** **400**

1 DFU7 100.0% 100.0% **LYAQTQLQSVFGINVVALVDGQPRTLNLKDMLEVFVRHRREVVTRRTVYELRKARERGHILEGQAVALSNIDPVIELIKS**

2 DFU9 100.0% 99.9% **LYAQTQLQSVFGINVVALVDGQPRTLNLKDMLEVFVRHRREVVTRRTVYELRKARERGHILEGQAVALSNIDPVIELIKS**

3 DFU16 100.0% 99.9% **LYAQTQLQSVFGINVVALVDGQPRTLNLKDMLEVFVRHRREVVTRRTVYELRKARERGHILEGQAVALSNIDPVIELIKS**

4 DFU48 100.0% 100.0% **LYAQTQLQSVFGINVVALVDGQPRTLNLKDMLEVFVRHRREVVTRRTVYELRKARERGHILEGQAVALSNIDPVIELIKS**

5 DFU58 100.0% 100.0% **LYAQTQLQSVFGINVVALVDGQPRTLNLKDMLEVFVRHRREVVTRRTVYELRKARERGHILEGQAVALSNIDPVIELIKS**

6 PAO1 100.0% 99.9% **LYAQTQLQSVFGINVVALVDGQPRTLNLKDMLEVFVRHRREVVTRRTVYELRKARERGHILEGQAVALSNIDPVIELIKS**

7 ATCC33363 100.0% 99.9% **LYAQTQLQSVFGINVVALVDGQPRTLNLKDMLEVFVRHRREVVTRRTVYELRKARERGHILEGQAVALSNIDPVIELIKS**

8 ZBX-P25 100.0% 99.9% **LYAQTQLQSVFGINVVALVDGQPRTLNLKDMLEVFVRHRREVVTRRTVYELRKARERGHILEGQAVALSNIDPVIELIKS**

9 DMC-20C 100.0% 100.0% **LYAQTQLQSVFGINVVALVDGQPRTLNLKDMLEVFVRHRREVVTRRTVYELRKARERGHILEGQAVALSNIDPVIELIKS**

10 P8W 100.0% 100.0% **LYAQTQLQSVFGINVVALVDGQPRTLNLKDMLEVFVRHRREVVTRRTVYELRKARERGHILEGQAVALSNIDPVIELIKS**

11 UTDF19-28A 100.0% 99.9% **LYAQTQLQSVFGINVVALVDGQPRTLNLKDMLEVFVRHRREVVTRRTVYELRKARERGHILEGQAVALSNIDPVIELIKS**

12 CMPL223 99.8% 99.2% **LYAQTQLQSVFGINVVALVDGQPRTLNLKDMLEVFVRHRREVVTRRTVYELRKARERGHILEGQAVALSNIDPVIELIKS**

13 AS_23 100.0% 100.0% **LYAQTQLQSVFGINVVALVDGQPRTLNLKDMLEVFVRHRREVVTRRTVYELRKARERGHILEGQAVALSNIDPVIELIKS**

14 PS1972 100.0% 99.9% **LYAQTQLQSVFGINVVALVDGQPRTLNLKDMLEVFVRHRREVVTRRTVYELRKARERGHILEGQAVALSNIDPVIELIKS**

15 BA10747 100.0% 100.0% **LYAQTQLQSVFGINVVALVDGQPRTLNLKDMLEVFVRHRREVVTRRTVYELRKARERGHILEGQAVALSNIDPVIELIKS**

16 ST773 100.0% 100.0% **LYAQTQLQSVFGINVVALVDGQPRTLNLKDMLEVFVRHRREVVTRRTVYELRKARERGHILEGQAVALSNIDPVIELIKS**

17 DSM50071 100.0% 99.9% **LYAQTQLQSVFGINVVALVDGQPRTLNLKDMLEVFVRHRREVVTRRTVYELRKARERGHILEGQAVALSNIDPVIELIKS**

consensus/100% **LYAQTQLQSVFGINVVALVDGQPRTLNLKDMLEVFVRHRREVVTRRTVYELRKARERGHILEGQAVALSNIDPVIELIKS**

consensus/90% **LYAQTQLQSVFGINVVALVDGQPRTLNLKDMLEVFVRHRREVVTRRTVYELRKARERGHILEGQAVALSNIDPVIELIKS**

consensus/80% **LYAQTQLQSVFGINVVALVDGQPRTLNLKDMLEVFVRHRREVVTRRTVYELRKARERGHILEGQAVALSNIDPVIELIKS**

consensus/70% **LYAQTQLQSVFGINVVALVDGQPRTLNLKDMLEVFVRHRREVVTRRTVYELRKARERGHILEGQAVALSNIDPVIELIKS**

cov pid **401**  **. . . . : . . .** **480**

1 DFU7 100.0% 100.0% **SPTPAEAKERLIATAWESSAVEAMVERAGADACRPEDLDPQYGLRDGKYYLSPEQAQAILELRLHRLTGLEHEKLLSEYQ**

2 DFU9 100.0% 99.9% **SPTPAEAKERLIATAWESSAVEAMVERAGADACRPEDLDPQYGLRDGKYYLSPEQAQAILELRLHRLTGLEHEKLLSEYQ**

3 DFU16 100.0% 99.9% **SPTPAEAKERLIATAWESSAVEAMVERAGADACRPEDLDPQYGLRDGKYYLSPEQAQAILELRLHRLTGLEHEKLLSEYQ**

4 DFU48 100.0% 100.0% **SPTPAEAKERLIATAWESSAVEAMVERAGADACRPEDLDPQYGLRDGKYYLSPEQAQAILELRLHRLTGLEHEKLLSEYQ**

5 DFU58 100.0% 100.0% **SPTPAEAKERLIATAWESSAVEAMVERAGADACRPEDLDPQYGLRDGKYYLSPEQAQAILELRLHRLTGLEHEKLLSEYQ**

6 PAO1 100.0% 99.9% **SPTPAEAKERLIATAWESSAVEAMVERAGADACRPEDLDPQYGLRDGKYYLSPEQAQAILELRLHRLTGLEHEKLLSEYQ**

7 ATCC33363 100.0% 99.9% **SPTPAEAKERLIATAWESSAVEAMVERAGADACRPEDLDPQYGLRDGKYYLSPEQAQAILELRLHRLTGLEHEKLLSEYQ**

8 ZBX-P25 100.0% 99.9% **SPTPAEAKERLIATAWESSAVEAMVERAGADACRPEDLDPQYGLRDGKYYLSPEQAQAILELRLHRLTGLEHEKLLSEYQ**

9 DMC-20C 100.0% 100.0% **SPTPAEAKERLIATAWESSAVEAMVERAGADACRPEDLDPQYGLRDGKYYLSPEQAQAILELRLHRLTGLEHEKLLSEYQ**

10 P8W 100.0% 100.0% **SPTPAEAKERLIATAWESSAVEAMVERAGADACRPEDLDPQYGLRDGKYYLSPEQAQAILELRLHRLTGLEHEKLLSEYQ**

11 UTDF19-28A 100.0% 99.9% **SPTPAEAKERLIATAWESSAVEAMVERAGADACRPEDLDPQYGLRDGKYYLSPEQAQAILELRLHRLTGLEHEKLLSEYQ**

12 CMPL223 99.8% 99.2% **SPTPAEAKERLIATAWESSAVEAMVERAGADACRPEDLDPQYGLRDGKYYLSPEQAQAILELRLHRLTGLEHEKLLSEYQ**

13 AS_23 100.0% 100.0% **SPTPAEAKERLIATAWESSAVEAMVERAGADACRPEDLDPQYGLRDGKYYLSPEQAQAILELRLHRLTGLEHEKLLSEYQ**

14 PS1972 100.0% 99.9% **SPTPAEAKERLIATAWESSAVEAMVERAGADACRPEDLDPQYGLRDGKYYLSPEQAQAILELRLHRLTGLEHEKLLSEYQ**

15 BA10747 100.0% 100.0% **SPTPAEAKERLIATAWESSAVEAMVERAGADACRPEDLDPQYGLRDGKYYLSPEQAQAILELRLHRLTGLEHEKLLSEYQ**

16 ST773 100.0% 100.0% **SPTPAEAKERLIATAWESSAVEAMVERAGADACRPEDLDPQYGLRDGKYYLSPEQAQAILELRLHRLTGLEHEKLLSEYQ**

17 DSM50071 100.0% 99.9% **SPTPAEAKERLIATAWESSAVEAMVERAGADACRPEDLDPQYGLRDGKYYLSPEQAQAILELRLHRLTGLEHEKLLSEYQ**

consensus/100% **SPTPAEAKERLIATAWESSAVEAMVERAGADACRPEDLDPQYGLRDGKYYLSPEQAQAILELRLHRLTGLEHEKLLSEYQ**

consensus/90% **SPTPAEAKERLIATAWESSAVEAMVERAGADACRPEDLDPQYGLRDGKYYLSPEQAQAILELRLHRLTGLEHEKLLSEYQ**

consensus/80% **SPTPAEAKERLIATAWESSAVEAMVERAGADACRPEDLDPQYGLRDGKYYLSPEQAQAILELRLHRLTGLEHEKLLSEYQ**

consensus/70% **SPTPAEAKERLIATAWESSAVEAMVERAGADACRPEDLDPQYGLRDGKYYLSPEQAQAILELRLHRLTGLEHEKLLSEYQ**

cov pid **481**  **. 5 . . . . : .** **560**

1 DFU7 100.0% 100.0% **EILNLIGELIRILTNPARLMEVIREELEAVKAEFGDARRTEIVASQVDLTIADLITEEDRVVTISHGGYAKSQPLAAYQA**

2 DFU9 100.0% 99.9% **EILNLIGELIRILTNPARLMEVIREELEAVKAEFGDARRTEIVASQVDLTIADLITEEDRVVTISHGGYAKSQPLAAYQA**

3 DFU16 100.0% 99.9% **EILNLIGELIRILTNPARLMEVIREELEAVKAEFGDARRTEIVASQVDLTIADLITEEDRVVTISHGGYAKSQPLAAYQA**

4 DFU48 100.0% 100.0% **EILNLIGELIRILTNPARLMEVIREELEAVKAEFGDARRTEIVASQVDLTIADLITEEDRVVTISHGGYAKSQPLAAYQA**

5 DFU58 100.0% 100.0% **EILNLIGELIRILTNPARLMEVIREELEAVKAEFGDARRTEIVASQVDLTIADLITEEDRVVTISHGGYAKSQPLAAYQA**

6 PAO1 100.0% 99.9% **EILNLIGELIRILTNPARLMEVIREELEAVKAEFGDARRTEIVASQVDLTIADLITEEDRVVTISHGGYAKSQPLAAYQA**

7 ATCC33363 100.0% 99.9% **EILNLIGELIRILTNPARLMEVIREELEAVKAEFGDARRTEIVASQVDLTIADLITEEDRVVTISHGGYAKSQPLAAYQA**

8 ZBX-P25 100.0% 99.9% **EILNLIGELIRILTNPARLMEVIREELEAVKAEFGDARRTEIVASQVDLTIADLITEEDRVVTISHGGYAKSQPLAAYQA**

9 DMC-20C 100.0% 100.0% **EILNLIGELIRILTNPARLMEVIREELEAVKAEFGDARRTEIVASQVDLTIADLITEEDRVVTISHGGYAKSQPLAAYQA**

10 P8W 100.0% 100.0% **EILNLIGELIRILTNPARLMEVIREELEAVKAEFGDARRTEIVASQVDLTIADLITEEDRVVTISHGGYAKSQPLAAYQA**

11 UTDF19-28A 100.0% 99.9% **EILNLIGELIRILTNPARLMEVIREELEAVKAEFGDARRTEIVASQVDLTIADLITEEDRVVTISHGGYAKSQPLAAYQA**

12 CMPL223 99.8% 99.2% **EILNLIGELIRILTNPARLMEVIREELEAVKAEFGDARRTEIVASQVDLTIADLITEEDRVVTISHGGYAKSQPLAAYQA**

13 AS_23 100.0% 100.0% **EILNLIGELIRILTNPARLMEVIREELEAVKAEFGDARRTEIVASQVDLTIADLITEEDRVVTISHGGYAKSQPLAAYQA**

14 PS1972 100.0% 99.9% **EILNLIGELIRILTNPARLMEVIREELEAVKAEFGDARRTEIVASQVDLTIADLITEEDRVVTISHGGYAKSQPLAAYQA**

15 BA10747 100.0% 100.0% **EILNLIGELIRILTNPARLMEVIREELEAVKAEFGDARRTEIVASQVDLTIADLITEEDRVVTISHGGYAKSQPLAAYQA**

16 ST773 100.0% 100.0% **EILNLIGELIRILTNPARLMEVIREELEAVKAEFGDARRTEIVASQVDLTIADLITEEDRVVTISHGGYAKSQPLAAYQA**

17 DSM50071 100.0% 99.9% **EILNLIGELIRILTNPARLMEVIREELEAVKAEFGDARRTEIVASQVDLTIADLITEEDRVVTISHGGYAKSQPLAAYQA**

consensus/100% **EILNLIGELIRILTNPARLMEVIREELEAVKAEFGDARRTEIVASQVDLTIADLITEEDRVVTISHGGYAKSQPLAAYQA**

consensus/90% **EILNLIGELIRILTNPARLMEVIREELEAVKAEFGDARRTEIVASQVDLTIADLITEEDRVVTISHGGYAKSQPLAAYQA**

consensus/80% **EILNLIGELIRILTNPARLMEVIREELEAVKAEFGDARRTEIVASQVDLTIADLITEEDRVVTISHGGYAKSQPLAAYQA**

consensus/70% **EILNLIGELIRILTNPARLMEVIREELEAVKAEFGDARRTEIVASQVDLTIADLITEEDRVVTISHGGYAKSQPLAAYQA**

cov pid **561**  **. . . 6 . . . .** **640**

1 DFU7 100.0% 100.0% **QRRGGKGKSATGMKDEDYIEHLLVANSHATLLLFSSKGKVYWLRTFEIPEASRTARGRPLVNLLPLDEGERITAMLQIDL**

2 DFU9 100.0% 99.9% **QRRGGKGKSATGMKDEDYIEHLLVANSHATLLLFSSKGKVYWLRTFEIPEASRTARGRPLVNLLPLDEGERITAMLQIDL**

3 DFU16 100.0% 99.9% **QRRGGKGKSATGMKDEDYIEHLLVANSHATLLLFSSKGKVYWLRTFEIPEASRTARGRPLVNLLPLDEGERITAMLQIDL**

4 DFU48 100.0% 100.0% **QRRGGKGKSATGMKDEDYIEHLLVANSHATLLLFSSKGKVYWLRTFEIPEASRTARGRPLVNLLPLDEGERITAMLQIDL**

5 DFU58 100.0% 100.0% **QRRGGKGKSATGMKDEDYIEHLLVANSHATLLLFSSKGKVYWLRTFEIPEASRTARGRPLVNLLPLDEGERITAMLQIDL**

6 PAO1 100.0% 99.9% **QRRGGKGKSATGMKDEDYIEHLLVANSHATLLLFSSKGKVYWLRTFEIPEASRTARGRPLVNLLPLDEGERITAMLQIDL**

7 ATCC33363 100.0% 99.9% **QRRGGKGKSATGMKDEDYIEHLLVANSHATLLLFSSKGKVYWLRTFEIPEASRTARGRPLVNLLPLDEGERITAMLQIDL**

8 ZBX-P25 100.0% 99.9% **QRRGGKGKSATGMKDEDYIEHLLVANSHATLLLFSSKGKVYWLRTFEIPEASRTARGRPLVNLLPLDEGERITAMLQIDL**

9 DMC-20C 100.0% 100.0% **QRRGGKGKSATGMKDEDYIEHLLVANSHATLLLFSSKGKVYWLRTFEIPEASRTARGRPLVNLLPLDEGERITAMLQIDL**

10 P8W 100.0% 100.0% **QRRGGKGKSATGMKDEDYIEHLLVANSHATLLLFSSKGKVYWLRTFEIPEASRTARGRPLVNLLPLDEGERITAMLQIDL**

11 UTDF19-28A 100.0% 99.9% **QRRGGKGKSATGMKDEDYIEHLLVANSHATLLLFSSKGKVYWLRTFEIPEASRTARGRPLVNLLPLDEGERITAMLQIDL**

12 CMPL223 99.8% 99.2% **QRRGGKGKSATGMKDEDYIEHLLVANSHATLLLFSSKGKVYWLRTFEIPEASRTARGRPLVNLLPLDEGERITAMLQIDL**

13 AS_23 100.0% 100.0% **QRRGGKGKSATGMKDEDYIEHLLVANSHATLLLFSSKGKVYWLRTFEIPEASRTARGRPLVNLLPLDEGERITAMLQIDL**

14 PS1972 100.0% 99.9% **QRRGGKGKSATGMKDEDYIEHLLVANSHATLLLFSSKGKVYWLRTFEIPEASRTARGRPLVNLLPLDEGERITAMLQIDL**

15 BA10747 100.0% 100.0% **QRRGGKGKSATGMKDEDYIEHLLVANSHATLLLFSSKGKVYWLRTFEIPEASRTARGRPLVNLLPLDEGERITAMLQIDL**

16 ST773 100.0% 100.0% **QRRGGKGKSATGMKDEDYIEHLLVANSHATLLLFSSKGKVYWLRTFEIPEASRTARGRPLVNLLPLDEGERITAMLQIDL**

17 DSM50071 100.0% 99.9% **QRRGGKGKSATGMKDEDYIEHLLVANSHATLLLFSSKGKVYWLRTFEIPEASRTARGRPLVNLLPLDEGERITAMLQIDL**

consensus/100% **QRRGGKGKSATGMKDEDYIEHLLVANSHATLLLFSSKGKVYWLRTFEIPEASRTARGRPLVNLLPLDEGERITAMLQIDL**

consensus/90% **QRRGGKGKSATGMKDEDYIEHLLVANSHATLLLFSSKGKVYWLRTFEIPEASRTARGRPLVNLLPLDEGERITAMLQIDL**

consensus/80% **QRRGGKGKSATGMKDEDYIEHLLVANSHATLLLFSSKGKVYWLRTFEIPEASRTARGRPLVNLLPLDEGERITAMLQIDL**

consensus/70% **QRRGGKGKSATGMKDEDYIEHLLVANSHATLLLFSSKGKVYWLRTFEIPEASRTARGRPLVNLLPLDEGERITAMLQIDL**

cov pid **641**  **: . . . . 7 . .** **720**

1 DFU7 100.0% 100.0% **EALQQNGGADDDLDEAEGAVLEGEVVEAAEVEEVEGETAELVAEPTGAYIFMATAFGTVKKTPLVQFSRPRSSGLIALKL**

2 DFU9 100.0% 99.9% **EALQQNGGADDDLDEAEGAVLEGEVVEAAEVEEVEGETAELVAEPTGAYIFMATAFGTVKKTPLVQFSRPRSSGLIALKL**

3 DFU16 100.0% 99.9% **EALQQNGGADDDLDEAEGAVLEGEVVEAAEVEEVEGETAELVAEPTGAYIFMATAFGTVKKTPLVQFSRPRSSGLIALKL**

4 DFU48 100.0% 100.0% **EALQQNGGADDDLDEAEGAVLEGEVVEAAEVEEVEGETAELVAEPTGAYIFMATAFGTVKKTPLVQFSRPRSSGLIALKL**

5 DFU58 100.0% 100.0% **EALQQNGGADDDLDEAEGAVLEGEVVEAAEVEEVEGETAELVAEPTGAYIFMATAFGTVKKTPLVQFSRPRSSGLIALKL**

6 PAO1 100.0% 99.9% **EALQQNGGADDDLDEAEGAVLEGEVVEAAEVEEVEGETAELVAEPTGAYIFMATAFGTVKKTPLVQFSRPRSSGLIALKL**

7 ATCC33363 100.0% 99.9% **EALQQNGGADDDLDEAEGAVLEGEVVEAAEVEEVEGETAELVAEPTGAYIFMATAFGTVKKTPLVQFSRPRSSGLIALKL**

8 ZBX-P25 100.0% 99.9% **EALQQNGGADDDLDEAEGAVLEGEVVEAAEVEEVEGETAELVAEPTGAYIFMATAFGTVKKTPLVQFSRPRSSGLIALKL**

9 DMC-20C 100.0% 100.0% **EALQQNGGADDDLDEAEGAVLEGEVVEAAEVEEVEGETAELVAEPTGAYIFMATAFGTVKKTPLVQFSRPRSSGLIALKL**

10 P8W 100.0% 100.0% **EALQQNGGADDDLDEAEGAVLEGEVVEAAEVEEVEGETAELVAEPTGAYIFMATAFGTVKKTPLVQFSRPRSSGLIALKL**

11 UTDF19-28A 100.0% 99.9% **EALQQNGGADDDLDEAEGAVLEGEVVEAAEVEEVEGETAELVAEPTGAYIFMATAFGTVKKTPLVQFSRPRSSGLIALKL**

12 CMPL223 99.8% 99.2% **EALQQNGGADDDLDEAEGAVLEGEVVEAAEIEEVEGETAELVAEPTGAYIFMATAFGTVKKTPLVQFSRPRSSGLIALKL**

13 AS_23 100.0% 100.0% **EALQQNGGADDDLDEAEGAVLEGEVVEAAEVEEVEGETAELVAEPTGAYIFMATAFGTVKKTPLVQFSRPRSSGLIALKL**

14 PS1972 100.0% 99.9% **EALQQNGGADDDLDEAEGAVLEGEVVEAAEVEEVEGETAELVAEPTGAYIFMATAFGTVKKTPLVQFSRPRSSGLIALKL**

15 BA10747 100.0% 100.0% **EALQQNGGADDDLDEAEGAVLEGEVVEAAEVEEVEGETAELVAEPTGAYIFMATAFGTVKKTPLVQFSRPRSSGLIALKL**

16 ST773 100.0% 100.0% **EALQQNGGADDDLDEAEGAVLEGEVVEAAEVEEVEGETAELVAEPTGAYIFMATAFGTVKKTPLVQFSRPRSSGLIALKL**

17 DSM50071 100.0% 99.9% **EALQQNGGADDDLDEAEGAVLEGEVVEAAEVEEVEGETAELVAEPTGAYIFMATAFGTVKKTPLVQFSRPRSSGLIALKL**

consensus/100% **EALQQNGGADDDLDEAEGAVLEGEVVEAAElEEVEGETAELVAEPTGAYIFMATAFGTVKKTPLVQFSRPRSSGLIALKL**

consensus/90% **EALQQNGGADDDLDEAEGAVLEGEVVEAAEVEEVEGETAELVAEPTGAYIFMATAFGTVKKTPLVQFSRPRSSGLIALKL**

consensus/80% **EALQQNGGADDDLDEAEGAVLEGEVVEAAEVEEVEGETAELVAEPTGAYIFMATAFGTVKKTPLVQFSRPRSSGLIALKL**

consensus/70% **EALQQNGGADDDLDEAEGAVLEGEVVEAAEVEEVEGETAELVAEPTGAYIFMATAFGTVKKTPLVQFSRPRSSGLIALKL**

cov pid **721**  **. . : . . . . 8** **800**

1 DFU7 100.0% 100.0% **EEGDTLIAAAITDGAKEVMLFSSAGKVIRFAESVVRIMGRNARGVRGMRLGKGQQLISMLIPESGAQILTASERGFGKRT**

2 DFU9 100.0% 99.9% **EEGDTLIAAAITDGAKEVMLFSSAGKVIRFAESVVRIMGRNARGVRGMRLGKGQQLISMLIPESGAQILTASERGFGKRT**

3 DFU16 100.0% 99.9% **EEGDTLIAAAITDGAKEVMLFSSAGKVIRFAESVVRIMGRNARGVRGMRLGKGQQLISMLIPESGAQILTASERGFGKRT**

4 DFU48 100.0% 100.0% **EEGDTLIAAAITDGAKEVMLFSSAGKVIRFAESVVRIMGRNARGVRGMRLGKGQQLISMLIPESGAQILTASERGFGKRT**

5 DFU58 100.0% 100.0% **EEGDTLIAAAITDGAKEVMLFSSAGKVIRFAESVVRIMGRNARGVRGMRLGKGQQLISMLIPESGAQILTASERGFGKRT**

6 PAO1 100.0% 99.9% **EEGDTLIAAAITDGAKEVMLFSSAGKVIRFAESVVRIMGRNARGVRGMRLGKGQQLISMLIPESGAQILTASERGFGKRT**

7 ATCC33363 100.0% 99.9% **EEGDTLIAAAITDGAKEVMLFSSAGKVIRFAESVVRIMGRNARGVRGMRLGKGQQLISMLIPESGAQILTASERGFGKRT**

8 ZBX-P25 100.0% 99.9% **EEGDTLIAAAITDGAKEVMLFSSAGKVIRFAESVVRIMGRNARGVRGMRLGKGQQLISMLIPESGAQILTASERGFGKRT**

9 DMC-20C 100.0% 100.0% **EEGDTLIAAAITDGAKEVMLFSSAGKVIRFAESVVRIMGRNARGVRGMRLGKGQQLISMLIPESGAQILTASERGFGKRT**

10 P8W 100.0% 100.0% **EEGDTLIAAAITDGAKEVMLFSSAGKVIRFAESVVRIMGRNARGVRGMRLGKGQQLISMLIPESGAQILTASERGFGKRT**

11 UTDF19-28A 100.0% 99.9% **EEGDTLIAAAITDGAKEVMLFSSAGKVIRFAESVVRIMGRNARGVRGMRLGKGQQLISMLIPESGAQILTASERGFGKRT**

12 CMPL223 99.8% 99.2% **EEGDTLIAAAITDGAKEVMLFSSAGKVIRFAESVVRIMGRNARGVRGMRLGKGQQLISMLIPESGAQILTASERGFGKRT**

13 AS_23 100.0% 100.0% **EEGDTLIAAAITDGAKEVMLFSSAGKVIRFAESVVRIMGRNARGVRGMRLGKGQQLISMLIPESGAQILTASERGFGKRT**

14 PS1972 100.0% 99.9% **EEGDTLIAAAITDGAKEVMLFSSAGKVIRFAESVVRIMGRNARGVRGMRLGKGQQLISMLIPESGAQILTASERGFGKRT**

15 BA10747 100.0% 100.0% **EEGDTLIAAAITDGAKEVMLFSSAGKVIRFAESVVRIMGRNARGVRGMRLGKGQQLISMLIPESGAQILTASERGFGKRT**

16 ST773 100.0% 100.0% **EEGDTLIAAAITDGAKEVMLFSSAGKVIRFAESVVRIMGRNARGVRGMRLGKGQQLISMLIPESGAQILTASERGFGKRT**

17 DSM50071 100.0% 99.9% **EEGDTLIAAAITDGAKEVMLFSSAGKVIRFAESVVRIMGRNARGVRGMRLGKGQQLISMLIPESGAQILTASERGFGKRT**

consensus/100% **EEGDTLIAAAITDGAKEVMLFSSAGKVIRFAESVVRIMGRNARGVRGMRLGKGQQLISMLIPESGAQILTASERGFGKRT**

consensus/90% **EEGDTLIAAAITDGAKEVMLFSSAGKVIRFAESVVRIMGRNARGVRGMRLGKGQQLISMLIPESGAQILTASERGFGKRT**

consensus/80% **EEGDTLIAAAITDGAKEVMLFSSAGKVIRFAESVVRIMGRNARGVRGMRLGKGQQLISMLIPESGAQILTASERGFGKRT**

consensus/70% **EEGDTLIAAAITDGAKEVMLFSSAGKVIRFAESVVRIMGRNARGVRGMRLGKGQQLISMLIPESGAQILTASERGFGKRT**

cov pid **801**  **. . . . : . . .** **880**

1 DFU7 100.0% 100.0% **PLSKFPRRGRGGQGVIAMVTNERNGALIAAVQVQEGEEIMLISDQGTLVRTRVDEVSLSGRNTQGVTLIKLASDEVLVGL**

2 DFU9 100.0% 99.9% **PLSKFPRRGRGGQGVIAMVTNERNGALIAAVQVQEGEEIMLISDQGTLVRTRVDEVSLSGRNTQGVTLIKLASDEVLVGL**

3 DFU16 100.0% 99.9% **PLSKFPRRGRGGQGVIAMVTNERNGALIAAVQVQEGEEIMLISDQGTLVRTRVDEVSLSGRNTQGVTLIKLASDEVLVGL**

4 DFU48 100.0% 100.0% **PLSKFPRRGRGGQGVIAMVTNERNGALIAAVQVQEGEEIMLISDQGTLVRTRVDEVSLSGRNTQGVTLIKLASDEVLVGL**

5 DFU58 100.0% 100.0% **PLSKFPRRGRGGQGVIAMVTNERNGALIAAVQVQEGEEIMLISDQGTLVRTRVDEVSLSGRNTQGVTLIKLASDEVLVGL**

6 PAO1 100.0% 99.9% **PLSKFPRRGRGGQGVIAMVTNERNGALIAAVQVQEGEEIMLISDQGTLVRTRVDEVSLSGRNTQGVTLIKLASDEVLVGL**

7 ATCC33363 100.0% 99.9% **PLSKFPRRGRGGQGVIAMVTNERNGALIAAVQVQEGEEIMLISDQGTLVRTRVDEVSLSGRNTQGVTLIKLASDEVLVGL**

8 ZBX-P25 100.0% 99.9% **PLSKFPRRGRGGQGVIAMVTNERNGALIAAVQVQEGEEIMLISDQGTLVRTRVDEVSLSGRNTQGVTLIKLASDEVLVGL**

9 DMC-20C 100.0% 100.0% **PLSKFPRRGRGGQGVIAMVTNERNGALIAAVQVQEGEEIMLISDQGTLVRTRVDEVSLSGRNTQGVTLIKLASDEVLVGL**

10 P8W 100.0% 100.0% **PLSKFPRRGRGGQGVIAMVTNERNGALIAAVQVQEGEEIMLISDQGTLVRTRVDEVSLSGRNTQGVTLIKLASDEVLVGL**

11 UTDF19-28A 100.0% 99.9% **PLSKFPRRGRGGQGVIAMVTNERNGALIAAVQVQEGEEIMLISDQGTLVRTRVDEVSLSGRNTQGVTLIKLASDEVLVGL**

12 CMPL223 99.8% 99.2% **PLSKFPRRGRGGQGVIAMVTNERNGALIAAVQVQEGEEIMLISDQGTLVRTRVDEVSLSSRNTQGVTLIKLASDEVLVGL**

13 AS_23 100.0% 100.0% **PLSKFPRRGRGGQGVIAMVTNERNGALIAAVQVQEGEEIMLISDQGTLVRTRVDEVSLSGRNTQGVTLIKLASDEVLVGL**

14 PS1972 100.0% 99.9% **PLSKFPRRGRGGQGVIAMVTNERNGALIAAVQVQEGEEIMLISDQGTLVRTRVDEVSLSGRNTQGVTLIKLASDEVLVGL**

15 BA10747 100.0% 100.0% **PLSKFPRRGRGGQGVIAMVTNERNGALIAAVQVQEGEEIMLISDQGTLVRTRVDEVSLSGRNTQGVTLIKLASDEVLVGL**

16 ST773 100.0% 100.0% **PLSKFPRRGRGGQGVIAMVTNERNGALIAAVQVQEGEEIMLISDQGTLVRTRVDEVSLSGRNTQGVTLIKLASDEVLVGL**

17 DSM50071 100.0% 99.9% **PLSKFPRRGRGGQGVIAMVTNERNGALIAAVQVQEGEEIMLISDQGTLVRTRVDEVSLSGRNTQGVTLIKLASDEVLVGL**

consensus/100% **PLSKFPRRGRGGQGVIAMVTNERNGALIAAVQVQEGEEIMLISDQGTLVRTRVDEVSLSuRNTQGVTLIKLASDEVLVGL**

consensus/90% **PLSKFPRRGRGGQGVIAMVTNERNGALIAAVQVQEGEEIMLISDQGTLVRTRVDEVSLSGRNTQGVTLIKLASDEVLVGL**

consensus/80% **PLSKFPRRGRGGQGVIAMVTNERNGALIAAVQVQEGEEIMLISDQGTLVRTRVDEVSLSGRNTQGVTLIKLASDEVLVGL**

consensus/70% **PLSKFPRRGRGGQGVIAMVTNERNGALIAAVQVQEGEEIMLISDQGTLVRTRVDEVSLSGRNTQGVTLIKLASDEVLVGL**

cov pid **881**  **. 9 . . ]** **923**

1 DFU7 100.0% 100.0% **ERVQEPSGGDDEDLPEGEEAAESLGESAESESEPAAEAEGNEE**

2 DFU9 100.0% 99.9% **ERVQEPSGGDDEDLPEGEEAAESLGESAESESEPAAEAEGNEE**

3 DFU16 100.0% 99.9% **ERVQEPSGGDDEDLPEGEEAAESLGESAESESEPAAEAEGNEE**

4 DFU48 100.0% 100.0% **ERVQEPSGGDDEDLPEGEEAAESLGESAESESEPAAEAEGNEE**

5 DFU58 100.0% 100.0% **ERVQEPSGGDDEDLPEGEEAAESLGESAESESEPAAEAEGNEE**

6 PAO1 100.0% 99.9% **ERVQEPSGGDDEDLPEGEEAAESLGESAESESEPAAEAEGNEE**

7 ATCC33363 100.0% 99.9% **ERVQEPSGGDDEDLPEGEEAAESLGESAESESEPAAEAEGNEE**

8 ZBX-P25 100.0% 99.9% **ERVQEPSGGDDEDLPEGEEAAESLGESAESESEPAAEAEGNEE**

9 DMC-20C 100.0% 100.0% **ERVQEPSGGDDEDLPEGEEAAESLGESAESESEPAAEAEGNEE**

10 P8W 100.0% 100.0% **ERVQEPSGGDDEDLPEGEEAAESLGESAESESEPAAEAEGNEE**

11 UTDF19-28A 100.0% 99.9% **ERVQEPSGGDDEDLPEGEEAAESLGESAESESEPAAEAEGNEE**

12 CMPL223 99.8% 99.2% **ERVQEPSGGDDEELPEGEEGAEALGESAES--EPAAEAEGNEE**

13 AS_23 100.0% 100.0% **ERVQEPSGGDDEDLPEGEEAAESLGESAESESEPAAEAEGNEE**

14 PS1972 100.0% 99.9% **ERVQEPSGGDDEDLPEGEEAAESLGESAESESEPAAEAEGNEE**

15 BA10747 100.0% 100.0% **ERVQEPSGGDDEDLPEGEEAAESLGESAESESEPAAEAEGNEE**

16 ST773 100.0% 100.0% **ERVQEPSGGDDEDLPEGEEAAESLGESAESESEPAAEAEGNEE**

17 DSM50071 100.0% 99.9% **ERVQEPSGGDDEDLPEGEEAAESLGESAESESEPAAEAEGNEE**

consensus/100% **ERVQEPSGGDDE-LPEGEEuAEuLGESAES..EPAAEAEGNEE**

consensus/90% **ERVQEPSGGDDEDLPEGEEAAESLGESAESESEPAAEAEGNEE**

consensus/80% **ERVQEPSGGDDEDLPEGEEAAESLGESAESESEPAAEAEGNEE**

consensus/70% **ERVQEPSGGDDEDLPEGEEAAESLGESAESESEPAAEAEGNEE**

**Supplementary Figure 2.** Multiple sequence alignment of the predicted amino acid sequences of GyrA carried by P. aeruginosa isolates and close genomes retrieved from the BV-BRC database compared to that P. aeruginosa strains (PAO1 and ATCC^®^33363). The alignment was visualized using MView version 1.63 hosted by the EMBL-EBI; cov, coverage; pid, percent identity.

cov pid  **1** **[ . . . . : . . .** **80**

1 DFU7 100.0% 100.0% **MSESLDLSLEGVERRSLAEFTEQAYLNYSMYVIMDRALPHIGDGLKPVQRRIVYAMSELGLDADSKHKKSARTVGDVLGK**

2 DFU9 100.0% 99.9% **MSESLDLSLEGVERRSLAEFTEQAYLNYSMYVIMDRALPHIGDGLKPVQRRIVYAMSELGLDADSKHKKSARTVGDVLGK**

3 DFU16 100.0% 99.9% **MSESLDLSLEGVERRSLAEFTEQAYLNYSMYVIMDRALPHIGDGLKPVQRRIVYAMSELGLDADSKHKKSARTVGDVLGK**

4 DFU48 100.0% 100.0% **MSESLDLSLEGVERRSLAEFTEQAYLNYSMYVIMDRALPHIGDGLKPVQRRIVYAMSELGLDADSKHKKSARTVGDVLGK**

5 DFU58 100.0% 99.9% **MSESLDLSLEGVERRSLAEFTEQAYLNYSMYVIMDRALPHIGDGLKPVQRRIVYAMSELGLDADSKHKKSARTVGDVLGK**

6 PAO1 100.0% 99.9% **MSESLDLSLEGVERRSLAEFTEQAYLNYSMYVIMDRALPHIGDGLKPVQRRIVYAMSELGLDADSKHKKSARTVGDVLGK**

7 ATCC33363 100.0% 99.7% **MSESLDLSLEGVERRSLAEFTEQAYLNYSMYVIMDRALPHIGDGLKPVQRRIVYAMSELGLDADSKHKKSARTVGDVLGK**

8 ZBX-P25 100.0% 99.9% **MSESLDLSLEGVERRSLAEFTEQAYLNYSMYVIMDRALPHIGDGLKPVQRRIVYAMSELGLDADSKHKKSARTVGDVLGK**

9 DMC-20C 100.0% 99.9% **MSESLDLSLEGVERRSLAEFTEQAYLNYSMYVIMDRALPHIGDGLKPVQRRIVYAMSELGLDADSKHKKSARTVGDVLGK**

10 P8W 100.0% 99.9% **MSESLDLSLEGVERRSLAEFTEQAYLNYSMYVIMDRALPHIGDGLKPVQRRIVYAMSELGLDADSKHKKSARTVGDVLGK**

11 UTDF19 100.0% 99.7% **MSESLDLSLEGVERRSLAEFTEQAYLNYSMYVIMDRALPHIGDGLKPVQRRIVYAMSELGLDADSKHKKSARTVGDVLGK**

12 CMPL223 100.0% 99.9% **MSESLDLSLEGVERRSLAEFTEQAYLNYSMYVIMDRALPHIGDGLKPVQRRIVYAMSELGLDADSKHKKSARTVGDVLGK**

13 AS_23 100.0% 100.0% **MSESLDLSLEGVERRSLAEFTEQAYLNYSMYVIMDRALPHIGDGLKPVQRRIVYAMSELGLDADSKHKKSARTVGDVLGK**

14 PS1972 100.0% 99.7% **MSESLDLSLEGVERRSLAEFTEQAYLNYSMYVIMDRALPHIGDGLKPVQRRIVYAMSELGLDADSKHKKSARTVGDVLGK**

15 BA10747 100.0% 100.0% **MSESLDLSLEGVERRSLAEFTEQAYLNYSMYVIMDRALPHIGDGLKPVQRRIVYAMSELGLDADSKHKKSARTVGDVLGK**

16 ST773 100.0% 100.0% **MSESLDLSLEGVERRSLAEFTEQAYLNYSMYVIMDRALPHIGDGLKPVQRRIVYAMSELGLDADSKHKKSARTVGDVLGK**

17 DSM50071 100.0% 99.7% **MSESLDLSLEGVERRSLAEFTEQAYLNYSMYVIMDRALPHIGDGLKPVQRRIVYAMSELGLDADSKHKKSARTVGDVLGK**

consensus/100% **MSESLDLSLEGVERRSLAEFTEQAYLNYSMYVIMDRALPHIGDGLKPVQRRIVYAMSELGLDADSKHKKSARTVGDVLGK**

consensus/90% **MSESLDLSLEGVERRSLAEFTEQAYLNYSMYVIMDRALPHIGDGLKPVQRRIVYAMSELGLDADSKHKKSARTVGDVLGK**

consensus/80% **MSESLDLSLEGVERRSLAEFTEQAYLNYSMYVIMDRALPHIGDGLKPVQRRIVYAMSELGLDADSKHKKSARTVGDVLGK**

consensus/70% **MSESLDLSLEGVERRSLAEFTEQAYLNYSMYVIMDRALPHIGDGLKPVQRRIVYAMSELGLDADSKHKKSARTVGDVLGK**

cov pid  **81**  **. 1 . . . . : .** **160**

1 DFU7 100.0% 100.0% **FHPHGDLACYEAMVLMAQPFSYRYPLVDGQGNWGAPDDPKSFAAMRYTEARLSRYSEVLLSELGQGTVDWVPNFDGTLDE**

2 DFU9 100.0% 99.9% **FHPHGDSACYEAMVLMAQPFSYRYPLVDGQGNWGAPDDPKSFAAMRYTEARLSRYSEVLLSELGQGTVDWVPNFDGTLDE**

3 DFU16 100.0% 99.9% **FHPHGDSACYEAMVLMAQPFSYRYPLVDGQGNWGAPDDPKSFAAMRYTEARLSRYSEVLLSELGQGTVDWVPNFDGTLDE**

4 DFU48 100.0% 100.0% **FHPHGDLACYEAMVLMAQPFSYRYPLVDGQGNWGAPDDPKSFAAMRYTEARLSRYSEVLLSELGQGTVDWVPNFDGTLDE**

5 DFU58 100.0% 99.9% **FHPHGDLACYEAMVLMAQPFSYRYPLVDGQGNWGAPDDPKSFAAMRYTEARLSRYSEVLLSELGQGTVDWVPNFDGTLDE**

6 PAO1 100.0% 99.9% **FHPHGDSACYEAMVLMAQPFSYRYPLVDGQGNWGAPDDPKSFAAMRYTEARLSRYSEVLLSELGQGTVDWVPNFDGTLDE**

7 ATCC33363 100.0% 99.7% **FHPHGDSACYEAMVLMAQPFSYRYPLVDGQGNWGAPDDPKSFAAMRYTEARLSRYSEVLLSELGQGTVDWVPNFDGTLDE**

8 ZBX-P25 100.0% 99.9% **FHPHGDLACYEAMVLMAQPFSYRYPLVDGQGNWGAPDDPKSFAAMRYTEARLSRYSEVLLSELGQGTVDWVPNFDGTLDE**

9 DMC-20C 100.0% 99.9% **FHPHGDLACYEAMVLMAQPFSYRYPLVDGQGNWGAPDDPKSFAAMRYTEARLSRYSEVLLSELGQGTVDWVPNFDGTLDE**

10 P8W 100.0% 99.9% **FHPHGDLACYEAMVLMAQPFSYRYPLVDGQGNWGAPDDPKSFAAMRYTEARLSRYSEVLLSELGQGTVDWVPNFDGTLDE**

11 UTDF19 100.0% 99.7% **FHPHGDSACYEAMVLMAQPFSYRYPLVDGQGNWGAPDDPKSFAAMRYTEARLSRYSEVLLSELGQGTVDWVPNFDGTLDE**

12 CMPL223 100.0% 99.9% **FHPHGDLACYEAMVLMAQPFSYRYPLVDGQGNWGAPDDPKSFAAMRYTEARLSRYSEVLLSELGQGTVDWVPNFDGTLDE**

13 AS_23 100.0% 100.0% **FHPHGDLACYEAMVLMAQPFSYRYPLVDGQGNWGAPDDPKSFAAMRYTEARLSRYSEVLLSELGQGTVDWVPNFDGTLDE**

14 PS1972 100.0% 99.7% **FHPHGDSACYEAMVLMAQPFSYRYPLVDGQGNWGAPDDPKSFAAMRYTEARLSRYSEVLLSELGQGTVDWVPNFDGTLDE**

15 BA10747 100.0% 100.0% **FHPHGDLACYEAMVLMAQPFSYRYPLVDGQGNWGAPDDPKSFAAMRYTEARLSRYSEVLLSELGQGTVDWVPNFDGTLDE**

16 ST773 100.0% 100.0% **FHPHGDLACYEAMVLMAQPFSYRYPLVDGQGNWGAPDDPKSFAAMRYTEARLSRYSEVLLSELGQGTVDWVPNFDGTLDE**

17 DSM50071 100.0% 99.7% **FHPHGDSACYEAMVLMAQPFSYRYPLVDGQGNWGAPDDPKSFAAMRYTEARLSRYSEVLLSELGQGTVDWVPNFDGTLDE**

consensus/100% **FHPHGD.ACYEAMVLMAQPFSYRYPLVDGQGNWGAPDDPKSFAAMRYTEARLSRYSEVLLSELGQGTVDWVPNFDGTLDE**

consensus/90% **FHPHGD.ACYEAMVLMAQPFSYRYPLVDGQGNWGAPDDPKSFAAMRYTEARLSRYSEVLLSELGQGTVDWVPNFDGTLDE**

consensus/80% **FHPHGD.ACYEAMVLMAQPFSYRYPLVDGQGNWGAPDDPKSFAAMRYTEARLSRYSEVLLSELGQGTVDWVPNFDGTLDE**

consensus/70% **FHPHGD.ACYEAMVLMAQPFSYRYPLVDGQGNWGAPDDPKSFAAMRYTEARLSRYSEVLLSELGQGTVDWVPNFDGTLDE**

cov pid **161**  **. . . 2 . . . .** **240**

1 DFU7 100.0% 100.0% **PAVLPARLPNLLLNGTTGIAVGMATDVPPHNLREVASACVRLLDQPGATVAELCEHVPGPDFPTEAEIITPRADLQKVYE**

2 DFU9 100.0% 99.9% **PAVLPARLPNLLLNGTTGIAVGMATDVPPHNLREVASACVRLLDQPGATVAELCEHVPGPDFPTEAEIITPRADLQKVYE**

3 DFU16 100.0% 99.9% **PAVLPARLPNLLLNGTTGIAVGMATDVPPHNLREVASACVRLLDQPGATVAELCEHVPGPDFPTEAEIITPRADLQKVYE**

4 DFU48 100.0% 100.0% **PAVLPARLPNLLLNGTTGIAVGMATDVPPHNLREVASACVRLLDQPGATVAELCEHVPGPDFPTEAEIITPRADLQKVYE**

5 DFU58 100.0% 99.9% **PAVLPARLPNLLLNGTTGIAVGMATDVPPHNLREVASACVRLLDQPGATVAELCEHVPGPDFPTEAEIITPRADLQKVYE**

6 PAO1 100.0% 99.9% **PAVLPARLPNLLLNGTTGIAVGMATDVPPHNLREVASACVRLLDQPGATVAELCEHVPGPDFPTEAEIITPRADLQKVYE**

7 ATCC33363 100.0% 99.7% **PAVLPARLPNLLLNGTTGIAVGMATDVPPHNLREVASACVRLLDQPGATVAELCEHVPGPDFPTEAEIITPRADLQKVYE**

8 ZBX-P25 100.0% 99.9% **PAVLPARLPNLLLNGTTGIAVGMATDVPPHNLREVASACVRLLDQPGATVAELCEHVPGPDFPTEAEIITPRADLQKVYE**

9 DMC-20C 100.0% 99.9% **PAVLPARLPNLLLNGTTGIAVGMATDVPPHNLREVASACVRLLDQPGATVAELCEHVPGPDFPTEAEIITPRADLQKVYE**

10 P8W 100.0% 99.9% **PAVLPARLPNLLLNGTTGIAVGMATDVPPHNLREVASACVRLLDQPGATVAELCEHVPGPDFPTEAEIITPRADLQKVYE**

11 UTDF19 100.0% 99.7% **PAVLPARLPNLLLNGTTGIAVGMATDVPPHNLREVASACVRLLDQPGATVAELCEHVPGPDFPTEAEIITPRADLQKVYE**

12 CMPL223 100.0% 99.9% **PAVLPARLPNLLLNGTTGIAVGMATDVPPHNLREVASACVRLLDQPGATVAELCEHVPGPDFPTEAEIITPRADLQKVYE**

13 AS_23 100.0% 100.0% **PAVLPARLPNLLLNGTTGIAVGMATDVPPHNLREVASACVRLLDQPGATVAELCEHVPGPDFPTEAEIITPRADLQKVYE**

14 PS1972 100.0% 99.7% **PAVLPARLPNLLLNGTTGIAVGMATDVPPHNLREVASACVRLLDQPGATVAELCEHVPGPDFPTEAEIITPRADLQKVYE**

15 BA10747 100.0% 100.0% **PAVLPARLPNLLLNGTTGIAVGMATDVPPHNLREVASACVRLLDQPGATVAELCEHVPGPDFPTEAEIITPRADLQKVYE**

16 ST773 100.0% 100.0% **PAVLPARLPNLLLNGTTGIAVGMATDVPPHNLREVASACVRLLDQPGATVAELCEHVPGPDFPTEAEIITPRADLQKVYE**

17 DSM50071 100.0% 99.7% **PAVLPARLPNLLLNGTTGIAVGMATDVPPHNLREVASACVRLLDQPGATVAELCEHVPGPDFPTEAEIITPRADLQKVYE**

consensus/100% **PAVLPARLPNLLLNGTTGIAVGMATDVPPHNLREVASACVRLLDQPGATVAELCEHVPGPDFPTEAEIITPRADLQKVYE**

consensus/90% **PAVLPARLPNLLLNGTTGIAVGMATDVPPHNLREVASACVRLLDQPGATVAELCEHVPGPDFPTEAEIITPRADLQKVYE**

consensus/80% **PAVLPARLPNLLLNGTTGIAVGMATDVPPHNLREVASACVRLLDQPGATVAELCEHVPGPDFPTEAEIITPRADLQKVYE**

consensus/70% **PAVLPARLPNLLLNGTTGIAVGMATDVPPHNLREVASACVRLLDQPGATVAELCEHVPGPDFPTEAEIITPRADLQKVYE**

cov pid **241**  **: . . . . 3 . .** **320**

1 DFU7 100.0% 100.0% **TGRGSVRMRAVYRVEDGDIVIHALPHQVSGSKVLEQIAGQMQAKKLPMVADLRDESDHENPTRIVIIPRSNRVDVEELMT**

2 DFU9 100.0% 99.9% **TGRGSVRMRAVYRVEDGDIVIHALPHQVSGSKVLEQIAGQMQAKKLPMVADLRDESDHENPTRIVIIPRSNRVDVEELMT**

3 DFU16 100.0% 99.9% **TGRGSVRMRAVYRVEDGDIVIHALPHQVSGSKVLEQIAGQMQAKKLPMVADLRDESDHENPTRIVIIPRSNRVDVEELMT**

4 DFU48 100.0% 100.0% **TGRGSVRMRAVYRVEDGDIVIHALPHQVSGSKVLEQIAGQMQAKKLPMVADLRDESDHENPTRIVIIPRSNRVDVEELMT**

5 DFU58 100.0% 99.9% **TGRGSVRMRAVYRVEDGDIVIQALPHQVSGSKVLEQIAGQMQAKKLPMVADLRDESDHENPTRIVIIPRSNRVDVEELMT**

6 PAO1 100.0% 99.9% **TGRGSVRMRAVYRVEDGDIVIHALPHQVSGSKVLEQIAGQMQAKKLPMVADLRDESDHENPTRIVIIPRSNRVDVEELMT**

7 ATCC33363 100.0% 99.7% **TGRGSVRMRAVYRVEDGDIVIQALPHQVSGSKVLEQIAGQMQAKKLPMVADLRDESDHENPTRIVIIPRSNRVDVEELMT**

8 ZBX-P25 100.0% 99.9% **TGRGSVRMRAVYRVEDGDIVIQALPHQVSGSKVLEQIAGQMQAKKLPMVADLRDESDHENPTRIVIIPRSNRVDVEELMT**

9 DMC-20C 100.0% 99.9% **TGRGSVRMRAVYRVEDGDIVIQALPHQVSGSKVLEQIAGQMQAKKLPMVADLRDESDHENPTRIVIIPRSNRVDVEELMT**

10 P8W 100.0% 99.9% **TGRGSVRMRAVYRVEDGDIVIQALPHQVSGSKVLEQIAGQMQAKKLPMVADLRDESDHENPTRIVIIPRSNRVDVEELMT**

11 UTDF19 100.0% 99.7% **TGRGSVRMRAVYRVEDGDIVIQALPHQVSGSKVLEQIAGQMQAKKLPMVADLRDESDHENPTRIVIIPRSNRVDVEELMT**

12 CMPL223 100.0% 99.9% **TGRGSVRMRAVYRVEDGDIVIQALPHQVSGSKVLEQIAGQMQAKKLPMVADLRDESDHENPTRIVIIPRSNRVDVEELMT**

13 AS_23 100.0% 100.0% **TGRGSVRMRAVYRVEDGDIVIHALPHQVSGSKVLEQIAGQMQAKKLPMVADLRDESDHENPTRIVIIPRSNRVDVEELMT**

14 PS1972 100.0% 99.7% **TGRGSVRMRAVYRVEDGDIVIQALPHQVSGSKVLEQIAGQMQAKKLPMVADLRDESDHENPTRIVIIPRSNRVDVEELMT**

15 BA10747 100.0% 100.0% **TGRGSVRMRAVYRVEDGDIVIHALPHQVSGSKVLEQIAGQMQAKKLPMVADLRDESDHENPTRIVIIPRSNRVDVEELMT**

16 ST773 100.0% 100.0% **TGRGSVRMRAVYRVEDGDIVIHALPHQVSGSKVLEQIAGQMQAKKLPMVADLRDESDHENPTRIVIIPRSNRVDVEELMT**

17 DSM50071 100.0% 99.7% **TGRGSVRMRAVYRVEDGDIVIHALPHQVSGSKVLEQIAGQMQAKKLPMVADLRDESDHENPTRIVIIPRSNRVDVEELMT**

consensus/100% **TGRGSVRMRAVYRVEDGDIVIpALPHQVSGSKVLEQIAGQMQAKKLPMVADLRDESDHENPTRIVIIPRSNRVDVEELMT**

consensus/90% **TGRGSVRMRAVYRVEDGDIVIpALPHQVSGSKVLEQIAGQMQAKKLPMVADLRDESDHENPTRIVIIPRSNRVDVEELMT**

consensus/80% **TGRGSVRMRAVYRVEDGDIVIpALPHQVSGSKVLEQIAGQMQAKKLPMVADLRDESDHENPTRIVIIPRSNRVDVEELMT**

consensus/70% **TGRGSVRMRAVYRVEDGDIVIpALPHQVSGSKVLEQIAGQMQAKKLPMVADLRDESDHENPTRIVIIPRSNRVDVEELMT**

cov pid **321**  **. . : . . . . 4** **400**

1 DFU7 100.0% 100.0% **HLFATTDLETSYRVNLNIIGLDGKPQVKDLRQLLSEWLQFRIGTVRRRLQFRLDKVERRLHLLDGLLIAFLNLDEVIHII**

2 DFU9 100.0% 99.9% **HLFATTDLETSYRVNLNIIGLDGKPQVKDLRQLLSEWLQFRIGTVRRRLQFRLDKVERRLHLLDGLLIAFLNLDEVIHII**

3 DFU16 100.0% 99.9% **HLFATTDLETSYRVNLNIIGLDGKPQVKDLRQLLSEWLQFRIGTVRRRLQFRLDKVERRLHLLDGLLIAFLNLDEVIHII**

4 DFU48 100.0% 100.0% **HLFATTDLETSYRVNLNIIGLDGKPQVKDLRQLLSEWLQFRIGTVRRRLQFRLDKVERRLHLLDGLLIAFLNLDEVIHII**

5 DFU58 100.0% 99.9% **HLFATTDLETSYRVNLNIIGLDGKPQVKDLRQLLSEWLQFRIGTVRRRLQFRLDKVERRLHLLDGLLIAFLNLDEVIHII**

6 PAO1 100.0% 99.9% **HLFATTDLETSYRVNLNIIGLDGKPQVKDLRQLLSEWLQFRIGTVRRRLQFRLDKVERRLHLLDGLLIAFLNLDEVIHII**

7 ATCC33363 100.0% 99.7% **HLFATTDLETSYRVNLNIIGLDGKPQVKDLRQLLSEWLQFRIGTVRRRLQFRLDKVERRLHLLDGLLIAFLNLDEVIHII**

8 ZBX-P25 100.0% 99.9% **HLFATTDLETSYRVNLNIIGLDGKPQVKDLRQLLSEWLQFRIGTVRRRLQFRLDKVERRLHLLDGLLIAFLNLDEVIHII**

9 DMC-20C 100.0% 99.9% **HLFATTDLETSYRVNLNIIGLDGKPQVKDLRQLLSEWLQFRIGTVRRRLQFRLDKVERRLHLLDGLLIAFLNLDEVIHII**

10 P8W 100.0% 99.9% **HLFATTDLETSYRVNLNIIGLDGKPQVKDLRQLLSEWLQFRIGTVRRRLQFRLDKVERRLHLLDGLLIAFLNLDEVIHII**

11 UTDF19 100.0% 99.7% **HLFATTDLETSYRVNLNIIGLDGKPQVKDLRQLLSEWLQFRIGTVRRRLQFRLDKVERRLHLLDGLLIAFLNLDEVIHII**

12 CMPL223 100.0% 99.9% **HLFATTDLETSYRVNLNIIGLDGKPQVKDLRQLLSEWLQFRIGTVRRRLQFRLDKVERRLHLLDGLLIAFLNLDEVIHII**

13 AS_23 100.0% 100.0% **HLFATTDLETSYRVNLNIIGLDGKPQVKDLRQLLSEWLQFRIGTVRRRLQFRLDKVERRLHLLDGLLIAFLNLDEVIHII**

14 PS1972 100.0% 99.7% **HLFATTDLETSYRVNLNIIGLDGKPQVKDLRQLLSEWLQFRIGTVRRRLQFRLDKVERRLHLLDGLLIAFLNLDEVIHII**

15 BA10747 100.0% 100.0% **HLFATTDLETSYRVNLNIIGLDGKPQVKDLRQLLSEWLQFRIGTVRRRLQFRLDKVERRLHLLDGLLIAFLNLDEVIHII**

16 ST773 100.0% 100.0% **HLFATTDLETSYRVNLNIIGLDGKPQVKDLRQLLSEWLQFRIGTVRRRLQFRLDKVERRLHLLDGLLIAFLNLDEVIHII**

17 DSM50071 100.0% 99.7% **HLFATTDLETSYRVNLNIIGLDGKPQVKDLRQLLSEWLQFRIGTVRRRLQFRLDKVERRLHLLDGLLIAFLNLDEVIHII**

consensus/100% **HLFATTDLETSYRVNLNIIGLDGKPQVKDLRQLLSEWLQFRIGTVRRRLQFRLDKVERRLHLLDGLLIAFLNLDEVIHII**

consensus/90% **HLFATTDLETSYRVNLNIIGLDGKPQVKDLRQLLSEWLQFRIGTVRRRLQFRLDKVERRLHLLDGLLIAFLNLDEVIHII**

consensus/80% **HLFATTDLETSYRVNLNIIGLDGKPQVKDLRQLLSEWLQFRIGTVRRRLQFRLDKVERRLHLLDGLLIAFLNLDEVIHII**

consensus/70% **HLFATTDLETSYRVNLNIIGLDGKPQVKDLRQLLSEWLQFRIGTVRRRLQFRLDKVERRLHLLDGLLIAFLNLDEVIHII**

cov pid **401**  **. . . . : . . .** **480**

1 DFU7 100.0% 100.0% **RTEDQPKAVLMERFELSEVQADYILDTRLRQLARLEEMKIRGEQEELLKEQKRLQTLLGSEAKLKKLVREELIKDAETYG**

2 DFU9 100.0% 99.9% **RTEDQPKAVLMERFELSEVQADYILDTRLRQLARLEEMKIRGEQEELLKEQKRLQTLLGSEAKLKKLVREELIKDAETYG**

3 DFU16 100.0% 99.9% **RTEDQPKAVLMERFELSEVQADYILDTRLRQLARLEEMKIRGEQEELLKEQKRLQTLLGSEAKLKKLVREELIKDAETYG**

4 DFU48 100.0% 100.0% **RTEDQPKAVLMERFELSEVQADYILDTRLRQLARLEEMKIRGEQEELLKEQKRLQTLLGSEAKLKKLVREELIKDAETYG**

5 DFU58 100.0% 99.9% **RTEDQPKAVLMERFELSEVQADYILDTRLRQLARLEEMKIRGEQEELLKEQKRLQTLLGSEAKLKKLVREELIKDAETYG**

6 PAO1 100.0% 99.9% **RTEDQPKAVLMERFELSEVQADYILDTRLRQLARLEEMKIRGEQEELLKEQKRLQTLLGSEAKLKKLVREELIKDAETYG**

7 ATCC33363 100.0% 99.7% **RTEDQPKAVLMERFELSEVQADYILDTRLRQLARLEEMKIRGEQEELLKEQKRLQTLLGSEAKLKKLVREELIKDAETYG**

8 ZBX-P25 100.0% 99.9% **RTEDQPKAVLMERFELSEVQADYILDTRLRQLARLEEMKIRGEQEELLKEQKRLQTLLGSEAKLKKLVREELIKDAETYG**

9 DMC-20C 100.0% 99.9% **RTEDQPKAVLMERFELSEVQADYILDTRLRQLARLEEMKIRGEQEELLKEQKRLQTLLGSEAKLKKLVREELIKDAETYG**

10 P8W 100.0% 99.9% **RTEDQPKAVLMERFELSEVQADYILDTRLRQLARLEEMKIRGEQEELLKEQKRLQTLLGSEAKLKKLVREELIKDAETYG**

11 UTDF19 100.0% 99.7% **RTEDQPKAVLMERFELSEVQADYILDTRLRQLARLEEMKIRGEQEELLKEQKRLQTLLGSEAKLKKLVREELIKDAETYG**

12 CMPL223 100.0% 99.9% **RTEDQPKAVLMERFELSEVQADYILDTRLRQLARLEEMKIRGEQEELLKEQKRLQTLLGSEAKLKKLVREELIKDAETYG**

13 AS_23 100.0% 100.0% **RTEDQPKAVLMERFELSEVQADYILDTRLRQLARLEEMKIRGEQEELLKEQKRLQTLLGSEAKLKKLVREELIKDAETYG**

14 PS1972 100.0% 99.7% **RTEDQPKAVLMERFELSEVQADYILDTRLRQLARLEEMKIRGEQEELLKEQKRLQTLLGSEAKLKKLVREELIKDAETYG**

15 BA10747 100.0% 100.0% **RTEDQPKAVLMERFELSEVQADYILDTRLRQLARLEEMKIRGEQEELLKEQKRLQTLLGSEAKLKKLVREELIKDAETYG**

16 ST773 100.0% 100.0% **RTEDQPKAVLMERFELSEVQADYILDTRLRQLARLEEMKIRGEQEELLKEQKRLQTLLGSEAKLKKLVREELIKDAETYG**

17 DSM50071 100.0% 99.7% **RTEDQPKAVLMERFELSEVQADYILDTRLRQLARLEEMKIRGEQEELLKEQKRLQTLLGSEAKLKKLVREELIKDAETYG**

consensus/100% **RTEDQPKAVLMERFELSEVQADYILDTRLRQLARLEEMKIRGEQEELLKEQKRLQTLLGSEAKLKKLVREELIKDAETYG**

consensus/90% **RTEDQPKAVLMERFELSEVQADYILDTRLRQLARLEEMKIRGEQEELLKEQKRLQTLLGSEAKLKKLVREELIKDAETYG**

consensus/80% **RTEDQPKAVLMERFELSEVQADYILDTRLRQLARLEEMKIRGEQEELLKEQKRLQTLLGSEAKLKKLVREELIKDAETYG**

consensus/70% **RTEDQPKAVLMERFELSEVQADYILDTRLRQLARLEEMKIRGEQEELLKEQKRLQTLLGSEAKLKKLVREELIKDAETYG**

cov pid **481**  **. 5 . . . . : .** **560**

1 DFU7 100.0% 100.0% **DDRRSPIVARAEARALSETELMPTEPVTVVLSEKGWVRCAKGHDIDAAGLSYKAGDGFKAAAPGRSNQYAVFIDSTGRSY**

2 DFU9 100.0% 99.9% **DDRRSPIVARAEARALSETELMPTEPVTVVLSEKGWVRCAKGHDIDAAGLSYKAGDGFKAAAPGRSNQYAVFIDSTGRSY**

3 DFU16 100.0% 99.9% **DDRRSPIVARAEARALSETELMPTEPVTVVLSEKGWVRCAKGHDIDAAGLSYKAGDGFKAAAPGRSNQYAVFIDSTGRSY**

4 DFU48 100.0% 100.0% **DDRRSPIVARAEARALSETELMPTEPVTVVLSEKGWVRCAKGHDIDAAGLSYKAGDGFKAAAPGRSNQYAVFIDSTGRSY**

5 DFU58 100.0% 99.9% **DDRRSPIVARAEARALSETELMPTEPVTVVLSEKGWVRCAKGHDIDAAGLSYKAGDGFKAAAPGRSNQYAVFIDSTGRSY**

6 PAO1 100.0% 99.9% **DDRRSPIVARAEARALSETELMPTEPVTVVLSEKGWVRCAKGHDIDAAGLSYKAGDGFKAAAPGRSNQYAVFIDSTGRSY**

7 ATCC33363 100.0% 99.7% **DDRRSPIVARAEARALSETELMPTEPVTVVLSEKGWVRCAKGHDIDAAGLSYKAGDGFKAAAPGRSNQYAVFIDSTGRSY**

8 ZBX-P25 100.0% 99.9% **DDRRSPIVARAEARALSETELMPTEPVTVVLSEKGWVRCAKGHDIDAAGLSYKAGDGFKAAAPGRSNQYAVFIDSTGRSY**

9 DMC-20C 100.0% 99.9% **DDRRSPIVARAEARALSETELMPTEPVTVVLSEKGWVRCAKGHDIDAAGLSYKAGDGFKAAAPGRSNQYAVFIDSTGRSY**

10 P8W 100.0% 99.9% **DDRRSPIVARAEARALSETELMPTEPVTVVLSEKGWVRCAKGHDIDAAGLSYKAGDGFKAAAPGRSNQYAVFIDSTGRSY**

11 UTDF19 100.0% 99.7% **DDRRSPIVARAEARALSETELMPTEPVTVVLSEKGWVRCAKGHDIDAAGLSYKAGDGFKAAAPGRSNQYAVFIDSTGRSY**

12 CMPL223 100.0% 99.9% **DDRRSPIVARAEARALSETELMPTEPVTVVLSEKGWVRCAKGHDIDAAGLSYKAGDGFKAAAPGRSNQYAVFIDSTGRSY**

13 AS_23 100.0% 100.0% **DDRRSPIVARAEARALSETELMPTEPVTVVLSEKGWVRCAKGHDIDAAGLSYKAGDGFKAAAPGRSNQYAVFIDSTGRSY**

14 PS1972 100.0% 99.7% **DDRRSPIVARAEARALSETELMPTEPVTVVLSEKGWVRCAKGHDIDAAGLSYKAGDGFKAAAPGRSNQYAVFIDSTGRSY**

15 BA10747 100.0% 100.0% **DDRRSPIVARAEARALSETELMPTEPVTVVLSEKGWVRCAKGHDIDAAGLSYKAGDGFKAAAPGRSNQYAVFIDSTGRSY**

16 ST773 100.0% 100.0% **DDRRSPIVARAEARALSETELMPTEPVTVVLSEKGWVRCAKGHDIDAAGLSYKAGDGFKAAAPGRSNQYAVFIDSTGRSY**

17 DSM50071 100.0% 99.7% **DDRRSPIVARAEARALSETELMPTEPVTVVLSEKGWVRCAKGHDIDAAGLSYKAGDGFKAAAPGRSNQYAVFIDSTGRSY**

consensus/100% **DDRRSPIVARAEARALSETELMPTEPVTVVLSEKGWVRCAKGHDIDAAGLSYKAGDGFKAAAPGRSNQYAVFIDSTGRSY**

consensus/90% **DDRRSPIVARAEARALSETELMPTEPVTVVLSEKGWVRCAKGHDIDAAGLSYKAGDGFKAAAPGRSNQYAVFIDSTGRSY**

consensus/80% **DDRRSPIVARAEARALSETELMPTEPVTVVLSEKGWVRCAKGHDIDAAGLSYKAGDGFKAAAPGRSNQYAVFIDSTGRSY**

consensus/70% **DDRRSPIVARAEARALSETELMPTEPVTVVLSEKGWVRCAKGHDIDAAGLSYKAGDGFKAAAPGRSNQYAVFIDSTGRSY**

cov pid **561**  **. . . 6 . . . .** **640**

1 DFU7 100.0% 100.0% **SLPAHSLPSARGQGEPLSGRLTPPPGASFECVLLPDDDALFVIASDAGYGFVVKGEDLQAKNKAGKALLSLPNGSAVVAP**

2 DFU9 100.0% 99.9% **SLPAHSLPSARGQGEPLSGRLTPPPGASFECVLLPDDDALFVIASDAGYGFVVKGEDLQAKNKAGKALLSLPNGSAVVAP**

3 DFU16 100.0% 99.9% **SLPAHSLPSARGQGEPLSGRLTPPPGASFECVLLPDDDALFVIASDAGYGFVVKGEDLQAKNKAGKALLSLPNGSAVVAP**

4 DFU48 100.0% 100.0% **SLPAHSLPSARGQGEPLSGRLTPPPGASFECVLLPDDDALFVIASDAGYGFVVKGEDLQAKNKAGKALLSLPNGSAVVAP**

5 DFU58 100.0% 99.9% **SLPAHSLPSARGQGEPLSGRLTPPPGASFECVLLPDDDALFVIASDAGYGFVVKGEDLQAKNKAGKALLSLPNGSAVVAP**

6 PAO1 100.0% 99.9% **SLPAHSLPSARGQGEPLSGRLTPPPGASFECVLLPDDDALFVIASDAGYGFVVKGEDLQAKNKAGKALLSLPNGSAVVAP**

7 ATCC33363 100.0% 99.7% **SLPAHSLPSARGQGEPLSGRLTPPPGASFECVLLPDDDALFVIASDAGYGFVVKGEDLQAKNKAGKALLSLPNGSAVVAP**

8 ZBX-P25 100.0% 99.9% **SLPAHSLPSARGQGEPLSGRLTPPPGASFECVLLPDDDALFVIASDAGYGFVVKGEDLQAKNKAGKALLSLPNGSAVVAP**

9 DMC-20C 100.0% 99.9% **SLPAHSLPSARGQGEPLSGRLTPPPGASFECVLLPDDDALFVIASDAGYGFVVKGEDLQAKNKAGKALLSLPNGSAVVAP**

10 P8W 100.0% 99.9% **SLPAHSLPSARGQGEPLSGRLTPPPGASFECVLLPDDDALFVIASDAGYGFVVKGEDLQAKNKAGKALLSLPNGSAVVAP**

11 UTDF19 100.0% 99.7% **SLPAHSLPSARGQGEPLSGRLTPPPGASFECVLLPDDDALFVIASDAGYGFVVKGEDLQAKNKAGKALLSLPNGSAVVAP**

12 CMPL223 100.0% 99.9% **SLPAHSLPSARGQGEPLSGRLTPPPGASFECVLLPDDDALFVIASDAGYGFVVKGEDLQAKNKAGKALLSLPNGSAVVAP**

13 AS_23 100.0% 100.0% **SLPAHSLPSARGQGEPLSGRLTPPPGASFECVLLPDDDALFVIASDAGYGFVVKGEDLQAKNKAGKALLSLPNGSAVVAP**

14 PS1972 100.0% 99.7% **SLPAHSLPSARGQGEPLSGRLTPPPGASFECVLLPDDDALFVIASDAGYGFVVKGEDLQAKNKAGKALLSLPNGSAVVAP**

15 BA10747 100.0% 100.0% **SLPAHSLPSARGQGEPLSGRLTPPPGASFECVLLPDDDALFVIASDAGYGFVVKGEDLQAKNKAGKALLSLPNGSAVVAP**

16 ST773 100.0% 100.0% **SLPAHSLPSARGQGEPLSGRLTPPPGASFECVLLPDDDALFVIASDAGYGFVVKGEDLQAKNKAGKALLSLPNGSAVVAP**

17 DSM50071 100.0% 99.7% **SLPAHSLPSARGQGEPLSGRLTPPPGASFECVLLPDDDALFVIASDAGYGFVVKGEDLQAKNKAGKALLSLPNGSAVVAP**

consensus/100% **SLPAHSLPSARGQGEPLSGRLTPPPGASFECVLLPDDDALFVIASDAGYGFVVKGEDLQAKNKAGKALLSLPNGSAVVAP**

consensus/90% **SLPAHSLPSARGQGEPLSGRLTPPPGASFECVLLPDDDALFVIASDAGYGFVVKGEDLQAKNKAGKALLSLPNGSAVVAP**

consensus/80% **SLPAHSLPSARGQGEPLSGRLTPPPGASFECVLLPDDDALFVIASDAGYGFVVKGEDLQAKNKAGKALLSLPNGSAVVAP**

consensus/70% **SLPAHSLPSARGQGEPLSGRLTPPPGASFECVLLPDDDALFVIASDAGYGFVVKGEDLQAKNKAGKALLSLPNGSAVVAP**

cov pid **641**  **: . . . . 7 . .** **720**

1 DFU7 100.0% 100.0% **RPVRDVEQDWLAAVTTEGRLLLFKVSDLPQLGKGKGNKIIGIPGERVASREEYLTDLAVLPAGATLVLQAGKRTLSLKGD**

2 DFU9 100.0% 99.9% **RPVRDVEQDWLAAVTTEGRLLLFKVSDLPQLGKGKGNKIIGIPGERVASREEYLTDLAVLPAGATLVLQAGKRTLSLKGD**

3 DFU16 100.0% 99.9% **RPVRDVEQDWLAAVTTEGRLLLFKVSDLPQLGKGKGNKIIGIPGERVASREEYLTDLAVLPAGATLVLQAGKRTLSLKGD**

4 DFU48 100.0% 100.0% **RPVRDVEQDWLAAVTTEGRLLLFKVSDLPQLGKGKGNKIIGIPGERVASREEYLTDLAVLPAGATLVLQAGKRTLSLKGD**

5 DFU58 100.0% 99.9% **RPVRDVEQDWLAAVTTEGRLLLFKVSDLPQLGKGKGNKIIGIPGERVASREEYLTDLAVLPAGATLVLQAGKRTLSLKGD**

6 PAO1 100.0% 99.9% **RPVRDVEQDWLAAVTTEGRLLLFKVSDLPQLGKGKGNKIIGIPGERVASREEYLTDLAVLPAGATLVLQAGKRTLSLKGD**

7 ATCC33363 100.0% 99.7% **RPVRDVEQDWLAAVTTEGRLLLFKVSDLPQLGKGKGNKIIGIPGERVASREEYLTDLAVLPAGATLVLQAGKRTLSLKGD**

8 ZBX-P25 100.0% 99.9% **RPVRDVEQDWLAAVTTEGRLLLFKVSDLPQLGKGKGNKIIGIPGERVASREEYLTDLAVLPAGATLVLQAGKRTLSLKGD**

9 DMC-20C 100.0% 99.9% **RPVRDVEQDWLAAVTTEGRLLLFKVSDLPQLGKGKGNKIIGIPGERVASREEYLTDLAVLPAGATLVLQAGKRTLSLKGD**

10 P8W 100.0% 99.9% **RPVRDVEQDWLAAVTTEGRLLLFKVSDLPQLGKGKGNKIIGIPGERVASREEYLTDLAVLPAGATLVLQAGKRTLSLKGD**

11 UTDF19 100.0% 99.7% **RPVRDVEQDWLAAVTTEGRLLLFKVSDLPQLGKGKGNKIIGIPGERVASREEYLTDLAVLPAGATLVLQAGKRTLSLKGD**

12 CMPL223 100.0% 99.9% **RPVRDVEQDWLAAVTTEGRLLLFKVSDLPQLGKGKGNKIIGIPGERVASREEYLTDLAVLPAGATLVLQAGKRTLSLKGD**

13 AS_23 100.0% 100.0% **RPVRDVEQDWLAAVTTEGRLLLFKVSDLPQLGKGKGNKIIGIPGERVASREEYLTDLAVLPAGATLVLQAGKRTLSLKGD**

14 PS1972 100.0% 99.7% **RPVRDVEQDWLAAVTTEGRLLLFKVSDLPQLGKGKGNKIIGIPGERVASREEYLTDLAVLPAGATLVLQAGKRTLSLKGD**

15 BA10747 100.0% 100.0% **RPVRDVEQDWLAAVTTEGRLLLFKVSDLPQLGKGKGNKIIGIPGERVASREEYLTDLAVLPAGATLVLQAGKRTLSLKGD**

16 ST773 100.0% 100.0% **RPVRDVEQDWLAAVTTEGRLLLFKVSDLPQLGKGKGNKIIGIPGERVASREEYLTDLAVLPAGATLVLQAGKRTLSLKGD**

17 DSM50071 100.0% 99.7% **RPVRDVEQDWLAAVTTEGRLLLFKVSDLPQLGKGKGNKIIGIPGERVVSREEYLTDLAVLPAGATLVLQAGKRTLSLKGD**

consensus/100% **RPVRDVEQDWLAAVTTEGRLLLFKVSDLPQLGKGKGNKIIGIPGERVsSREEYLTDLAVLPAGATLVLQAGKRTLSLKGD**

consensus/90% **RPVRDVEQDWLAAVTTEGRLLLFKVSDLPQLGKGKGNKIIGIPGERVASREEYLTDLAVLPAGATLVLQAGKRTLSLKGD**

consensus/80% **RPVRDVEQDWLAAVTTEGRLLLFKVSDLPQLGKGKGNKIIGIPGERVASREEYLTDLAVLPAGATLVLQAGKRTLSLKGD**

consensus/70% **RPVRDVEQDWLAAVTTEGRLLLFKVSDLPQLGKGKGNKIIGIPGERVASREEYLTDLAVLPAGATLVLQAGKRTLSLKGD**

cov pid **721**  **. . : ]** **754**

1 DFU7 100.0% 100.0% **DLEHYKGERGRRGNKLPRGFQRVDSLLVDIPPQD**

2 DFU9 100.0% 99.9% **DLEHYKGERGRRGNKLPRGFQRVDSLLVDIPPQD**

3 DFU16 100.0% 99.9% **DLEHYKGERGRRGNKLPRGFQRVDSLLVDIPPQD**

4 DFU48 100.0% 100.0% **DLEHYKGERGRRGNKLPRGFQRVDSLLVDIPPQD**

5 DFU58 100.0% 99.9% **DLEHYKGERGRRGNKLPRGFQRVDSLLVDIPPQD**

6 PAO1 100.0% 99.9% **DLEHYKGERGRRGNKLPRGFQRVDSLLVDIPPQD**

7 ATCC33363 100.0% 99.7% **DLEHYKGERGRRGNKLPRGFQRVDSLLVDIPPQD**

8 ZBX-P25 100.0% 99.9% **DLEHYKGERGRRGNKLPRGFQRVDSLLVDIPPQD**

9 DMC-20C 100.0% 99.9% **DLEHYKGERGRRGNKLPRGFQRVDSLLVDIPPQD**

10 P8W 100.0% 99.9% **DLEHYKGERGRRGNKLPRGFQRVDSLLVDIPPQD**

11 UTDF19 100.0% 99.7% **DLEHYKGERGRRGNKLPRGFQRVDSLLVDIPPQD**

12 CMPL223 100.0% 99.9% **DLEHYKGERGRRGNKLPRGFQRVDSLLVDIPPQD**

13 AS_23 100.0% 100.0% **DLEHYKGERGRRGNKLPRGFQRVDSLLVDIPPQD**

14 PS1972 100.0% 99.7% **DLEHYKGERGRRGNKLPRGFQRVDSLLVDIPPQD**

15 BA10747 100.0% 100.0% **DLEHYKGERGRRGNKLPRGFQRVDSLLVDIPPQD**

16 ST773 100.0% 100.0% **DLEHYKGERGRRGNKLPRGFQRVDSLLVDIPPQD**

17 DSM50071 100.0% 99.7% **DLEHYKGERGRRGNKLPRGFQRVDSLLVDIPPQD**

consensus/100% **DLEHYKGERGRRGNKLPRGFQRVDSLLVDIPPQD**

consensus/90% **DLEHYKGERGRRGNKLPRGFQRVDSLLVDIPPQD**

consensus/80% **DLEHYKGERGRRGNKLPRGFQRVDSLLVDIPPQD**

consensus/70% **DLEHYKGERGRRGNKLPRGFQRVDSLLVDIPPQD**

**Supplementary Figure 3.** Multiple sequence alignment of the predicted amino acid sequences of ParC carried by P. aeruginosa isolates and close genomes retrieved from the BV-BRC database compared to that P. aeruginosa strains (PAO1 and ATCC^®^33363). The alignment was visualized using MView version 1.63 hosted by the EMBL-EBI; cov, coverage; pid, percent identity.

cov pid  **1** **[ . . . . : . . .** **80**

1 DFU7 100.0% 100.0% **MRILLAEDDLLLGDGIRAGLRLEGDTVEWVTDGVAAENALVTDEFDLLVLDIGLPRRSGLDILRNLRHQGLLTPVLLLTA**

2 DFU9 100.0% 99.5% **MRILLAEDDLLLGDGIRAGLRLEGDTVEWVTDGVAAENALVTDEFDLLVLDIGLPRRSGLDILRNLRHQGRLTPVLLLTA**

3 DFU16 100.0% 99.5% **MRILLAEDDLLLGDGIRAGLRLEGDTVEWVTDGVAAENALVTDEFDLLVLDIGLPRRSGLDILRNLRHQGRLTPVLLLTA**

4 DFU48 100.0% 100.0% **MRILLAEDDLLLGDGIRAGLRLEGDTVEWVTDGVAAENALVTDEFDLLVLDIGLPRRSGLDILRNLRHQGLLTPVLLLTA**

5 DFU58 100.0% 99.5% **MRILLAEDDLLLGDGIRAGLRLEGDTVEWVTDGVAAENALVTDEFDLLVLDIGLPRRSGLDILRNLRHQGRLTPVLLLTA**

6 PAO1 100.0% 100.0% **MRILLAEDDLLLGDGIRAGLRLEGDTVEWVTDGVAAENALVTDEFDLLVLDIGLPRRSGLDILRNLRHQGLLTPVLLLTA**

7 ATCC33363 100.0% 99.5% **MRILLAEDDLLLGDGIRAGLRLEGDTVEWVTDGVAAENALVTDEFDLLVLDIGLPRRSGLDILRNLRHQGRLTPVLLLTA**

8 ZBX-P25 100.0% 99.5% **MRILLAEDDLLLGDGIRAGLRLEGDTVEWVTDGVAAENALVTDEFDLLVLDIGLPRRSGLDILRNLRHQGRLTPVLLLTA**

9 DMC-20C 100.0% 99.5% **MRILLAEDDLLLGDGIRAGLRLEGDTVEWVTDGVAAENALVTDEFDLLVLDIGLPRRSGLDILRNLRHQGRLTPVLLLTA**

10 P8W 100.0% 99.5% **MRILLAEDDLLLGDGIRAGLRLEGDTVEWVTDGVAAENALVTDEFDLLVLDIGLPRRSGLDILRNLRHQGRLTPVLLLTA**

11 UTDF19-28A 100.0% 99.5% **MRILLAEDDLLLGDGIRAGLRLEGDTVEWVTDGVAAENALVTDEFDLLVLDIGLPRRSGLDILRNLRHQGRLTPVLLLTA**

12 CMPL223 100.0% 99.5% **MRILLAEDDLLLGDGIRAGLRLEGDTVEWVTDGVAAENALVTDEFDLLVLDIGLPRRSGLDILRNLRHQGRLTPVLLLTA**

13 AS_23 100.0% 100.0% **MRILLAEDDLLLGDGIRAGLRLEGDTVEWVTDGVAAENALVTDEFDLLVLDIGLPRRSGLDILRNLRHQGLLTPVLLLTA**

14 PS1972 100.0% 99.5% **MRILLAEDDLLLGDGIRAGLRLEGDTVEWVTDGVAAENALVTDEFDLLVLDIGLPRRSGLDILRNLRHQGRLTPVLLLTA**

15 BA10747 100.0% 100.0% **MRILLAEDDLLLGDGIRAGLRLEGDTVEWVTDGVAAENALVTDEFDLLVLDIGLPRRSGLDILRNLRHQGLLTPVLLLTA**

16 ST773 100.0% 100.0% **MRILLAEDDLLLGDGIRAGLRLEGDTVEWVTDGVAAENALVTDEFDLLVLDIGLPRRSGLDILRNLRHQGLLTPVLLLTA**

17 DSM50071 100.0% 99.5% **MRILLAEDDLLLGDGIRAGLRLEGDTVEWVTDGVAAENALVTDEFDLLVLDIGLPRRSGLDILRNLRHQGRLTPVLLLTA**

consensus/100% **MRILLAEDDLLLGDGIRAGLRLEGDTVEWVTDGVAAENALVTDEFDLLVLDIGLPRRSGLDILRNLRHQGhLTPVLLLTA**

consensus/90% **MRILLAEDDLLLGDGIRAGLRLEGDTVEWVTDGVAAENALVTDEFDLLVLDIGLPRRSGLDILRNLRHQGhLTPVLLLTA**

consensus/80% **MRILLAEDDLLLGDGIRAGLRLEGDTVEWVTDGVAAENALVTDEFDLLVLDIGLPRRSGLDILRNLRHQGhLTPVLLLTA**

consensus/70% **MRILLAEDDLLLGDGIRAGLRLEGDTVEWVTDGVAAENALVTDEFDLLVLDIGLPRRSGLDILRNLRHQGhLTPVLLLTA**

cov pid  **81**  **. 1 . . . . : .** **160**

1 DFU7 100.0% 100.0% **RDKVADRVAGLDSGADDYLTKPFDLDELQARVRALTRRTTGRALPQLVHGELRLDPATHQVTLSGQAVELAPREYALLRL**

2 DFU9 100.0% 99.5% **RDKVADRVAGLDSGADDYLTKPFDLDELQARVRALTRRTTGRALPQLVHGELRLDPATHQVTLSGQAVELAPREYALLRL**

3 DFU16 100.0% 99.5% **RDKVADRVAGLDSGADDYLTKPFDLDELQARVRALTRRTTGRALPQLVHGELRLDPATHQVTLSGQAVELAPREYALLRL**

4 DFU48 100.0% 100.0% **RDKVADRVAGLDSGADDYLTKPFDLDELQARVRALTRRTTGRALPQLVHGELRLDPATHQVTLSGQAVELAPREYALLRL**

5 DFU58 100.0% 99.5% **RDKVADRVAGLDSGADDYLTKPFDLDELQARVRALTRRTTGRALPQLVHGELRLDPATHQVTLSGQAVELAPREYALLRL**

6 PAO1 100.0% 100.0% **RDKVADRVAGLDSGADDYLTKPFDLDELQARVRALTRRTTGRALPQLVHGELRLDPATHQVTLSGQAVELAPREYALLRL**

7 ATCC33363 100.0% 99.5% **RDKVADRVAGLDSGADDYLTKPFDLDELQARVRALTRRTTGRALPQLVHGELRLDPATHQVTLSGQAVELAPREYALLRL**

8 ZBX-P25 100.0% 99.5% **RDKVADRVAGLDSGADDYLTKPFDLDELQARVRALTRRTTGRALPQLVHGELRLDPATHQVTLSGQAVELAPREYALLRL**

9 DMC-20C 100.0% 99.5% **RDKVADRVAGLDSGADDYLTKPFDLDELQARVRALTRRTTGRALPQLVHGELRLDPATHQVTLSGQAVELAPREYALLRL**

10 P8W 100.0% 99.5% **RDKVADRVAGLDSGADDYLTKPFDLDELQARVRALTRRTTGRALPQLVHGELRLDPATHQVTLSGQAVELAPREYALLRL**

11 UTDF19-28A 100.0% 99.5% **RDKVADRVAGLDSGADDYLTKPFDLDELQARVRALTRRTTGRALPQLVHGELRLDPATHQVTLSGQAVELAPREYALLRL**

12 CMPL223 100.0% 99.5% **RDKVADRVAGLDSGADDYLTKPFDLDELQARVRALTRRTTGRALPQLVHGELRLDPATHQVTLSGQAVELAPREYALLRL**

13 AS_23 100.0% 100.0% **RDKVADRVAGLDSGADDYLTKPFDLDELQARVRALTRRTTGRALPQLVHGELRLDPATHQVTLSGQAVELAPREYALLRL**

14 PS1972 100.0% 99.5% **RDKVADRVAGLDSGADDYLTKPFDLDELQARVRALTRRTTGRALPQLVHGELRLDPATHQVTLSGQAVELAPREYALLRL**

15 BA10747 100.0% 100.0% **RDKVADRVAGLDSGADDYLTKPFDLDELQARVRALTRRTTGRALPQLVHGELRLDPATHQVTLSGQAVELAPREYALLRL**

16 ST773 100.0% 100.0% **RDKVADRVAGLDSGADDYLTKPFDLDELQARVRALTRRTTGRALPQLVHGELRLDPATHQVTLSGQAVELAPREYALLRL**

17 DSM50071 100.0% 99.5% **RDKVADRVAGLDSGADDYLTKPFDLDELQARVRALTRRTTGRALPQLVHGELRLDPATHQVTLSGQAVELAPREYALLRL**

consensus/100% **RDKVADRVAGLDSGADDYLTKPFDLDELQARVRALTRRTTGRALPQLVHGELRLDPATHQVTLSGQAVELAPREYALLRL**

consensus/90% **RDKVADRVAGLDSGADDYLTKPFDLDELQARVRALTRRTTGRALPQLVHGELRLDPATHQVTLSGQAVELAPREYALLRL**

consensus/80% **RDKVADRVAGLDSGADDYLTKPFDLDELQARVRALTRRTTGRALPQLVHGELRLDPATHQVTLSGQAVELAPREYALLRL**

consensus/70% **RDKVADRVAGLDSGADDYLTKPFDLDELQARVRALTRRTTGRALPQLVHGELRLDPATHQVTLSGQAVELAPREYALLRL**

cov pid **161**  **. . . 2 . .]** **221**

1 DFU7 100.0% 100.0% **LLENSGKVLSRNQLEQSLYGWSGDVESNAIEVHVHHLRRKLGNQLIRTVRGIGYGIDQPAP**

2 DFU9 100.0% 99.5% **LLENSGKVLSRNQLEQSLYGWSGDVESNAIEVHVHHLRRKLGNQLIRTVRGIGYGIDQPAP**

3 DFU16 100.0% 99.5% **LLENSGKVLSRNQLEQSLYGWSGDVESNAIEVHVHHLRRKLGNQLIRTVRGIGYGIDQPAP**

4 DFU48 100.0% 100.0% **LLENSGKVLSRNQLEQSLYGWSGDVESNAIEVHVHHLRRKLGNQLIRTVRGIGYGIDQPAP**

5 DFU58 100.0% 99.5% **LLENSGKVLSRNQLEQSLYGWSGDVESNAIEVHVHHLRRKLGNQLIRTVRGIGYGIDQPAP**

6 PAO1 100.0% 100.0% **LLENSGKVLSRNQLEQSLYGWSGDVESNAIEVHVHHLRRKLGNQLIRTVRGIGYGIDQPAP**

7 ATCC33363 100.0% 99.5% **LLENSGKVLSRNQLEQSLYGWSGDVESNAIEVHVHHLRRKLGNQLIRTVRGIGYGIDQPAP**

8 ZBX-P25 100.0% 99.5% **LLENSGKVLSRNQLEQSLYGWSGDVESNAIEVHVHHLRRKLGNQLIRTVRGIGYGIDQPAP**

9 DMC-20C 100.0% 99.5% **LLENSGKVLSRNQLEQSLYGWSGDVESNAIEVHVHHLRRKLGNQLIRTVRGIGYGIDQPAP**

10 P8W 100.0% 99.5% **LLENSGKVLSRNQLEQSLYGWSGDVESNAIEVHVHHLRRKLGNQLIRTVRGIGYGIDQPAP**

11 UTDF19-28A 100.0% 99.5% **LLENSGKVLSRNQLEQSLYGWSGDVESNAIEVHVHHLRRKLGNQLIRTVRGIGYGIDQPAP**

12 CMPL223 100.0% 99.5% **LLENSGKVLSRNQLEQSLYGWSGDVESNAIEVHVHHLRRKLGNQLIRTVRGIGYGIDQPAP**

13 AS_23 100.0% 100.0% **LLENSGKVLSRNQLEQSLYGWSGDVESNAIEVHVHHLRRKLGNQLIRTVRGIGYGIDQPAP**

14 PS1972 100.0% 99.5% **LLENSGKVLSRNQLEQSLYGWSGDVESNAIEVHVHHLRRKLGNQLIRTVRGIGYGIDQPAP**

15 BA10747 100.0% 100.0% **LLENSGKVLSRNQLEQSLYGWSGDVESNAIEVHVHHLRRKLGNQLIRTVRGIGYGIDQPAP**

16 ST773 100.0% 100.0% **LLENSGKVLSRNQLEQSLYGWSGDVESNAIEVHVHHLRRKLGNQLIRTVRGIGYGIDQPAP**

17 DSM50071 100.0% 99.5% **LLENSGKVLSRNQLEQSLYGWSGDVESNAIEVHVHHLRRKLGNQLIRTVRGIGYGIDQPAP**

consensus/100% **LLENSGKVLSRNQLEQSLYGWSGDVESNAIEVHVHHLRRKLGNQLIRTVRGIGYGIDQPAP**

consensus/90% **LLENSGKVLSRNQLEQSLYGWSGDVESNAIEVHVHHLRRKLGNQLIRTVRGIGYGIDQPAP**

consensus/80% **LLENSGKVLSRNQLEQSLYGWSGDVESNAIEVHVHHLRRKLGNQLIRTVRGIGYGIDQPAP**

consensus/70% **LLENSGKVLSRNQLEQSLYGWSGDVESNAIEVHVHHLRRKLGNQLIRTVRGIGYGIDQPAP**

**Supplementary Figure 4.** Multiple sequence alignment of the predicted amino acid sequences of PmrA (BasR) carried by P. aeruginosa isolates and close genomes retrieved from the BV-BRC database compared to that P. aeruginosa strains (PAO1 and ATCC^®^33363). The alignment was visualized using MView version 1.63 hosted by the EMBL-EBI; cov, coverage; pid, percent identity.

cov pid  **1** **[ . . . . : . . .** **80**

1 DFU7 100.0% 100.0% **MPRTAVPSVRRRLLINLLVGFVLCWLSVAALTYHLSLKQVNRLFDDDMVDFGEAALRLLDLATEDQASEDGSITEIIERS**

2 DFU9 100.0% 99.0% **MSRAAVPSVRRRLLVNLLVGFVLCWLSVAALTYHLSLKQVNRLFDDDMVDFGEAALRLLDLATEDQAGEDGSITEIIERS**

3 DFU16 100.0% 99.0% **MSRAAVPSVRRRLLVNLLVGFVLCWLSVAALTYHLSLKQVNRLFDDDMVDFGEAALRLLDLATEDQAGEDGSITEIIERS**

4 DFU48 100.0% 100.0% **MPRTAVPSVRRRLLINLLVGFVLCWLSVAALTYHLSLKQVNRLFDDDMVDFGEAALRLLDLATEDQASEDGSITEIIERS**

5 DFU58 100.0% 99.2% **MSRAAVPSVRRRLLVNLLVGFVLCWLSVAALTYHLSLKQVNRLFDDDMVDFGEAALRLLDLATEDQAGEDGSITEIIERS**

6 PAO1 100.0% 99.0% **MSRAAVPSVRRRLLVNLLVGFVLCWLSVAALTYHLSLKQVNRLFDDDMVDFGEAALRLLDLATEDQAGEDGSITEIIERS**

7 ATCC33363 100.0% 99.2% **MSRAAVPSVRRRLLVNLLVGFVLCWLSVAALTYHLSLKQVNRLFDDDMVDFGEAALRLLDLATEDQAGEDGSITEIIERS**

8 ZBX-P25 100.0% 99.2% **MSRAAVPSVRRRLLVNLLVGFVLCWLSVAALTYHLSLKQVNRLFDDDMVDFGEAALRLLDLATEDQAGEDGSITEIIERS**

9 DMC-20C 100.0% 99.2% **MSRAAVPSVRRRLLVNLLVGFVLCWLSVAALTYHLSLKQVNRLFDDDMVDFGEAALRLLDLATEDQAGEDGSITEIIERS**

10 P8W 100.0% 99.2% **MSRAAVPSVRRRLLVNLLVGFVLCWLSVAALTYHLSLKQVNRLFDDDMVDFGEAALRLLDLATEDQAGEDGSITEIIERS**

11 UTDF19-28A 100.0% 99.0% **MSRAAVPSVRRRLLVNLLVGFVLCWLSVAALTYHLSLKQVNRLFDDDMVDFGEAALRLLDLATEDQAGEDGSITEIIERS**

12 CMPL223 100.0% 99.0% **MSRAAVPSVRRRLLVNLLVGFVLCWLSVAALTYHLSLKQVNRLFDDDMVDFGEAALRLLDLATEDQAGEDGSITEIIERS**

13 AS_23 100.0% 100.0% **MPRTAVPSVRRRLLINLLVGFVLCWLSVAALTYHLSLKQVNRLFDDDMVDFGEAALRLLDLATEDQASEDGSITEIIERS**

14 PS1972 100.0% 99.2% **MSRAAVPSVRRRLLVNLLVGFVLCWLSVAALTYHLSLKQVNRLFDDDMVDFGEAALRLLDLATEDQAGEDGSITEIIERS**

15 BA10747 100.0% 100.0% **MPRTAVPSVRRRLLINLLVGFVLCWLSVAALTYHLSLKQVNRLFDDDMVDFGEAALRLLDLATEDQASEDGSITEIIERS**

16 ST773 100.0% 99.6% **MPRTAVPSVRRRLLINLLVGFVLCWLSVAALTYHLSLKQVNRLFDDDMVDFSEAALRLLDLATEDQASEDGSITEIIERS**

17 DSM50071 100.0% 99.2% **MSRAAVPSVRRRLLVNLLVGFVLCWLSVAALTYHLSLKQVNRLFDDDMVDFGEAALRLLDLATEDQAGEDGSITEIIERS**

18 XDR-PA 100.0% 99.2% **MSRAAVPSVRRRLLVNLLVGFVLCWLSVAALTYHLSLKQVNRLFDDDMVDFGEAALRLLDLATEDQAGEDGSITEIIERS**

consensus/100% **MsRsAVPSVRRRLLlNLLVGFVLCWLSVAALTYHLSLKQVNRLFDDDMVDFuEAALRLLDLATEDQAuEDGSITEIIERS**

consensus/90% **MsRsAVPSVRRRLLlNLLVGFVLCWLSVAALTYHLSLKQVNRLFDDDMVDFGEAALRLLDLATEDQAuEDGSITEIIERS**

consensus/80% **MsRsAVPSVRRRLLlNLLVGFVLCWLSVAALTYHLSLKQVNRLFDDDMVDFGEAALRLLDLATEDQAuEDGSITEIIERS**

consensus/70% **MSRAAVPSVRRRLLVNLLVGFVLCWLSVAALTYHLSLKQVNRLFDDDMVDFGEAALRLLDLATEDQAGEDGSITEIIERS**

cov pid  **81**  **. 1 . . . . : .** **160**

1 DFU7 100.0% 100.0% **REAIQGLPLLRRESALGYALWRDGQPLLSSLNLPPEITAQGPGFSTVEAQGTHWRVLQLNIDGFQIWISENLIYRQHTMN**

2 DFU9 100.0% 99.0% **REAIQGLPLLRRESALGYALWRDGQPLLSSLNLPPEITAQGPGFSTVEAQGTHWRVLQLNIDGFQIWISENLIYRQHTMN**

3 DFU16 100.0% 99.0% **REAIQGLPLLRRESALGYALWRDGQPLLSSLNLPPEITAQGPGFSTVEAQGTHWRVLQLNIDGFQIWISENLIYRQHTMN**

4 DFU48 100.0% 100.0% **REAIQGLPLLRRESALGYALWRDGQPLLSSLNLPPEITAQGPGFSTVEAQGTHWRVLQLNIDGFQIWISENLIYRQHTMN**

5 DFU58 100.0% 99.2% **REAIQGLPLLRRESALGYALWRDGQPLLSSLNLPPEITAQGPGFSTVEAQGTHWRVLQLNIDGFQIWISENLIYRQHTMN**

6 PAO1 100.0% 99.0% **REAIQGLPLLRRESALGYALWRDGQPLLSSLNLPPEITAQGPGFSTVEAQGTHWRVLQLNIDGFQIWISENLIYRQHTMN**

7 ATCC33363 100.0% 99.2% **REAIQGLPLLRRESALGYALWRDGQPLLSSLNLPPEITAQGPGFSTVEAQGTHWRVLQLNIDGFQIWISENLIYRQHTMN**

8 ZBX-P25 100.0% 99.2% **REAIQGLPLLRRESALGYALWRDGQPLLSSLNLPPEITAQGPGFSTVEAQGTHWRVLQLNIDGFQIWISENLIYRQHTMN**

9 DMC-20C 100.0% 99.2% **REAIQGLPLLRRESALGYALWRDGQPLLSSLNLPPEITAQGPGFSTVEAQGTHWRVLQLNIDGFQIWISENLIYRQHTMN**

10 P8W 100.0% 99.2% **REAIQGLPLLRRESALGYALWRDGQPLLSSLNLPPEITAQGPGFSTVEAQGTHWRVLQLNIDGFQIWISENLIYRQHTMN**

11 UTDF19-28A 100.0% 99.0% **REAIQGLPLLRRESALGYALWRDGQPLLSSLNLPPEITAQGPGFSTVEAQGTHWRVLQLNIDGFQIWISENLIYRQHTMN**

12 CMPL223 100.0% 99.0% **REAIQGLPLLRRESALGYALWCDGQPLLSSLNLPPEITAQGPGFSTVEAQGTHWRVLQLNIDGFQIWISENLIYRQHTMN**

13 AS_23 100.0% 100.0% **REAIQGLPLLRRESALGYALWRDGQPLLSSLNLPPEITAQGPGFSTVEAQGTHWRVLQLNIDGFQIWISENLIYRQHTMN**

14 PS1972 100.0% 99.2% **REAIQGLPLLRRESALGYALWRDGQPLLSSLNLPPEITAQGPGFSTVEAQGTHWRVLQLNIDGFQIWISENLIYRQHTMN**

15 BA10747 100.0% 100.0% **REAIQGLPLLRRESALGYALWRDGQPLLSSLNLPPEITAQGPGFSTVEAQGTHWRVLQLNIDGFQIWISENLIYRQHTMN**

16 ST773 100.0% 99.6% **REAIQGLPLLRRESALGYALWRDGQPLLSSLNLPPEITAQGPGFSTVEAQGTHWRVLQLNIDGFQIWISENLIYRQHTMN**

17 DSM50071 100.0% 99.2% **REAIQGLPLLRRESALGYALWRDGQPLLSSLNLPPEITAQGPGFSTVEAQGTHWRVLQLNIDGFQIWISENLIYRQHTMN**

18 XDR-PA 100.0% 99.2% **REAIQGLPLLRRESALGYALWRDGQPLLSSLNLPPEITAQGPGFSTVEAQGTHWRVLQLNIDGFQIWISENLIYRQHTMN**

consensus/100% **REAIQGLPLLRRESALGYALWpDGQPLLSSLNLPPEITAQGPGFSTVEAQGTHWRVLQLNIDGFQIWISENLIYRQHTMN**

consensus/90% **REAIQGLPLLRRESALGYALWRDGQPLLSSLNLPPEITAQGPGFSTVEAQGTHWRVLQLNIDGFQIWISENLIYRQHTMN**

consensus/80% **REAIQGLPLLRRESALGYALWRDGQPLLSSLNLPPEITAQGPGFSTVEAQGTHWRVLQLNIDGFQIWISENLIYRQHTMN**

consensus/70% **REAIQGLPLLRRESALGYALWRDGQPLLSSLNLPPEITAQGPGFSTVEAQGTHWRVLQLNIDGFQIWISENLIYRQHTMN**

cov pid **161**  **. . . 2 . . . .** **240**

1 DFU7 100.0% 100.0% **LLLFYSLFPLLLALPLLGGLVWFGVARGLAPLREVQAEVQQRSARHLQPIAVEAVPLEIRGLIDELNLLLERLRTALEAE**

2 DFU9 100.0% 99.0% **LLLFYSLFPLLLALPLLGGLVWFGVARGLAPLREVQAEVQQRSARHLQPIAVEAVPLEIRGLIDELNLLLERLRTALEAE**

3 DFU16 100.0% 99.0% **LLLFYSLFPLLLALPLLGGLVWFGVARGLAPLREVQAEVQQRSARHLQPIAVEAVPLEIRGLIDELNLLLERLRTALEAE**

4 DFU48 100.0% 100.0% **LLLFYSLFPLLLALPLLGGLVWFGVARGLAPLREVQAEVQQRSARHLQPIAVEAVPLEIRGLIDELNLLLERLRTALEAE**

5 DFU58 100.0% 99.2% **LLLFYSLFPLLLALPLLGGLVWFGVARGLAPLREVQAEVQQRSARHLQPIAVEAVPLEIRGLIDELNLLLERLRTALEAE**

6 PAO1 100.0% 99.0% **LLLFYSLFPLLLALPLLGGLVWFGVARGLAPLREVQAEVQQRSARHLQPIAVEAVPLEIRGLIDELNLLLERLRTALEAE**

7 ATCC33363 100.0% 99.2% **LLLFYSLFPLLLALPLLGGLVWFGVARGLAPLREVQAEVQQRSARHLQPIAVEAVPLEIRGLIDELNLLLERLRTALEAE**

8 ZBX-P25 100.0% 99.2% **LLLFYSLFPLLLALPLLGGLVWFGVARGLAPLREVQAEVQQRSARHLQPIAVEAVPLEIRGLIDELNLLLERLRTALEAE**

9 DMC-20C 100.0% 99.2% **LLLFYSLFPLLLALPLLGGLVWFGVARGLAPLREVQAEVQQRSARHLQPIAVEAVPLEIRGLIDELNLLLERLRTALEAE**

10 P8W 100.0% 99.2% **LLLFYSLFPLLLALPLLGGLVWFGVARGLAPLREVQAEVQQRSARHLQPIAVEAVPLEIRGLIDELNLLLERLRTALEAE**

11 UTDF19-28A 100.0% 99.0% **LLLFYSLFPLLLALPLLGGLVWFGVARGLAPLREVQAEVQQRSARHLQPIAVEAVPLEIRGLIDELNLLLERLRTALEAE**

12 CMPL223 100.0% 99.0% **LLLFYSLFPLLLALPLLGGLVWFGVARGLAPLREVQAEVQQRSARHLQPIAVEAVPLEIRGLIDELNLLLERLRTALEAE**

13 AS_23 100.0% 100.0% **LLLFYSLFPLLLALPLLGGLVWFGVARGLAPLREVQAEVQQRSARHLQPIAVEAVPLEIRGLIDELNLLLERLRTALEAE**

14 PS1972 100.0% 99.2% **LLLFYSLFPLLLALPLLGGLVWFGVARGLAPLREVQAEVQQRSARHLQPIAVEAVPLEIRGLIDELNLLLERLRTALEAE**

15 BA10747 100.0% 100.0% **LLLFYSLFPLLLALPLLGGLVWFGVARGLAPLREVQAEVQQRSARHLQPIAVEAVPLEIRGLIDELNLLLERLRTALEAE**

16 ST773 100.0% 99.6% **LLLFYSLFPLLLALPLLGGLVWFGVARGLAPLREVQAEVQQRSARHLQPIAVEAVPLEIRGLIDELNLLLERLRTALEAE**

17 DSM50071 100.0% 99.2% **LLLFYSLFPLLLALPLLGGLVWFGVARGLAPLREVQAEVQQRSARHLQPIAVEAVPLEIRGLIDELNLLLERLRTALEAE**

18 XDR-PA 100.0% 99.2% **LLLFYSLFPLLLALPLLGGLVWFGVARGLAPLREVQAEVQQRSARHLQPIAVEAVPLEIRGLIDELNLLLERLRTALEAE**

consensus/100% **LLLFYSLFPLLLALPLLGGLVWFGVARGLAPLREVQAEVQQRSARHLQPIAVEAVPLEIRGLIDELNLLLERLRTALEAE**

consensus/90% **LLLFYSLFPLLLALPLLGGLVWFGVARGLAPLREVQAEVQQRSARHLQPIAVEAVPLEIRGLIDELNLLLERLRTALEAE**

consensus/80% **LLLFYSLFPLLLALPLLGGLVWFGVARGLAPLREVQAEVQQRSARHLQPIAVEAVPLEIRGLIDELNLLLERLRTALEAE**

consensus/70% **LLLFYSLFPLLLALPLLGGLVWFGVARGLAPLREVQAEVQQRSARHLQPIAVEAVPLEIRGLIDELNLLLERLRTALEAE**

cov pid **241**  **: . . . . 3 . .** **320**

1 DFU7 100.0% 100.0% **RRLTSDAAHEIRTPLASLRTHAQVALRSEDPKAHARGLLQVSRSVERISTLMEQILLLARLDGDALLEQFHPVNLATLAE**

2 DFU9 100.0% 99.0% **RRLTSDAAHEIRTPLASLRTHAQVALRSEDPKAHARGLLQVSRSVERISTLMEQILLLARLDGDALLEQFHPVNLATLAE**

3 DFU16 100.0% 99.0% **RRLTSDAAHEIRTPLASLRTHAQVALRSEDPKAHARGLLQVSRSVERISTLMEQILLLARLDGDALLEQFHPVNLATLAE**

4 DFU48 100.0% 100.0% **RRLTSDAAHEIRTPLASLRTHAQVALRSEDPKAHARGLLQVSRSVERISTLMEQILLLARLDGDALLEQFHPVNLATLAE**

5 DFU58 100.0% 99.2% **RRLTSDAAHEIRTPLASLRTHAQVALRSEDPKAHARGLLQVSRSVERISTLMEQILLLARLDGDALLEQFHPVNLATLAE**

6 PAO1 100.0% 99.0% **RRLTSDAAHEIRTPLASLRTHAQVALRSEDPKAHARGLLQVSRSVERISTLMEQILLLARLDGDALLEQFHPVNLATLAE**

7 ATCC33363 100.0% 99.2% **RRLTSDAAHEIRTPLASLRTHAQVALRSEDPKAHARGLLQVSRSVERISTLMEQILLLARLDGDALLEQFHPVNLATLAE**

8 ZBX-P25 100.0% 99.2% **RRLTSDAAHEIRTPLASLRTHAQVALRSEDPKAHARGLLQVSRSVERISTLMEQILLLARLDGDALLEQFHPVNLATLAE**

9 DMC-20C 100.0% 99.2% **RRLTSDAAHEIRTPLASLRTHAQVALRSEDPKAHARGLLQVSRSVERISTLMEQILLLARLDGDALLEQFHPVNLATLAE**

10 P8W 100.0% 99.2% **RRLTSDAAHEIRTPLASLRTHAQVALRSEDPKAHARGLLQVSRSVERISTLMEQILLLARLDGDALLEQFHPVNLATLAE**

11 UTDF19-28A 100.0% 99.0% **RRLTSDAAHEIRTPLASLRTHAQVALRSEDPKAHARGLLQVSRSVERISTLMEQILLLARLDGDALLEQFHPVNLATLAE**

12 CMPL223 100.0% 99.0% **RRLTSDAAHEIRTPLASLRTHAQVALRSEDPKAHARGLLQVSRSVERISTLMEQILLLARLDGDALLEQFHPVNLATLAE**

13 AS_23 100.0% 100.0% **RRLTSDAAHEIRTPLASLRTHAQVALRSEDPKAHARGLLQVSRSVERISTLMEQILLLARLDGDALLEQFHPVNLATLAE**

14 PS1972 100.0% 99.2% **RRLTSDAAHEIRTPLASLRTHAQVALRSEDPKAHARGLLQVSRSVERISTLMEQILLLARLDGDALLEQFHPVNLATLAE**

15 BA10747 100.0% 100.0% **RRLTSDAAHEIRTPLASLRTHAQVALRSEDPKAHARGLLQVSRSVERISTLMEQILLLARLDGDALLEQFHPVNLATLAE**

16 ST773 100.0% 99.6% **RRLTSDAAHEIRTPLASLRPHAQVALRSEDPKAHARGLLQVSRSVERISTLMEQILLLARLDGDALLEQFHPVNLATLAE**

17 DSM50071 100.0% 99.2% **RRLTSDAAHEIRTPLASLRTHAQVALRSEDPKAHARGLLQVSRSVERISTLMEQILLLARLDGDALLEQFHPVNLATLAE**

18 XDR-PA 100.0% 99.2% **RRLTSDAAHEIRTPLASLRTHAQVALRSEDPKAHARGLLQVSRSVERISTLMEQILLLARLDGDALLEQFHPVNLATLAE**

consensus/100% **RRLTSDAAHEIRTPLASLRsHAQVALRSEDPKAHARGLLQVSRSVERISTLMEQILLLARLDGDALLEQFHPVNLATLAE**

consensus/90% **RRLTSDAAHEIRTPLASLRTHAQVALRSEDPKAHARGLLQVSRSVERISTLMEQILLLARLDGDALLEQFHPVNLATLAE**

consensus/80% **RRLTSDAAHEIRTPLASLRTHAQVALRSEDPKAHARGLLQVSRSVERISTLMEQILLLARLDGDALLEQFHPVNLATLAE**

consensus/70% **RRLTSDAAHEIRTPLASLRTHAQVALRSEDPKAHARGLLQVSRSVERISTLMEQILLLARLDGDALLEQFHPVNLATLAE**

cov pid **321**  **. . : . . . . 4** **400**

1 DFU7 100.0% 100.0% **DVLSELARQAIDKDIELSLHQETVHVMGIDLWLKAMVGNLVGNALRYTPAGGQVEIRVENRAQHAVLRVRDNGPGVALEE**

2 DFU9 100.0% 99.0% **DVLSELARQAIDKDIELSLHQETVYVMGIDLWLKAMVGNLVGNALRYTPAGGQVEIRVENRAQHAVLRVRDNGPGVALEE**

3 DFU16 100.0% 99.0% **DVLSELARQAIDKDIELSLHQETVYVMGIDLWLKAMVGNLVGNALRYTPAGGQVEIRVENRAQHAVLRVRDNGPGVALEE**

4 DFU48 100.0% 100.0% **DVLSELARQAIDKDIELSLHQETVHVMGIDLWLKAMVGNLVGNALRYTPAGGQVEIRVENRAQHAVLRVRDNGPGVALEE**

5 DFU58 100.0% 99.2% **DVLSELARQAIDKDIELSLHQETVHVMGIDLWLKAMVGNLVGNALRYTPAGGQVEIRVENRAQHAVLRVRDNGPGVALEE**

6 PAO1 100.0% 99.0% **DVLSELARQAIDKDIELSLHQETVYVMGIDLWLKAMVGNLVGNALRYTPAGGQVEIRVENRAQHAVLRVRDNGPGVALEE**

7 ATCC33363 100.0% 99.2% **DVLSELARQAIDKDIELSLHQETVHVMGIDLWLKAMVGNLVGNALRYTPAGGQVEIRVENRAQHAVLRVRDNGPGVALEE**

8 ZBX-P25 100.0% 99.2% **DVLSELARQAIDKDIELSLHQETVHVMGIDLWLKAMVGNLVGNALRYTPAGGQVEIRVENRAQHAVLRVRDNGPGVALEE**

9 DMC-20C 100.0% 99.2% **DVLSELARQAIDKDIELSLHQETVHVMGIDLWLKAMVGNLVGNALRYTPAGGQVEIRVENRAQHAVLRVRDNGPGVALEE**

10 P8W 100.0% 99.2% **DVLSELARQAIDKDIELSLHQETVHVMGIDLWLKAMVGNLVGNALRYTPAGGQVEIRVENRAQHAVLRVRDNGPGVALEE**

11 UTDF19-28A 100.0% 99.0% **DVLSELARQAIDKDIELSLHQETVHVMGIDLWLKAMVGNLVGNALRYTPAGGQVEIRVENRAQHAVLRVRDNGPGVALEE**

12 CMPL223 100.0% 99.0% **DVLSELARQAIDKDIELSLHQETVHVMGIDLWLKAMVGNLVGNALRYTPAGGQVEIRVENRAQHAVLRVRDNGPGVALEE**

13 AS_23 100.0% 100.0% **DVLSELARQAIDKDIELSLHQETVHVMGIDLWLKAMVGNLVGNALRYTPAGGQVEIRVENRAQHAVLRVRDNGPGVALEE**

14 PS1972 100.0% 99.2% **DVLSELARQAIDKDIELSLHQETVHVMGIDLWLKAMVGNLVGNALRYTPAGGQVEIRVENRAQHAVLRVRDNGPGVALEE**

15 BA10747 100.0% 100.0% **DVLSELARQAIDKDIELSLHQETVHVMGIDLWLKAMVGNLVGNALRYTPAGGQVEIRVENRAQHAVLRVRDNGPGVALEE**

16 ST773 100.0% 99.6% **DVLSELARQAIDKDIELSLHQETVHVMGIDLWLKAMVGNLVGNALRYTPAGGQVEIRVENRAQHAVLRVRDNGPGVALEE**

17 DSM50071 100.0% 99.2% **DVLSELARQAIDKDIELSLHQETVHVMGIDLWLKAMVGNLVGNALRYTPAGGQVEIRVENRAQHAVLRVRDNGPGVALEE**

18 XDR-PA 100.0% 99.2% **DVLSELARQAIDKDIELSLHQETVHVMGIDLWLKAMVGNLVGNALRYTPAGGQVEIRVENRAQHAVLRVRDNGPGVALEE**

consensus/100% **DVLSELARQAIDKDIELSLHQETVaVMGIDLWLKAMVGNLVGNALRYTPAGGQVEIRVENRAQHAVLRVRDNGPGVALEE**

consensus/90% **DVLSELARQAIDKDIELSLHQETVaVMGIDLWLKAMVGNLVGNALRYTPAGGQVEIRVENRAQHAVLRVRDNGPGVALEE**

consensus/80% **DVLSELARQAIDKDIELSLHQETVHVMGIDLWLKAMVGNLVGNALRYTPAGGQVEIRVENRAQHAVLRVRDNGPGVALEE**

consensus/70% **DVLSELARQAIDKDIELSLHQETVHVMGIDLWLKAMVGNLVGNALRYTPAGGQVEIRVENRAQHAVLRVRDNGPGVALEE**

cov pid **401**  **. . . . : . . ]** **477**

1 DFU7 100.0% 100.0% **QQAIFTRFYRSPATSSGEGSGLGLPIVKRIVELHFGSIGLGKGLEGKGLEVQVFLPKTQPDATRPPARGPDSGRSHI**

2 DFU9 100.0% 99.0% **QQAIFTRFYRSPATSSGEGSGLGLPIVKRIVELHFGSIGLGKGLEGKGLEVQVFLPKTQPDATRPPARGPDSGRSHI**

3 DFU16 100.0% 99.0% **QQAIFTRFYRSPATSSGEGSGLGLPIVKRIVELHFGSIGLGKGLEGKGLEVQVFLPKTQPDATRPPARGPDSGRSHI**

4 DFU48 100.0% 100.0% **QQAIFTRFYRSPATSSGEGSGLGLPIVKRIVELHFGSIGLGKGLEGKGLEVQVFLPKTQPDATRPPARGPDSGRSHI**

5 DFU58 100.0% 99.2% **QQAIFTRFYRSPATSSGEGSGLGLPIVKRIVELHFGSIGLGKGLEGKGLEVQVFLPKTQPDATRPPARGPDSGRSHI**

6 PAO1 100.0% 99.0% **QQAIFTRFYRSPATSSGEGSGLGLPIVKRIVELHFGSIGLGKGLEGKGLEVQVFLPKTQPDATRPPARGPDSGRSHI**

7 ATCC33363 100.0% 99.2% **QQAIFTRFYRSPATSSGEGSGLGLPIVKRIVELHFGSIGLGKGLEGKGLEVQVFLPKTQPDATRPPARGPDSGRSHI**

8 ZBX-P25 100.0% 99.2% **QQAIFTRFYRSPATSSGEGSGLGLPIVKRIVELHFGSIGLGKGLEGKGLEVQVFLPKTQPDATRPPARGPDSGRSHI**

9 DMC-20C 100.0% 99.2% **QQAIFTRFYRSPATSSGEGSGLGLPIVKRIVELHFGSIGLGKGLEGKGLEVQVFLPKTQPDATRPPARGPDSGRSHI**

10 P8W 100.0% 99.2% **QQAIFTRFYRSPATSSGEGSGLGLPIVKRIVELHFGSIGLGKGLEGKGLEVQVFLPKTQPDATRPPARGPDSGRSHI**

11 UTDF19-28A 100.0% 99.0% **QQAIFTRFYRSPATSSGEGSGLGLPIVKRIVELHFGSIGLGKGLEGKGLEVQVFLPKTQPDTTRPPARGPDSGRSHI**

12 CMPL223 100.0% 99.0% **QQAIFTRFYRSPATSSGEGSGLGLPIVKRIVELHFGSIGLGKGLEGKGLEVQVFLPKTQPDATRPPARGPDSGRSHI**

13 AS_23 100.0% 100.0% **QQAIFTRFYRSPATSSGEGSGLGLPIVKRIVELHFGSIGLGKGLEGKGLEVQVFLPKTQPDATRPPARGPDSGRSHI**

14 PS1972 100.0% 99.2% **QQAIFTRFYRSPATSSGEGSGLGLPIVKRIVELHFGSIGLGKGLEGKGLEVQVFLPKTQPDATRPPARGPDSGRSHI**

15 BA10747 100.0% 100.0% **QQAIFTRFYRSPATSSGEGSGLGLPIVKRIVELHFGSIGLGKGLEGKGLEVQVFLPKTQPDATRPPARGPDSGRSHI**

16 ST773 100.0% 99.6% **QQAIFTRFYRSPATSSGEGSGLGLPIVKRIVELHFGSIGLGKGLEGKGLEVQVFLPKTQPDATRPPARGPDSGRSHI**

17 DSM50071 100.0% 99.2% **QQAIFTRFYRSPATSSGEGSGLGLPIVKRIVELHFGSIGLGKGLEGKGLEVQVFLPKTQPDATRPPARGPDSGRSHI**

18 XDR-PA 100.0% 99.2% **QQAIFTRFYRSPATSSGEGSGLGLPIVKRIVELHFGSIGLGKGLEGKGLEVQVFLPKTQPDATRPPARGPDSGRSHI**

consensus/100% **QQAIFTRFYRSPATSSGEGSGLGLPIVKRIVELHFGSIGLGKGLEGKGLEVQVFLPKTQPDsTRPPARGPDSGRSHI**

consensus/90% **QQAIFTRFYRSPATSSGEGSGLGLPIVKRIVELHFGSIGLGKGLEGKGLEVQVFLPKTQPDATRPPARGPDSGRSHI**

consensus/80% **QQAIFTRFYRSPATSSGEGSGLGLPIVKRIVELHFGSIGLGKGLEGKGLEVQVFLPKTQPDATRPPARGPDSGRSHI**

consensus/70% **QQAIFTRFYRSPATSSGEGSGLGLPIVKRIVELHFGSIGLGKGLEGKGLEVQVFLPKTQPDATRPPARGPDSGRSHI**

**Supplementary Figure 5.** Multiple sequence alignment of the predicted amino acid sequences of PmrB (BasS) carried by P. aeruginosa isolates and close genomes retrieved from the BV-BRC database compared to that P. aeruginosa strains (PAO1 and ATCC^®^33363). The alignment was visualized using MView version 1.63 hosted by the EMBL-EBI; cov, coverage; pid, percent identity.

cov pid  **1** **[ . . . . : . . .** **80**

1 DFU7 100.0% 100.0% **MIRSLRIRLMLGAAALAVLFMLALLPALQRAFGIALENTIEQRLAADVATLVSAARVEKGRLVMPEHLPVEEFNLPEAKV**

2 DFU9 100.0% 100.0% **MIRSLRIRLMLGAAALAVLFMLALLPALQRAFGIALENTIEQRLAADVATLVSAARVEKGRLVMPEHLPVEEFNLPEAKV**

3 DFU16 100.0% 100.0% **MIRSLRIRLMLGAAALAVLFMLALLPALQRAFGIALENTIEQRLAADVATLVSAARVEKGRLVMPEHLPVEEFNLPEAKV**

4 DFU48 100.0% 100.0% **MIRSLRIRLMLGAAALAVLFMLALLPALQRAFGIALENTIEQRLAADVATLVSAARVEKGRLVMPEHLPVEEFNLPEAKV**

5 DFU58 100.0% 100.0% **MIRSLRIRLMLGAAALAVLFMLALLPALQRAFGIALENTIEQRLAADVATLVSAARVEKGRLVMPEHLPVEEFNLPEAKV**

6 PAO1 100.0% 100.0% **MIRSLRIRLMLGAAALAVLFMLALLPALQRAFGIALENTIEQRLAADVATLVSAARVEKGRLVMPEHLPVEEFNLPEAKV**

7 ATCC33363 100.0% 100.0% **MIRSLRIRLMLGAAALAVLFMLALLPALQRAFGIALENTIEQRLAADVATLVSAARVEKGRLVMPEHLPVEEFNLPEAKV**

8 ZBX-P25 100.0% 100.0% **MIRSLRIRLMLGAAALAVLFMLALLPALQRAFGIALENTIEQRLAADVATLVSAARVEKGRLVMPEHLPVEEFNLPEAKV**

9 DMC-20C 100.0% 100.0% **MIRSLRIRLMLGAAALAVLFMLALLPALQRAFGIALENTIEQRLAADVATLVSAARVEKGRLVMPEHLPVEEFNLPEAKV**

10 P8W 100.0% 100.0% **MIRSLRIRLMLGAAALAVLFMLALLPALQRAFGIALENTIEQRLAADVATLVSAARVEKGRLVMPEHLPVEEFNLPEAKV**

11 UTDF19-28A 100.0% 100.0% **MIRSLRIRLMLGAAALAVLFMLALLPALQRAFGIALENTIEQRLAADVATLVSAARVEKGRLVMPEHLPVEEFNLPEAKV**

12 CMPL223 100.0% 100.0% **MIRSLRIRLMLGAAALAVLFMLALLPALQRAFGIALENTIEQRLAADVATLVSAARVEKGRLVMPEHLPVEEFNLPEAKV**

13 AS_23 100.0% 100.0% **MIRSLRIRLMLGAAALAVLFMLALLPALQRAFGIALENTIEQRLAADVATLVSAARVEKGRLVMPEHLPVEEFNLPEAKV**

14 PS1972 100.0% 100.0% **MIRSLRIRLMLGAAALAVLFMLALLPALQRAFGIALENTIEQRLAADVATLVSAARVEKGRLVMPEHLPVEEFNLPEAKV**

15 BA10747 100.0% 100.0% **MIRSLRIRLMLGAAALAVLFMLALLPALQRAFGIALENTIEQRLAADVATLVSAARVEKGRLVMPEHLPVEEFNLPEAKV**

16 PSA10 100.0% 100.0% **MIRSLRIRLMLGAAALAVLFMLALLPALQRAFGIALENTIEQRLAADVATLVSAARVEKGRLVMPEHLPVEEFNLPEAKV**

17 ST773 100.0% 100.0% **MIRSLRIRLMLGAAALAVLFMLALLPALQRAFGIALENTIEQRLAADVATLVSAARVEKGRLVMPEHLPVEEFNLPEAKV**

18 DSM50071 100.0% 100.0% **MIRSLRIRLMLGAAALAVLFMLALLPALQRAFGIALENTIEQRLAADVATLVSAARVEKGRLVMPEHLPVEEFNLPEAKV**

19 XDR-PA 100.0% 100.0% **MIRSLRIRLMLGAAALAVLFMLALLPALQRAFGIALENTIEQRLAADVATLVSAARVEKGRLVMPEHLPVEEFNLPEAKV**

consensus/100% **MIRSLRIRLMLGAAALAVLFMLALLPALQRAFGIALENTIEQRLAADVATLVSAARVEKGRLVMPEHLPVEEFNLPEAKV**

consensus/90% **MIRSLRIRLMLGAAALAVLFMLALLPALQRAFGIALENTIEQRLAADVATLVSAARVEKGRLVMPEHLPVEEFNLPEAKV**

consensus/80% **MIRSLRIRLMLGAAALAVLFMLALLPALQRAFGIALENTIEQRLAADVATLVSAARVEKGRLVMPEHLPVEEFNLPEAKV**

consensus/70% **MIRSLRIRLMLGAAALAVLFMLALLPALQRAFGIALENTIEQRLAADVATLVSAARVEKGRLVMPEHLPVEEFNLPEAKV**

cov pid  **81**  **. 1 . . . . : .** **160**

1 DFU7 100.0% 100.0% **LGYIYDQNGDLLWRSTSAADESINYTPRYDGRGNEFHTTRDAKGEEFFVFDVEIDLLRGKQAAYSIVTMQSVSEFESLLK**

2 DFU9 100.0% 100.0% **LGYIYDQNGDLLWRSTSAADESINYTPRYDGRGNEFHTTRDAKGEEFFVFDVEIDLLRGKQAAYSIVTMQSVSEFESLLK**

3 DFU16 100.0% 100.0% **LGYIYDQNGDLLWRSTSAADESINYTPRYDGRGNEFHTTRDAKGEEFFVFDVEIDLLRGKQAAYSIVTMQSVSEFESLLK**

4 DFU48 100.0% 100.0% **LGYIYDQNGDLLWRSTSAADESINYTPRYDGRGNEFHTTRDAKGEEFFVFDVEIDLLRGKQAAYSIVTMQSVSEFESLLK**

5 DFU58 100.0% 100.0% **LGYIYDQNGDLLWRSTSAADESINYTPRYDGRGNEFHTTRDAKGEEFFVFDVEIDLLRGKQAAYSIVTMQSVSEFESLLK**

6 PAO1 100.0% 100.0% **LGYIYDQNGDLLWRSTSAADESINYTPRYDGRGNEFHTTRDAKGEEFFVFDVEIDLLRGKQAAYSIVTMQSVSEFESLLK**

7 ATCC33363 100.0% 100.0% **LGYIYDQNGDLLWRSTSAADESINYTPRYDGRGNEFHTTRDAKGEEFFVFDVEIDLLRGKQAAYSIVTMQSVSEFESLLK**

8 ZBX-P25 100.0% 100.0% **LGYIYDQNGDLLWRSTSAADESINYTPRYDGRGNEFHTTRDAKGEEFFVFDVEIDLLRGKQAAYSIVTMQSVSEFESLLK**

9 DMC-20C 100.0% 100.0% **LGYIYDQNGDLLWRSTSAADESINYTPRYDGRGNEFHTTRDAKGEEFFVFDVEIDLLRGKQAAYSIVTMQSVSEFESLLK**

10 P8W 100.0% 100.0% **LGYIYDQNGDLLWRSTSAADESINYTPRYDGRGNEFHTTRDAKGEEFFVFDVEIDLLRGKQAAYSIVTMQSVSEFESLLK**

11 UTDF19-28A 100.0% 100.0% **LGYIYDQNGDLLWRSTSAADESINYTPRYDGRGNEFHTTRDAKGEEFFVFDVEIDLLRGKQAAYSIVTMQSVSEFESLLK**

12 CMPL223 100.0% 100.0% **LGYIYDQNGDLLWRSTSAADESINYTPRYDGRGNEFHTTRDAKGEEFFVFDVEIDLLRGKQAAYSIVTMQSVSEFESLLK**

13 AS_23 100.0% 100.0% **LGYIYDQNGDLLWRSTSAADESINYTPRYDGRGNEFHTTRDAKGEEFFVFDVEIDLLRGKQAAYSIVTMQSVSEFESLLK**

14 PS1972 100.0% 100.0% **LGYIYDQNGDLLWRSTSAADESINYTPRYDGRGNEFHTTRDAKGEEFFVFDVEIDLLRGKQAAYSIVTMQSVSEFESLLK**

15 BA10747 100.0% 100.0% **LGYIYDQNGDLLWRSTSAADESINYTPRYDGRGNEFHTTRDAKGEEFFVFDVEIDLLRGKQAAYSIVTMQSVSEFESLLK**

16 PSA10 100.0% 100.0% **LGYIYDQNGDLLWRSTSAADESINYTPRYDGRGNEFHTTRDAKGEEFFVFDVEIDLLRGKQAAYSIVTMQSVSEFESLLK**

17 ST773 100.0% 100.0% **LGYIYDQNGDLLWRSTSAADESINYTPRYDGRGNEFHTTRDAKGEEFFVFDVEIDLLRGKQAAYSIVTMQSVSEFESLLK**

18 DSM50071 100.0% 100.0% **LGYIYDQNGDLLWRSTSAADESINYTPRYDGRGNEFHTTRDAKGEEFFVFDVEIDLLRGKQAAYSIVTMQSVSEFESLLK**

19 XDR-PA 100.0% 100.0% **LGYIYDQNGDLLWRSTSAADESINYTPRYDGRGNEFHTTRDAKGEEFFVFDVEIDLLRGKQAAYSIVTMQSVSEFESLLK**

consensus/100% **LGYIYDQNGDLLWRSTSAADESINYTPRYDGRGNEFHTTRDAKGEEFFVFDVEIDLLRGKQAAYSIVTMQSVSEFESLLK**

consensus/90% **LGYIYDQNGDLLWRSTSAADESINYTPRYDGRGNEFHTTRDAKGEEFFVFDVEIDLLRGKQAAYSIVTMQSVSEFESLLK**

consensus/80% **LGYIYDQNGDLLWRSTSAADESINYTPRYDGRGNEFHTTRDAKGEEFFVFDVEIDLLRGKQAAYSIVTMQSVSEFESLLK**

consensus/70% **LGYIYDQNGDLLWRSTSAADESINYTPRYDGRGNEFHTTRDAKGEEFFVFDVEIDLLRGKQAAYSIVTMQSVSEFESLLK**

cov pid **161**  **. . . 2 . . . .** **240**

1 DFU7 100.0% 100.0% **GFREQLYLWLGGALLVLLGLLWLGLTWGFRAMRGLSSELDQIESGERESLSEEHPRELLRLTHSLNRLLRSEHKQRERYR**

2 DFU9 100.0% 100.0% **GFREQLYLWLGGALLVLLGLLWLGLTWGFRAMRGLSSELDQIESGERESLSEEHPRELLRLTHSLNRLLRSEHKQRERYR**

3 DFU16 100.0% 100.0% **GFREQLYLWLGGALLVLLGLLWLGLTWGFRAMRGLSSELDQIESGERESLSEEHPRELLRLTHSLNRLLRSEHKQRERYR**

4 DFU48 100.0% 100.0% **GFREQLYLWLGGALLVLLGLLWLGLTWGFRAMRGLSSELDQIESGERESLSEEHPRELLRLTHSLNRLLRSEHKQRERYR**

5 DFU58 100.0% 100.0% **GFREQLYLWLGGALLVLLGLLWLGLTWGFRAMRGLSSELDQIESGERESLSEEHPRELLRLTHSLNRLLRSEHKQRERYR**

6 PAO1 100.0% 100.0% **GFREQLYLWLGGALLVLLGLLWLGLTWGFRAMRGLSSELDQIESGERESLSEEHPRELLRLTHSLNRLLRSEHKQRERYR**

7 ATCC33363 100.0% 100.0% **GFREQLYLWLGGALLVLLGLLWLGLTWGFRAMRGLSSELDQIESGERESLSEEHPRELLRLTHSLNRLLRSEHKQRERYR**

8 ZBX-P25 100.0% 100.0% **GFREQLYLWLGGALLVLLGLLWLGLTWGFRAMRGLSSELDQIESGERESLSEEHPRELLRLTHSLNRLLRSEHKQRERYR**

9 DMC-20C 100.0% 100.0% **GFREQLYLWLGGALLVLLGLLWLGLTWGFRAMRGLSSELDQIESGERESLSEEHPRELLRLTHSLNRLLRSEHKQRERYR**

10 P8W 100.0% 100.0% **GFREQLYLWLGGALLVLLGLLWLGLTWGFRAMRGLSSELDQIESGERESLSEEHPRELLRLTHSLNRLLRSEHKQRERYR**

11 UTDF19-28A 100.0% 100.0% **GFREQLYLWLGGALLVLLGLLWLGLTWGFRAMRGLSSELDQIESGERESLSEEHPRELLRLTHSLNRLLRSEHKQRERYR**

12 CMPL223 100.0% 100.0% **GFREQLYLWLGGALLVLLGLLWLGLTWGFRAMRGLSSELDQIESGERESLSEEHPRELLRLTHSLNRLLRSEHKQRERYR**

13 AS_23 100.0% 100.0% **GFREQLYLWLGGALLVLLGLLWLGLTWGFRAMRGLSSELDQIESGERESLSEEHPRELLRLTHSLNRLLRSEHKQRERYR**

14 PS1972 100.0% 100.0% **GFREQLYLWLGGALLVLLGLLWLGLTWGFRAMRGLSSELDQIESGERESLSEEHPRELLRLTHSLNRLLRSEHKQRERYR**

15 BA10747 100.0% 100.0% **GFREQLYLWLGGALLVLLGLLWLGLTWGFRAMRGLSSELDQIESGERESLSEEHPRELLRLTHSLNRLLRSEHKQRERYR**

16 PSA10 100.0% 100.0% **GFREQLYLWLGGALLVLLGLLWLGLTWGFRAMRGLSSELDQIESGERESLSEEHPRELLRLTHSLNRLLRSEHKQRERYR**

17 ST773 100.0% 100.0% **GFREQLYLWLGGALLVLLGLLWLGLTWGFRAMRGLSSELDQIESGERESLSEEHPRELLRLTHSLNRLLRSEHKQRERYR**

18 DSM50071 100.0% 100.0% **GFREQLYLWLGGALLVLLGLLWLGLTWGFRAMRGLSSELDQIESGERESLSEEHPRELLRLTHSLNRLLRSEHKQRERYR**

19 XDR-PA 100.0% 100.0% **GFREQLYLWLGGALLVLLGLLWLGLTWGFRAMRGLSSELDQIESGERESLSEEHPRELLRLTHSLNRLLRSEHKQRERYR**

consensus/100% **GFREQLYLWLGGALLVLLGLLWLGLTWGFRAMRGLSSELDQIESGERESLSEEHPRELLRLTHSLNRLLRSEHKQRERYR**

consensus/90% **GFREQLYLWLGGALLVLLGLLWLGLTWGFRAMRGLSSELDQIESGERESLSEEHPRELLRLTHSLNRLLRSEHKQRERYR**

consensus/80% **GFREQLYLWLGGALLVLLGLLWLGLTWGFRAMRGLSSELDQIESGERESLSEEHPRELLRLTHSLNRLLRSEHKQRERYR**

consensus/70% **GFREQLYLWLGGALLVLLGLLWLGLTWGFRAMRGLSSELDQIESGERESLSEEHPRELLRLTHSLNRLLRSEHKQRERYR**

cov pid **241**  **: . . . . 3 . .** **320**

1 DFU7 100.0% 100.0% **HSLGDLAHSLKTPLAVLQGVGDQLAEEPGNREQVRVLQGQIERMSQQIGYQLQRASLRKSGLVRHREQLAPLVETLCDAL**

2 DFU9 100.0% 100.0% **HSLGDLAHSLKTPLAVLQGVGDQLAEEPGNREQVRVLQGQIERMSQQIGYQLQRASLRKSGLVRHREQLAPLVETLCDAL**

3 DFU16 100.0% 100.0% **HSLGDLAHSLKTPLAVLQGVGDQLAEEPGNREQVRVLQGQIERMSQQIGYQLQRASLRKSGLVRHREQLAPLVETLCDAL**

4 DFU48 100.0% 100.0% **HSLGDLAHSLKTPLAVLQGVGDQLAEEPGNREQVRVLQGQIERMSQQIGYQLQRASLRKSGLVRHREQLAPLVETLCDAL**

5 DFU58 100.0% 100.0% **HSLGDLAHSLKTPLAVLQGVGDQLAEEPGNREQVRVLQGQIERMSQQIGYQLQRASLRKSGLVRHREQLAPLVETLCDAL**

6 PAO1 100.0% 100.0% **HSLGDLAHSLKTPLAVLQGVGDQLAEEPGNREQVRVLQGQIERMSQQIGYQLQRASLRKSGLVRHREQLAPLVETLCDAL**

7 ATCC33363 100.0% 100.0% **HSLGDLAHSLKTPLAVLQGVGDQLAEEPGNREQVRVLQGQIERMSQQIGYQLQRASLRKSGLVRHREQLAPLVETLCDAL**

8 ZBX-P25 100.0% 100.0% **HSLGDLAHSLKTPLAVLQGVGDQLAEEPGNREQVRVLQGQIERMSQQIGYQLQRASLRKSGLVRHREQLAPLVETLCDAL**

9 DMC-20C 100.0% 100.0% **HSLGDLAHSLKTPLAVLQGVGDQLAEEPGNREQVRVLQGQIERMSQQIGYQLQRASLRKSGLVRHREQLAPLVETLCDAL**

10 P8W 100.0% 100.0% **HSLGDLAHSLKTPLAVLQGVGDQLAEEPGNREQVRVLQGQIERMSQQIGYQLQRASLRKSGLVRHREQLAPLVETLCDAL**

11 UTDF19-28A 100.0% 100.0% **HSLGDLAHSLKTPLAVLQGVGDQLAEEPGNREQVRVLQGQIERMSQQIGYQLQRASLRKSGLVRHREQLAPLVETLCDAL**

12 CMPL223 100.0% 100.0% **HSLGDLAHSLKTPLAVLQGVGDQLAEEPGNREQVRVLQGQIERMSQQIGYQLQRASLRKSGLVRHREQLAPLVETLCDAL**

13 AS_23 100.0% 100.0% **HSLGDLAHSLKTPLAVLQGVGDQLAEEPGNREQVRVLQGQIERMSQQIGYQLQRASLRKSGLVRHREQLAPLVETLCDAL**

14 PS1972 100.0% 100.0% **HSLGDLAHSLKTPLAVLQGVGDQLAEEPGNREQVRVLQGQIERMSQQIGYQLQRASLRKSGLVRHREQLAPLVETLCDAL**

15 BA10747 100.0% 100.0% **HSLGDLAHSLKTPLAVLQGVGDQLAEEPGNREQVRVLQGQIERMSQQIGYQLQRASLRKSGLVRHREQLAPLVETLCDAL**

16 PSA10 100.0% 100.0% **HSLGDLAHSLKTPLAVLQGVGDQLAEEPGNREQVRVLQGQIERMSQQIGYQLQRASLRKSGLVRHREQLAPLVETLCDAL**

17 ST773 100.0% 100.0% **HSLGDLAHSLKTPLAVLQGVGDQLAEEPGNREQVRVLQGQIERMSQQIGYQLQRASLRKSGLVRHREQLAPLVETLCDAL**

18 DSM50071 100.0% 100.0% **HSLGDLAHSLKTPLAVLQGVGDQLAEEPGNREQVRVLQGQIERMSQQIGYQLQRASLRKSGLVRHREQLAPLVETLCDAL**

19 XDR-PA 100.0% 100.0% **HSLGDLAHSLKTPLAVLQGVGDQLAEEPGNREQVRVLQGQIERMSQQIGYQLQRASLRKSGLVRHREQLAPLVETLCDAL**

consensus/100% **HSLGDLAHSLKTPLAVLQGVGDQLAEEPGNREQVRVLQGQIERMSQQIGYQLQRASLRKSGLVRHREQLAPLVETLCDAL**

consensus/90% **HSLGDLAHSLKTPLAVLQGVGDQLAEEPGNREQVRVLQGQIERMSQQIGYQLQRASLRKSGLVRHREQLAPLVETLCDAL**

consensus/80% **HSLGDLAHSLKTPLAVLQGVGDQLAEEPGNREQVRVLQGQIERMSQQIGYQLQRASLRKSGLVRHREQLAPLVETLCDAL**

consensus/70% **HSLGDLAHSLKTPLAVLQGVGDQLAEEPGNREQVRVLQGQIERMSQQIGYQLQRASLRKSGLVRHREQLAPLVETLCDAL**

cov pid **321**  **. . : . . . . 4** **400**

1 DFU7 100.0% 100.0% **DKVYRDKRVSLQRDFSPSFSVPVERGALLELLGNLLENAYRLCLGRVRVGARLGPGYSELWVEDDGPGVPAEQRARIIRR**

2 DFU9 100.0% 100.0% **DKVYRDKRVSLQRDFSPSFSVPVERGALLELLGNLLENAYRLCLGRVRVGARLGPGYSELWVEDDGPGVPAEQRARIIRR**

3 DFU16 100.0% 100.0% **DKVYRDKRVSLQRDFSPSFSVPVERGALLELLGNLLENAYRLCLGRVRVGARLGPGYSELWVEDDGPGVPAEQRARIIRR**

4 DFU48 100.0% 100.0% **DKVYRDKRVSLQRDFSPSFSVPVERGALLELLGNLLENAYRLCLGRVRVGARLGPGYSELWVEDDGPGVPAEQRARIIRR**

5 DFU58 100.0% 100.0% **DKVYRDKRVSLQRDFSPSFSVPVERGALLELLGNLLENAYRLCLGRVRVGARLGPGYSELWVEDDGPGVPAEQRARIIRR**

6 PAO1 100.0% 100.0% **DKVYRDKRVSLQRDFSPSFSVPVERGALLELLGNLLENAYRLCLGRVRVGARLGPGYSELWVEDDGPGVPAEQRARIIRR**

7 ATCC33363 100.0% 100.0% **DKVYRDKRVSLQRDFSPSFSVPVERGALLELLGNLLENAYRLCLGRVRVGARLGPGYSELWVEDDGPGVPAEQRARIIRR**

8 ZBX-P25 100.0% 100.0% **DKVYRDKRVSLQRDFSPSFSVPVERGALLELLGNLLENAYRLCLGRVRVGARLGPGYSELWVEDDGPGVPAEQRARIIRR**

9 DMC-20C 100.0% 100.0% **DKVYRDKRVSLQRDFSPSFSVPVERGALLELLGNLLENAYRLCLGRVRVGARLGPGYSELWVEDDGPGVPAEQRARIIRR**

10 P8W 100.0% 100.0% **DKVYRDKRVSLQRDFSPSFSVPVERGALLELLGNLLENAYRLCLGRVRVGARLGPGYSELWVEDDGPGVPAEQRARIIRR**

11 UTDF19-28A 100.0% 100.0% **DKVYRDKRVSLQRDFSPSFSVPVERGALLELLGNLLENAYRLCLGRVRVGARLGPGYSELWVEDDGPGVPAEQRARIIRR**

12 CMPL223 100.0% 100.0% **DKVYRDKRVSLQRDFSPSFSVPVERGALLELLGNLLENAYRLCLGRVRVGARLGPGYSELWVEDDGPGVPAEQRARIIRR**

13 AS_23 100.0% 100.0% **DKVYRDKRVSLQRDFSPSFSVPVERGALLELLGNLLENAYRLCLGRVRVGARLGPGYSELWVEDDGPGVPAEQRARIIRR**

14 PS1972 100.0% 100.0% **DKVYRDKRVSLQRDFSPSFSVPVERGALLELLGNLLENAYRLCLGRVRVGARLGPGYSELWVEDDGPGVPAEQRARIIRR**

15 BA10747 100.0% 100.0% **DKVYRDKRVSLQRDFSPSFSVPVERGALLELLGNLLENAYRLCLGRVRVGARLGPGYSELWVEDDGPGVPAEQRARIIRR**

16 PSA10 100.0% 100.0% **DKVYRDKRVSLQRDFSPSFSVPVERGALLELLGNLLENAYRLCLGRVRVGARLGPGYSELWVEDDGPGVPAEQRARIIRR**

17 ST773 100.0% 100.0% **DKVYRDKRVSLQRDFSPSFSVPVERGALLELLGNLLENAYRLCLGRVRVGARLGPGYSELWVEDDGPGVPAEQRARIIRR**

18 DSM50071 100.0% 100.0% **DKVYRDKRVSLQRDFSPSFSVPVERGALLELLGNLLENAYRLCLGRVRVGARLGPGYSELWVEDDGPGVPAEQRARIIRR**

19 XDR-PA 100.0% 100.0% **DKVYRDKRVSLQRDFSPSFSVPVERGALLELLGNLLENAYRLCLGRVRVGARLGPGYSELWVEDDGPGVPAEQRARIIRR**

consensus/100% **DKVYRDKRVSLQRDFSPSFSVPVERGALLELLGNLLENAYRLCLGRVRVGARLGPGYSELWVEDDGPGVPAEQRARIIRR**

consensus/90% **DKVYRDKRVSLQRDFSPSFSVPVERGALLELLGNLLENAYRLCLGRVRVGARLGPGYSELWVEDDGPGVPAEQRARIIRR**

consensus/80% **DKVYRDKRVSLQRDFSPSFSVPVERGALLELLGNLLENAYRLCLGRVRVGARLGPGYSELWVEDDGPGVPAEQRARIIRR**

consensus/70% **DKVYRDKRVSLQRDFSPSFSVPVERGALLELLGNLLENAYRLCLGRVRVGARLGPGYSELWVEDDGPGVPAEQRARIIRR**

cov pid **401**  **. . . . ]** **448**

1 DFU7 100.0% 100.0% **GERADTQHPGQGIGLAVALDIIESYDGELSLDDSELGGACFRIRFATV**

2 DFU9 100.0% 100.0% **GERADTQHPGQGIGLAVALDIIESYDGELSLDDSELGGACFRIRFATV**

3 DFU16 100.0% 100.0% **GERADTQHPGQGIGLAVALDIIESYDGELSLDDSELGGACFRIRFATV**

4 DFU48 100.0% 100.0% **GERADTQHPGQGIGLAVALDIIESYDGELSLDDSELGGACFRIRFATV**

5 DFU58 100.0% 100.0% **GERADTQHPGQGIGLAVALDIIESYDGELSLDDSELGGACFRIRFATV**

6 PAO1 100.0% 100.0% **GERADTQHPGQGIGLAVALDIIESYDGELSLDDSELGGACFRIRFATV**

7 ATCC33363 100.0% 100.0% **GERADTQHPGQGIGLAVALDIIESYDGELSLDDSELGGACFRIRFATV**

8 ZBX-P25 100.0% 100.0% **GERADTQHPGQGIGLAVALDIIESYDGELSLDDSELGGACFRIRFATV**

9 DMC-20C 100.0% 100.0% **GERADTQHPGQGIGLAVALDIIESYDGELSLDDSELGGACFRIRFATV**

10 P8W 100.0% 100.0% **GERADTQHPGQGIGLAVALDIIESYDGELSLDDSELGGACFRIRFATV**

11 UTDF19-28A 100.0% 100.0% **GERADTQHPGQGIGLAVALDIIESYDGELSLDDSELGGACFRIRFATV**

12 CMPL223 100.0% 100.0% **GERADTQHPGQGIGLAVALDIIESYDGELSLDDSELGGACFRIRFATV**

13 AS_23 100.0% 100.0% **GERADTQHPGQGIGLAVALDIIESYDGELSLDDSELGGACFRIRFATV**

14 PS1972 100.0% 100.0% **GERADTQHPGQGIGLAVALDIIESYDGELSLDDSELGGACFRIRFATV**

15 BA10747 100.0% 100.0% **GERADTQHPGQGIGLAVALDIIESYDGELSLDDSELGGACFRIRFATV**

16 PSA10 100.0% 100.0% **GERADTQHPGQGIGLAVALDIIESYDGELSLDDSELGGACFRIRFATV**

17 ST773 100.0% 100.0% **GERADTQHPGQGIGLAVALDIIESYDGELSLDDSELGGACFRIRFATV**

18 DSM50071 100.0% 100.0% **GERADTQHPGQGIGLAVALDIIESYDGELSLDDSELGGACFRIRFATV**

19 XDR-PA 100.0% 100.0% **GERADTQHPGQGIGLAVALDIIESYDGELSLDDSELGGACFRIRFATV**

consensus/100% **GERADTQHPGQGIGLAVALDIIESYDGELSLDDSELGGACFRIRFATV**

consensus/90% **GERADTQHPGQGIGLAVALDIIESYDGELSLDDSELGGACFRIRFATV**

consensus/80% **GERADTQHPGQGIGLAVALDIIESYDGELSLDDSELGGACFRIRFATV**

consensus/70% **GERADTQHPGQGIGLAVALDIIESYDGELSLDDSELGGACFRIRFATV**

**Supplementary Figure 6.** Multiple sequence alignment of the predicted amino acid sequences of PhoQ carried by P. aeruginosa isolates and close genomes retrieved from the BV-BRC database compared to that P. aeruginosa strains (PAO1 and ATCC^®^33363). The alignment was visualized using MView version 1.63 hosted by the EMBL-EBI; cov, coverage; pid, percent identity.


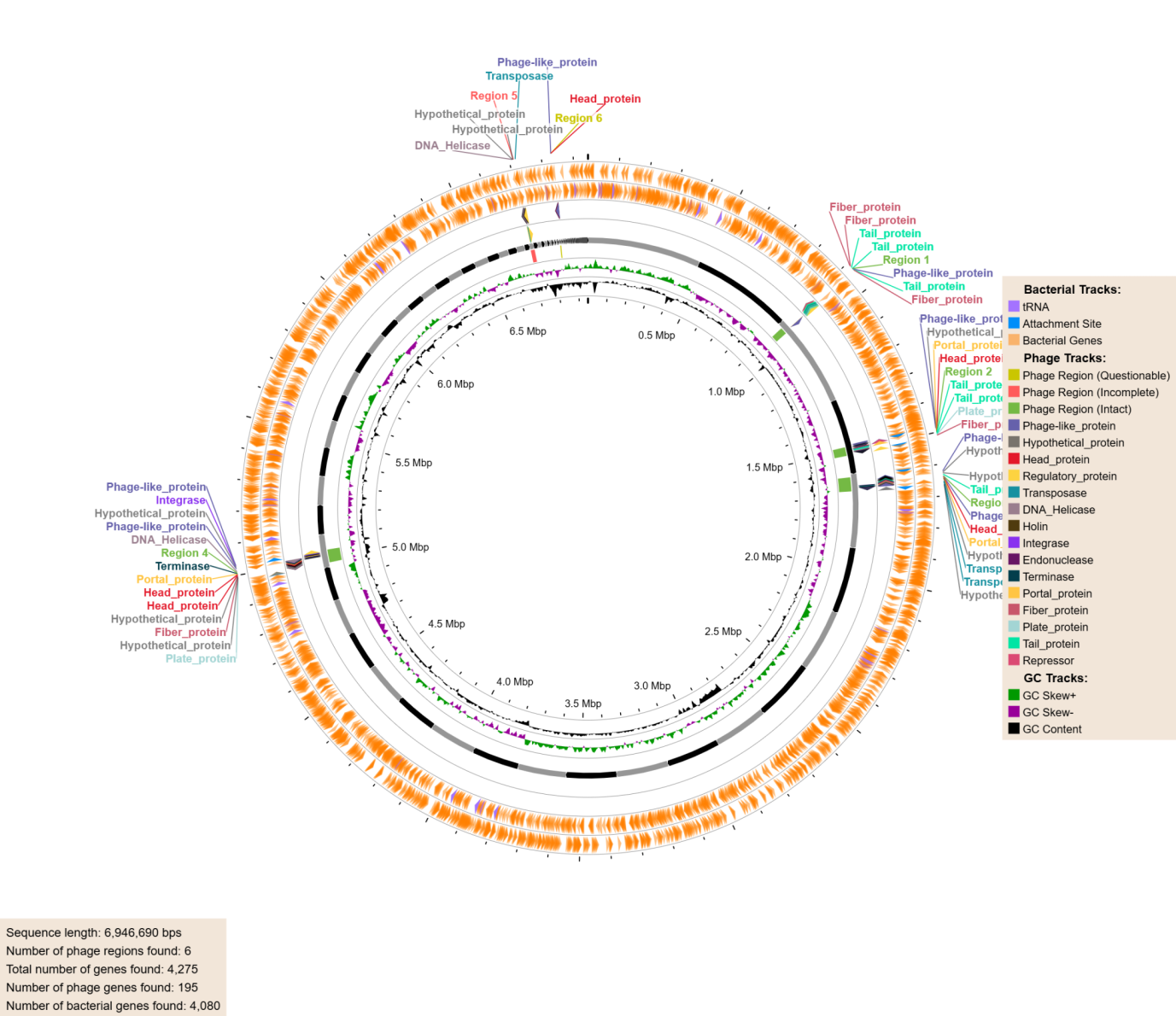


**Supplementary Figure 7. Prophage regions identified in the genome of DFU7**


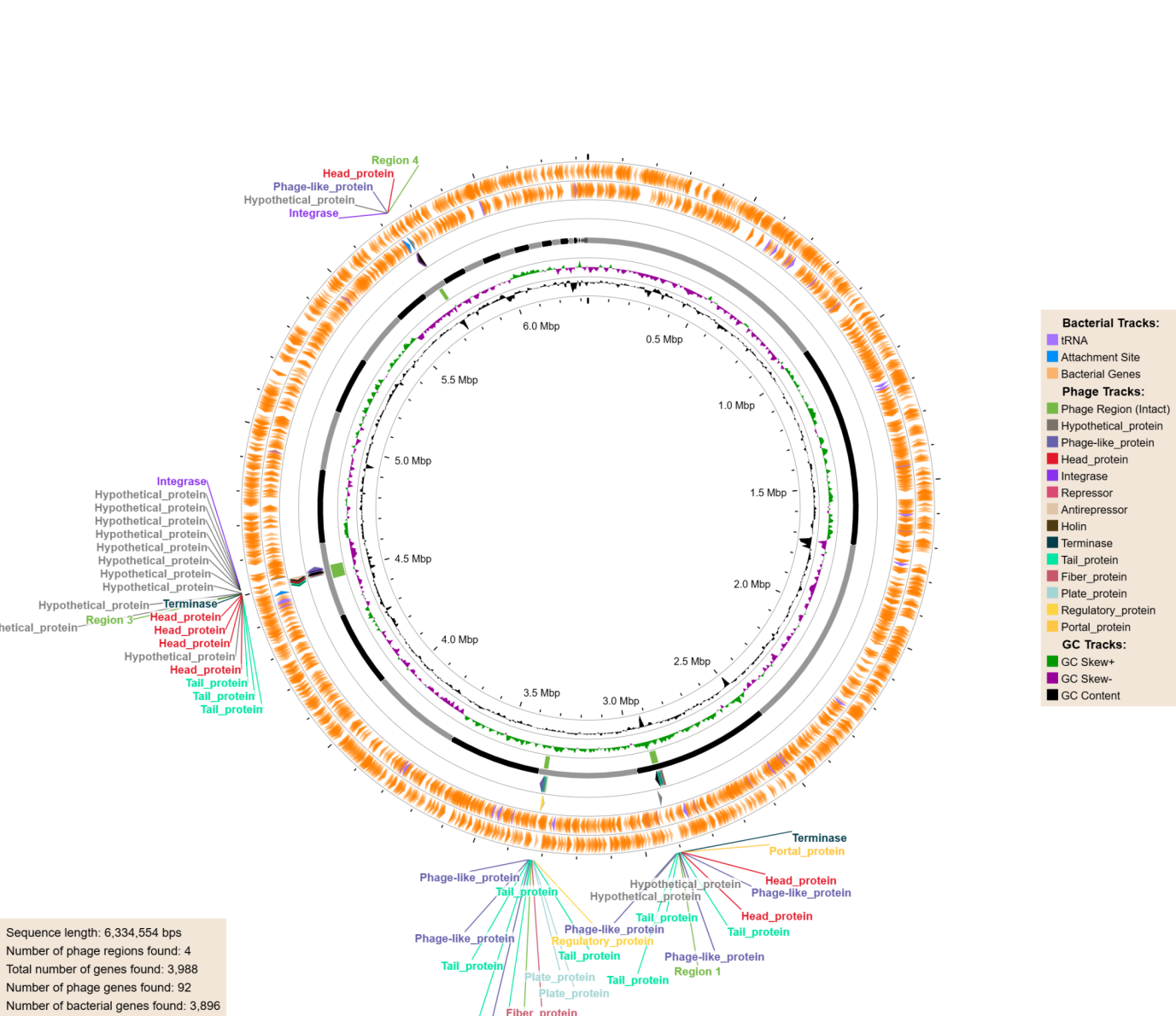


**Supplementary Figure 8. Prophage regions identified in the genome of DFU9**


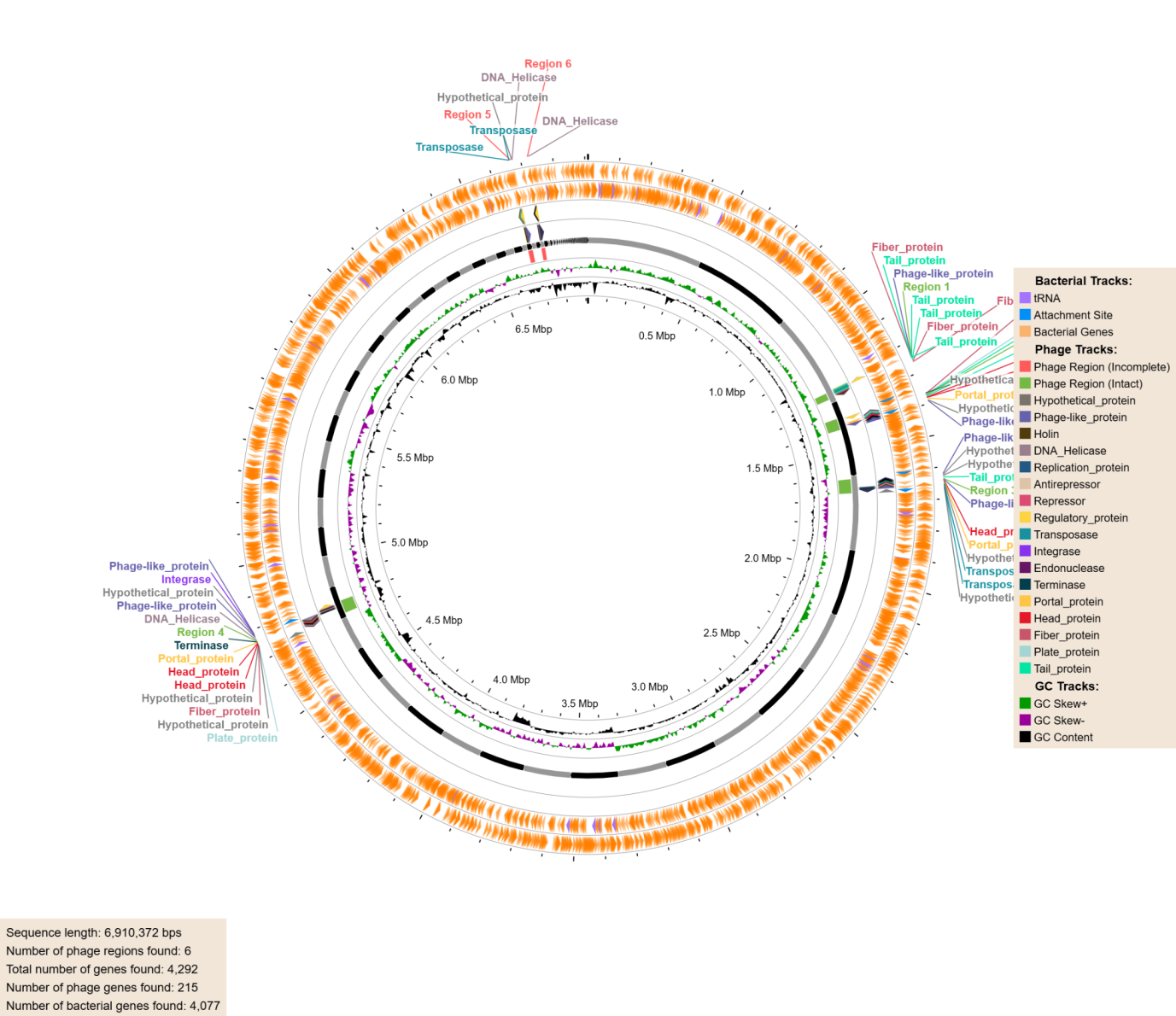


**Supplementary Figure 9. Prophage regions identified in the genome of DFU48**
